# Decarbonylative Transfer Hydrochlorination of Alkenes and Alkynes Based on a B(C_6_F_5_)_3_‐Initiated Grob Fragmentation

**DOI:** 10.1002/anie.202203692

**Published:** 2022-04-11

**Authors:** Kaixue Xie, Martin Oestreich

**Affiliations:** ^1^ Institut für Chemie Technische Universität Berlin Strasse des 17. Juni 115 10623 Berlin Germany

**Keywords:** Alkenes, Boron, Homogeneous Catalysis, Hydrochlorination, Lewis Acids

## Abstract

Readily available cyclohexa‐2,5‐dien‐1‐ylcarbonyl chloride derivatives are introduced as bench‐stable HCl surrogates for transfer hydrochlorination of terminal and internal alkenes as well as selected alkynes. The stepwise Grob fragmentation of those acyl chlorides into chloride, carbon monoxide, a low‐molecular‐weight arene, and a proton is promoted by B(C_6_F_5_)_3_. This decarbonylative transfer process enables the addition of HCl across C−C double and triple bonds with Markovnikov selectivity at room temperature.

## Graphical abstract

B(C_6_F_5_)_3_ initiates the Grob fragmentation of cyclohexa‐2,5‐dien‐1‐yl‐substituted acyl chlorides into a low‐molecular‐weight arene, carbon monoxide and HCl. In the presence of π‐basic substrates such as alkenes and alkynes, HCl is transferred stepwise to afford the corresponding hydrochlorination products. The overall reaction is a decarbonylative transfer hydrochlorination driven by aromatization and carbon‐monoxide release.

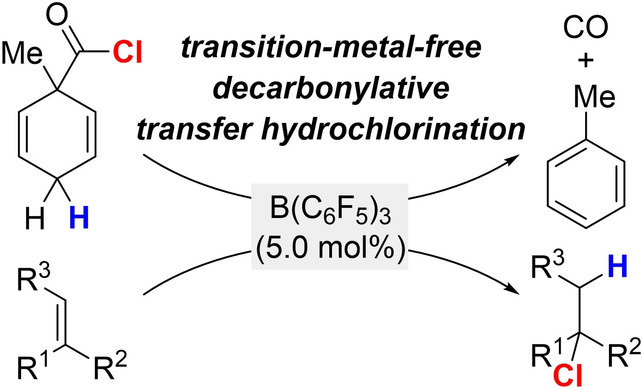

Cyclohexa‐1,4‐diene‐based reagents serve as pro‐aromatic surrogates of mostly gaseous small molecules in ionic^[1]^ and radical^[2]^ transfer processes. The ionic transfer hydrofunctionalization reactions of C−C multiple bonds developed by our laboratory are either catalyzed by boron Lewis acids or Brønsted acids. An example of this is the transfer hydrocyanation of alkenes catalyzed by BCl_3_ or (C_6_F_5_)_2_BCl.^[3]^ The stepwise release of HCN proceeds by Lewis acid‐mediated abstraction of cyanide (nucleofuge) from the surrogate followed by loss of a proton (electrofuge) from the formed Wheland complex. With the cyano group being a pseudohalogen, related transfer hydrohalogenations using surrogates of hydrogen halides seemed within reach (Scheme 1, top left). However, such surrogates with the halogen atom X directly attached to the cyclohexadiene core are not chemically stable because of rapid aromatization. To overcome this predisposition, we introduced reagents with the X group connected to the pro‐aromatic platform by an ethylene tether that would disappear as ethylene gas.^[4]^ These are rather robust HX surrogates that require the use of a strong Brønsted acid such as Tf_2_NH and reaction temperatures of 140 °C or higher to achieve hydroiodination and ‐bromination of C−C triple bonds. The corresponding transfer of HCl failed as a result of stronger C(sp^3^)−Cl bond compared to the C(sp^3^)−I/Br bonds. As an alternative, we recently designed a disguised system that provides two molecules of HCl under the action of B(C_6_F_5_)_3_ by electrocyclic ring opening and β‐elimination at 140 °C (Scheme 1, top right).^[5]^ These forcing reaction conditions have so far thwarted expansion of any of these methods to the transfer hydrohalogenation of alkenes, partly due to product degradation. We therefore seeked other bench‐stable yet more reactive HCl surrogates and were inspired by work of Vorndran and Linker (Scheme 1, middle).^[6]^ These authors reported an acid‐promoted Grob fragmentation of cyclohexa‐2,5‐dien‐1‐ylcarboxylic acid derivatives through the formal intermediacy of an acylium ion (gray box). The idea was to arrive at the same reactive intermediate by boron Lewis acid‐mediated chloride abstraction from the corresponding acyl chloride **2**. Aromatization by the release of carbon monoxide and a proton could then enable the protonation of an alkene **1**; subsequent capture of the resulting carbenium ion by chloride from the previously generated boron ate complex would afford the alkylchloride **3** with Markovnikov selectivity (Scheme 1, bottom).

**Scheme 1:**
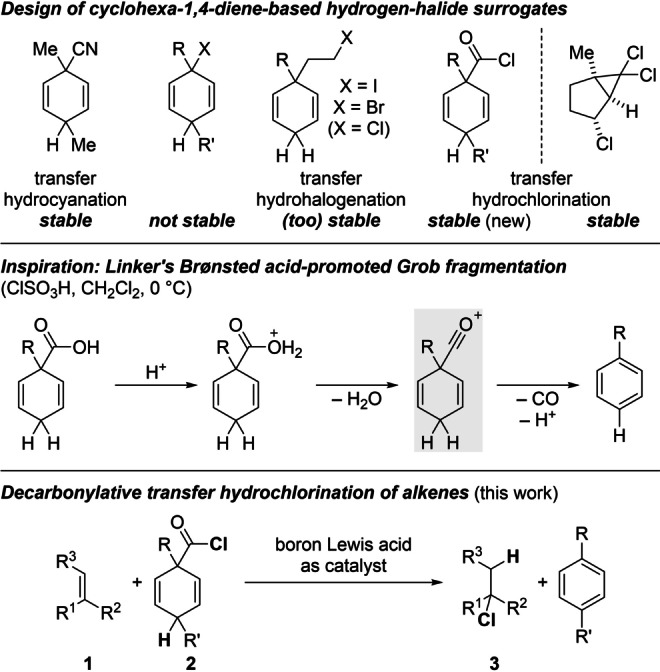
Surrogates for transfer hydro(pseudo)halogenation and design of decarbonylative process. R groups=various aryl and alkyl groups as well as H.

The resulting alkene hydrochlorination is a classic textbook reaction. The direct addition of HCl across C−C double bonds is atom economic but there are safety concerns with the use of toxic and corrosive gaseous HCl or condensed liquid HCl. A limited substrate scope further restricts practicality.^[7]^ Several approaches relying on the in situ generation of HCl were developed^[8]^ yet α‐olefins have remained challenging substrates, and there are problems with acid‐sensitive functional groups. A cobalt‐catalyzed hydrogen‐atom transfer process designed by Gaspar and Carreira closed this gap with the exception of styrene derivatives.^[9]^ Snyder and co‐workers recently introduced novel HCl addition reagents,^[10]^ and Paquin and co‐workers just described the use of MsOH/CaCl_2_ for the addition of HCl to unactivated alkenes^[11]^ but neither procedure can be applied to monosubstituted alkenes.^[12]^ Herein, we disclose a decarbonylative transfer hydrochlorination of a broad range of alkenes, and the new method can also be applied to aryl‐substituted alkynes. With a low‐molecular‐weight arene as the only waste and the quench of the catalyst B(C_6_F_5_)_3_ during the hydrolytic work‐up, product purification is simple, often rendering column chromatography on silica gel, especially relevant for tertiary alkyl chlorides prone to β‐elimination, unnecessary.

The preparation of cyclohexa‐2,5‐dien‐1‐ylcarbonyl chloride derivatives **2** was accomplished in two straightforward steps (Scheme 2). The parent compound **2 aa** was obtained by Birch reduction of benzoic acid (**4 a**→**5 aa**) followed by conversion of the carboxylic acid into the acyl chloride using oxalyl chloride (**5 aa**→**2 aa**). Surrogates with R=R′=H can engage in competing transfer hydrogenation.^[13]^ For that reason, R=Me was installed by Birch alkylation with methyl iodide as the alkylating reagent (**4 a**→**5 ab** and **4 b**→**5 bb**); 4‐toluic acid (**4 b**) brought along R′=Me. Chlorination furnished both methylated surrogates **2 ab** and **2 bb** in good yields;^[14]^ the fact that **2 bb** is formed as a mixture of diastereomers is not relevant for the transfer process (see the Supporting Information for details). The acyl chlorides **2** are not extremely sensitive to moisture but were kept under an inert atmosphere for long‐term storage.

**Scheme 2:**
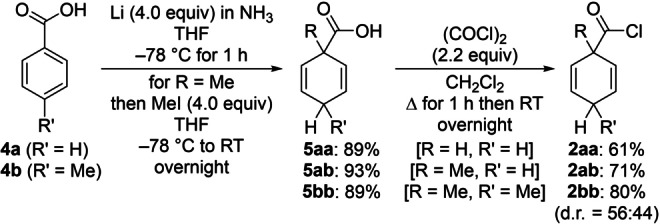
Two‐step preparation of the HCl surrogates.

We began our investigation with testing these HCl surrogates **2** in reactions with methallylbenzene (**1 a**) in the presence of 5.0 mol % of B(C_6_F_5_)_3_ as catalyst in C_6_D_6_ at room temperature (Table 1). Reactions were routinely performed in a glovebox but results were the same when conducted in a Schlenk tube in a fume cupboard. Surrogate **2 aa** furnished the desired product only in a small quantity whereas surrogates **2 ab** and **2 ac** both afforded the transfer hydrochlorination product **3 a** (entries 1–3). It is worth mentioning that B(C_6_F_5_)_3_ degraded all three surrogates completely in the absence of the alkene. We continued with **2 ab** and examined other boron Lewis acids. No reaction was seen when using BEt_3_, presumably because of its weak Lewis acidity (entry 4). BCl_3_ behaved equally well as B(C_6_F_5_)_3_ but, considering that it may also be an additional chloride source, we chose B(C_6_F_5_)_3_ as the catalyst for this transformation (entry 5). The reaction was further optimized by checking the effect of the solvent (entries 6–9). The reaction times were generally shorter in polar arene solvents and CH_2_Cl_2_; quantitative yield was obtained in CH_2_Cl_2_ within 3 h (entry 9). A lower catalyst loading led to a prolonged reaction time (entry 10).

**Table 1:** Selected examples of the optimization of the boron Lewis acid‐catalyzed transfer hydrochlorination of alkenes.^[a]^

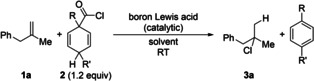					
Entry	Catalyst [mol %]	HCl surrogate	Solvent	*t* [h]	Yield [%]^[b]^
1	B(C_6_F_5_)_3_ (5.0)	**2 aa**	C_6_D_6_	20	6
2	B(C_6_F_5_)_3_ (5.0)	**2 ab**	C_6_D_6_	20	quant.
3	B(C_6_F_5_)_3_ (5.0)	**2 bb**	C_6_D_6_	20	77
4	BEt_3_^[c]^ (5.0)	**2 ab**	C_6_D_6_	20	no reaction
5	BCl_3_^[d]^ (5.0)	**2 ab**	C_6_D_6_	20	quant.
6	B(C_6_F_5_)_3_ (5.0)	**2 ab**	toluene	20	96
7	B(C_6_F_5_)_3_ (5.0)	**2 ab**	C_6_H_5_Cl	4	quant.
8	B(C_6_F_5_)_3_ (5.0)	**2 ab**	1,2‐C_6_H_4_F_2_	4	78
9^[e]^	B(C_6_F_5_)_3_ (5.0)	**2 ab**	CH_2_Cl_2_	3	quant. (78)^[f]^
10	B(C_6_F_5_)_3_ (2.5)	**2 ab**	CH_2_Cl_2_	20	91

[a] All reactions were performed on a 0.10‐mmol scale with 1.2 equiv of the HCl source **2** in 0.2 mL (0.5 M) of the indicated solvent at room temperature. [b] Yield of the volatile product determined by ^1^H NMR spectroscopy by the addition of CH_2_Br_2_ as an internal standard. [c] 1.0 M in hexane. [d] 1.0 M in toluene. [e] 0.30‐mmol scale. [f] Isolated yield in parentheses.

A wide range of alkene substrates were subjected to the optimized reaction conditions (Schemes 3 and 4). We started with studying the electronic and steric effects of substituents on the aryl ring in methallylbenzene derivatives **1 b**–**l**. Both electron‐donating and ‐withdrawing groups as well as halogen atoms were tolerated, and the corresponding products **3 b**–**l** were obtained in excellent yields throughout. Of note, Lewis basic sites as in **1 c** and **1 g** and a trifluoromethyl group as in **1 f** were compatible with B(C_6_F_5_)_3_ under these mild reaction conditions. The phenyl group can also be replaced by an α‐naphthyl and a thien‐2‐yl group as in **1 m** and **1 n**, respectively. Other 1,1‐disubstituted alkenes **1 o**–**q** as well as methylenecyclooctane (**1 r**) also reacted in near‐quantitative yields. It should be noted that B(C_6_F_5_)_3_ and BCl_3_ perform equally well but BCl_3_ was used for the transfer hydrochlorination of **1 l** and **1 r** because of purification problems. Notably, 1,1‐disubstituted alkenes **1 s**–**u** bearing a protected primary hydroxy group in the homoallylic position also reacted in high yields. A benzyl ether as in **1 t** and an ester as in **1 u** (cf. **1 g**) are particularly delicate Lewis basic groups in B(C_6_F_5_)_3_ catalysis. Our method can be extended to the trisubstituted alkenes **1 v**–**x**, α‐olefins **1 y**–**a′**, and the stryrene derivative **1 b′**, again demonstrating the tolerance of Lewis basic sites (as in **1 x**). The corresponding products formed in good yields throughout. The oligomerization of 3‐chlorostyrene (**1 b′**) prevailed under the standard reaction conditions but this was overcome by using 10 mol % of BCl_3_ instead of 5.0 mol % of B(C_6_F_5_)_3_.

**Scheme 3:**
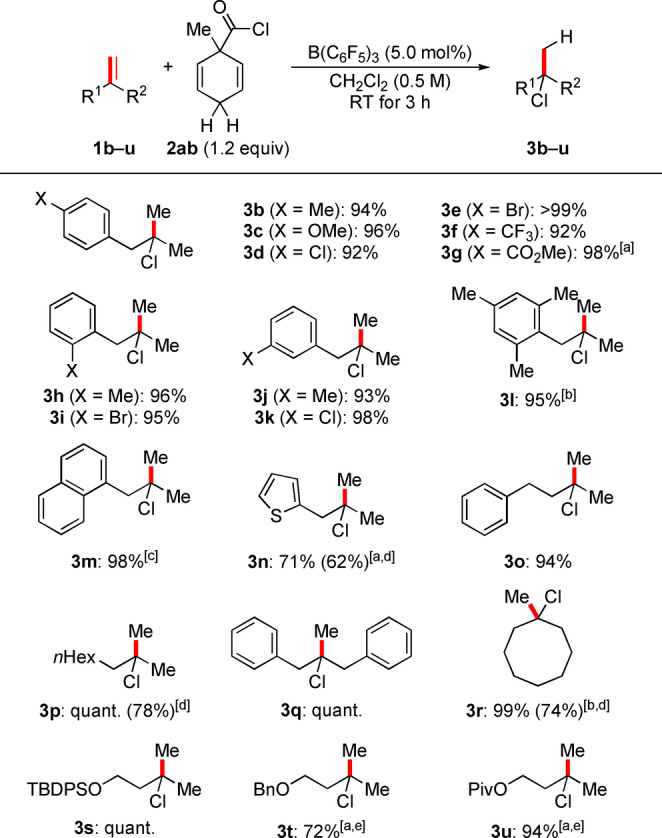
Scope I: B(C_6_F_5_)_3_‐catalyzed transfer hydrochlorination of 1,1‐disubstituted alkenes. [a] 24 h reaction time. [b] BCl_3_ instead of B(C_6_F_5_)_3_. [c] 93 % were obtained on a 2.0‐mmol scale. [d] Yield of volatile products determined by ^1^H NMR spectroscopy by the addition of CH_2_Br_2_ as an internal standard; isolated yields in parentheses. [e] 10 mol % B(C_6_F_5_)_3_ used. TBDPS=*tert*‐butyldiphenylsilyl, Bn=benzyl, Piv=pivaloyl.

**Scheme 4:**
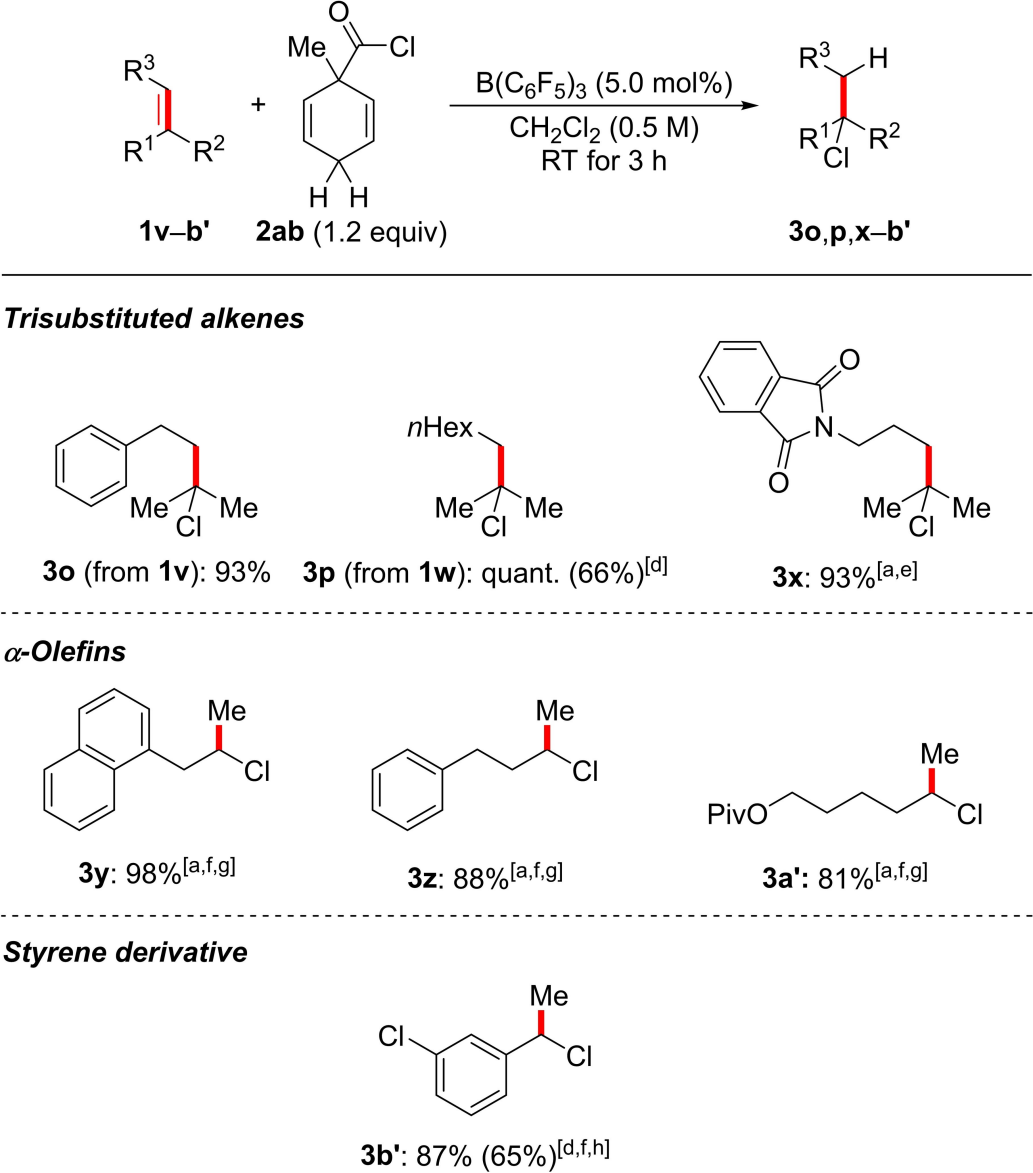
Scope II: B(C_6_F_5_)_3_‐catalyzed transfer hydrochlorination of trisubstituted alkenes, α‐olefins, and a styrene derivative. For footnotes [a]–[e], see Scheme 3. [f] 3.0 equiv of surrogate **2 ab** used. [g] 20 mol % B(C_6_F_5_)_3_ used. [h] Reaction performed with 10 mol % of BCl_3_ in C_6_D_6_ with a reaction time of 24 h.

We also probed the applicability of the decarbonylative transfer hydrochlorination for a small subset of internal alkynes (Scheme 5).^[15]^ The alkynes **6 a**–**d** bearing at least one aryl group converted cleanly into the alkenyl chlorides **7 a**–**d**. Compared to a recently reported protocol employing a strained, bicyclic HCl surrogate at 140 °C (see Scheme 1, top right),^[5]^ this is a major improvement. However, the substrate scope does neither include terminal nor dialkyl‐substituted internal alkynes.

**Scheme 5:**
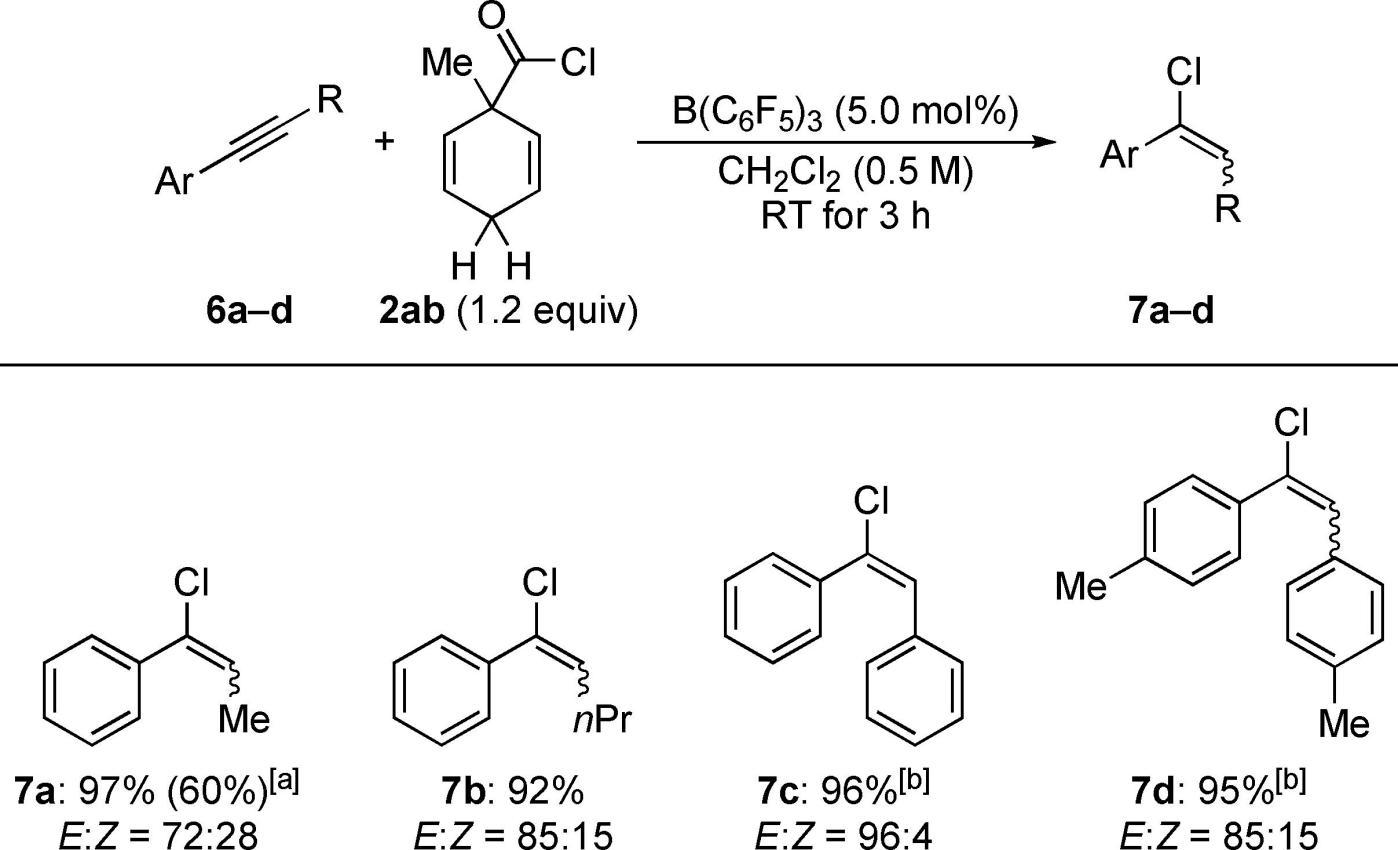
Scope III: B(C_6_F_5_)_3_‐catalyzed transfer hydrochlorination of alkynes. [a] Yield of volatile products determined by ^1^H NMR spectroscopy by the addition of CH_2_Br_2_ as an internal standard; isolated yield in parentheses. [b] Reaction performed with 10 mol % of B(C_6_F_5_)_3_ and 2.0 equiv of surrogate **2 ab** with a reaction time of 24 h.

To summarize, we developed a transition‐metal‐free transfer hydrochlorination of C−C multiple bonds that is based on a B(C_6_F_5_)_3_‐initiated Grob fragmentation of cyclohexa‐2,5‐dien‐1‐ylcarbonyl chloride derivatives.^[16]^ This new in situ release of HCl was inspired by work of Vorndran and Linker (see Scheme 1, middle).^[6]^ The required HCl surrogates are easily available from benzoic acid derivatives and are bench stable. HCl is transferred stepwise to the C−C double or triple bond with Markovnikov selectivity forming toluene or xylene and carbon monoxide as byproducts. The method is quite general as both terminal and internal alkenes undergo the hydrochlorination in high yields.

## Conflict of interest

The authors declare no conflict of interest.

## Supporting information

As a service to our authors and readers, this journal provides supporting information supplied by the authors. Such materials are peer reviewed and may be re‐organized for online delivery, but are not copy‐edited or typeset. Technical support issues arising from supporting information (other than missing files) should be addressed to the authors.

Supporting Information

## Data Availability

The data that support the findings of this study are available from the corresponding author upon reasonable request.
