# Supplementary material for: Decarbonylative Transfer Hydrochlorination of Alkenes and Alkynes Based on a B(C6F5)3‐Initiated Grob Fragmentation
Source: Angew Chem Int Ed Engl. 2022 Apr 11;61(24):e202203692. doi: 10.1002/anie.202203692 (PMC9321001; doi:10.1002/anie.202203692)
Supplement: Supplementary file 1 — Supporting Information [file ANIE-61-0-s001.pdf]

Angewandte Chemie
Eine Zeitschrift der Gesellschaft Deutscher Chemiker

Supporting Information

Decarbonylative Transfer Hydrochlorination of Alkenes and Alkynes Based on a $B(C_6F_5)_3$-Initiated Grob Fragmentation

*K. Xie, M. Oestreich**

Supporting Information

Table of Contents

1 General Information S3

2 Preparation of Surrogates S5

2.1 Synthesis of Cyclohexa-2,5-diene-1-carbonyl Chloride (**2aa**) S5

2.2 Synthesis of 1-Methylcyclohexa-2,5-diene-1-carbonyl Chloride (**2ab**) S6

2.3 Synthesis of 1,4-Dimethylcyclohexa-2,5-diene-1-carbonyl Chloride (**2ac**) S8

3 Preparation of Substrates…………………………………………….....………..…… S10

4 Decarbonylative Transfer Hydrochlorination... ………………………………… S12

4.1 General Procedure for the Decarbonylative Transfer Hydrochlorination……... ... S12

4.2 Characterization Data of Alkyl Chlorides **3a–z and 3a'**,**b'** as well as Alkenyl Chlorides **7a–d** S14

5 Scale-Up Experiment S25

6 NMR Spectra... ………………………………………………………………………... S26

7 References..... ……………………………………………………………………… S95

1 General Information

Reactions

All reactions were performed in an *MBraun* glovebox or using conventional Schlenk techniques under a static pressure of argon (glovebox) or nitrogen. Glassware for reactions performed outside the glovebox was dried under vacuum using a heat gun. Glassware for reactions performed inside the glovebox was dried overnight in a 120 °C oven and plastic syringes were dried overnight in a 60 °C oven before being transferred into the glovebox. Liquids and solutions were transferred with either syringes or glass pipettes.

Reagents and Solvents

Standard reagents and solvents were purchased from *ABCR*, *Acros*, *Alfa Aesar*, *Merck*, *Sigma-Aldrich* or *Tokyo Chemical Industry (TCI)*. Technical grade solvents for chromatography and extraction were distilled prior to use. Solvents for reactions: CH_2Cl_2, 1,2-$F_2C_6H_4$ and chlorobenzene were dried over CaH_2 , toluene was dried over sodium/benzophenone, distilled, degassed by three freeze-pump-thaw cycles and stored in a glovebox over thermally activated 4 Å molecular sieves; C_6D_6 (purchased from *Eurisotop*) was degassed by three freeze-pump thaw cycles and stored in a glovebox over thermally activated 4 Å molecular sieves; THF were dried over sodium/benzophenone and freshly distilled prior to use. $B(C_6F_5)_3$ was purchased from *Fluoropharm* company and sublimed before use. BCl_3 (1.0M solution in toluene), BEt_3(1.0M solution in hexane) were purchased from *Sigma-Aldrich* and used as received.

Chromatography

Analytical thin layer chromatography (TLC) was performed on *Alugram*® Xtra SIL G/UV_{254} silica gel 60 pre-coated aluminium-backed plates. Flash column chromatography was performed on *Grace* 60 (40–63 µm, 230–400 mesh, ASTM) silica gel using the indicated solvents.

Nuclear Magnetic Resonance (NMR) Spectroscopy

^{1}H, ^{13}C, and ^{19}F NMR spectra were recorded in $CDCl_3$ or C_6D_6 on a Bruker AV400, AV500 or AV 700 instruments. Chemical shifts are reported in parts per million (ppm) and are referenced to the residual solvent resonance as the internal standard ($CHCl_3$: δ = 7.26 ppm for ^{1}H NMR

and $CDCl_3$: δ = 77.16 ppm for ^{13}C NMR; C_6D_5H: δ = 7.16 ppm for ^{1}H NMR and C_6D_6: δ = 128.06 ppm for ^{13}C NMR). ^{19}F chemical shifts are referenced in compliance with the unified scale as recommended by the IUPAC stating the chemical shift relative to CCl_3F.[S1] Data are reported as follows: chemical shift, multiplicity (br = broad signal, s = singlet, d = doublet, t = triplet, q = quartet, m = multiplet), coupling constants (Hz), and integration.

Infrared Spectroscopy

Infrared (IR) spectra were recorded on an Agilent Technologies Cary 630 FT-IR spectrometer equipped with an ATR unit or a Jasco FT/IR-4100 spectrometer and selected absorption maxima are reported in wavenumbers (cm^{-1}).

Mass Spectrometry

High resolution mass spectrometry (HRMS) analysis was performed by the Analytical Facility at the *Institut für Chemie, Technische Universität Berlin*.

2 Preparation of Surrogates

2.1 Synthesis of cyclohexa-2,5-diene-1-carbonyl Chloride (2aa)

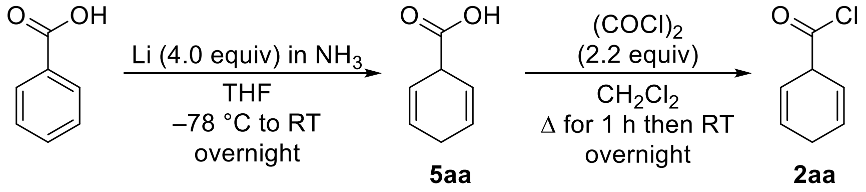

Scheme S1. Synthesis of surrogate **2aa**.

2.1.1 Cyclohexa-2,5-diene-1-carboxylic Acid (5aa)

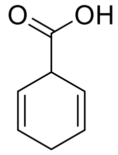

5aa
$C_7H_8O_2$
M = 124.14 g/mol

A 500-mL three-neck round bottom flask equipped with a dry ice condenser and a mechanical stirrer was flushed with N_2 for 10 min before being placed in a dry ice/acetone bath (–78 °C). Ammonia (approx. 200 mL) was condensed, and a solution of benzoic acid (10.0 g, 81.9 mmol, 1.0 equiv) in THF (50 mL) was then added dropwise. Then, lithium (2.27 g, 328 mmol, 4.0 equiv) was added portionwise until the deep dark blue of the solution persisted. The mixture was stirred at –78 °C for 1 h, removed from the cold bath and allowed to stir at room temperature overnight for the evaporation of ammonia. Water (100 mL) was added, and the aqueous phase was acidified with HCl (37%, aq.) under ice bath until pH = 2 was achieved. The mixture was extracted with Et_2O (3 × 150 mL), and the combined organic layers were dried over anhydrous $MgSO_4$, filtered and concentrated under reduced pressure. The residue was purified by flash column chromatography on silica gel with Et_2O/*n*-pentane = 1:10 as the eluent to afford **5aa** as a colorless oil (9.05 g, 89%). The NMR spectroscopic data are in accordance with those reported in the literature.[S2]

2.1.2 Cyclohexa-2,5-diene-1-carbonyl Chloride (2aa)

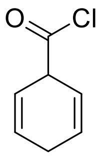

2aa
C_7H_7ClO
M = 142.58 g/mol

To a solution of cyclohexa-2,5-diene-1-carboxylic acid (**5aa**, 5.70 g, 45.9 mmol, 1.0 equiv) in dry CH_2Cl_2 (90 mL) was added oxalyl chloride (8.5 mL, 101 mmol, 2.2 equiv) dropwise under nitrogen atmosphere. The solution was heated at reflux for 1 h, cooled to room temperature and stirred overnight. The solvent and the excess of oxalyl chloride were removed under vacuum, and the crude product was purified by vacuum distillation (34 °C head, $5.6x10^{-1}$ mbar) at 50 °C oil-bath temperature to afford **2aa** as a colorless liquid (3.99 g, 61%).

^{1}H NMR (400 MHz, $CDCl_3$): δ = 6.06–6.00 (m, 2H), 5.88–5.83 (m, 2H), 4.18–4.11 (m, 1H), 2.77–2.71 (m, 2H) ppm. **^{13}C NMR** (101 MHz, $CDCl_3$): δ = 173.8, 129.0, 120.3, 53.1, 26.2 ppm. **HRMS** (APCI) calculated for $C_7H_8ClO^+$ $[M+H]^+$: 143.0259; found: 143.0258. **IR** (ATR): $\tilde{\nu}$ = 3042, 2877, 2817, 1784, 1419, 1012, 923, 784, 719 cm^{-1}.

2.2 Synthesis of 1-methylcyclohexa-2,5-diene-1-carbonyl Chloride (2ab)

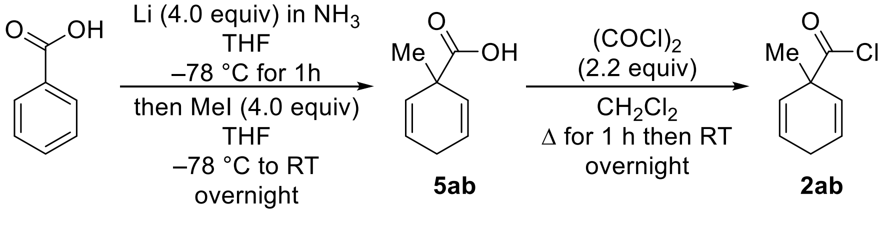

Scheme S2. Synthesis of surrogate **2ab**.

2.2.1 1-Methylcyclohexa-2,5-diene-1-carboxylic Acid (5ab)

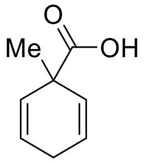

5ab
$C_8H_{10}O_2$
M = 138.17 g/mol

A 500-mL three-neck round bottom flask equipped with a dry ice condenser and a mechanical stirrer was flushed with N_2 for 10 min before being placed in a dry ice/acetone bath (–78 °C). Ammonia (approx. 200 mL) was condensed, and a solution of benzoic acid (10.0 g, 81.9 mmol, 1.0 equiv) in THF (50 mL) was then added dropwise. Then, lithium (2.27 g, 328 mmol, 4.0 equiv) was added portionwise until the deep dark blue of the solution persisted. After the mixture was stirred at –78 °C for 1 h, iodomethane (20.4 mL, 328 mmol, 4.0 equiv) was added dropwise. After addition, the mixture was removed from the cold bath and allowed to stir at room temperature overnight for the evaporation of ammonia. Water (100 mL) was added, and the aqueous phase was acidified with HCl (37%, aq.) under ice bath until pH = 2 was achieved. The mixture was extracted with Et_2O (3 × 150 mL) and the combined organic layers were dried over anhydrous $MgSO_4$, filtered and concentrated under reduced pressure. The residue was purified by flash column chromatography on silica gel with Et_2O/*n*-pentane = 1:5 as the eluent to afford **5ab** as an off-white solid (10.52 g, 93%). The NMR spectroscopic data are in accordance with those reported in the literature.[S2]

2.2.2 1-Methylcyclohexa-2,5-diene-1-carbonyl Chloride (2ab)

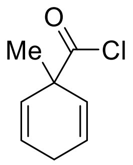

2ab
C_8H_9ClO
M = 156.61 g/mol

Acyl chloride **2ab** was synthesized according to a literature procedure[S3] with some modifications. To a solution of 1-methylcyclohexa-2,5-diene-1-carboxylic acid (**5ab**, 8.00 g, 57.9 mmol, 1.0 equiv) in dry CH_2Cl_2 (120 mL) was added oxalyl chloride (10.8 mL, 127.4 mmol, 2.2 equiv) dropwise under a nitrogen atmosphere. The solution was heated at reflux for 1 h, cooled to room temperature and stirred overnight. The solvent and the excess of oxalyl chloride were removed under vacuum, and the crude product was purified by vacuum distillation (37 °C head, 5.6×10^{-1} mbar) at 55 °C oil-bath temperature to afford **2ab** as a colorless liquid (6.45 g, 71%).

^{1}H NMR (400 MHz, $CDCl_3$): δ = 6.00–5.95 (m, 2H), 5.74–5.69 (m, 2H), 2.75–2.71 (m, 2H), 1.44 (s, 3H) ppm. **^{13}C NMR** (101 MHz, $CDCl_3$): δ = 177.3, 127.3, 126.9, 54.2, 26.9, 26.2 ppm. **HRMS**

(APCI) calculated for $C_8H_{10}ClO^+$ $[M+H]^+$: 157.0415; found: 157.0414. **IR** (ATR): $\tilde{\nu}$ = 3034, 2981, 2874, 1780, 1452, 1417, 1012, 934, 902, 781, 708 cm^{-1}.

2.3 Synthesis of 1-methylcyclohexa-2,5-diene-1-carbonyl Chloride (2ac)

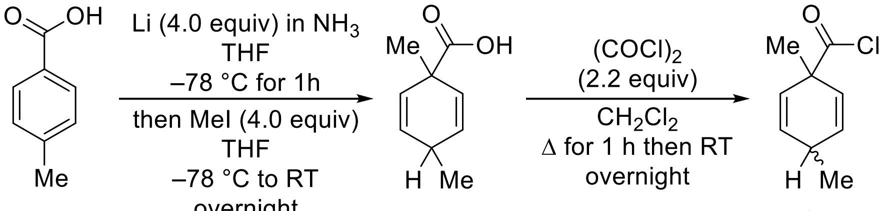

Scheme S3. Synthesis of surrogate **2ac**.

2.3.1 1,4-Dimethylcyclohexa-2,5-diene-1-carboxylic Acid (5ac)

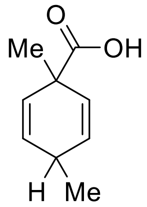

5ac
$C_9H_{12}O_2$
M = 152.19 g/mol

A 500-mL three-neck round bottom flask equipped with a dry ice condenser and a mechanical stirrer was flushed with N_2 for 10 min before being placed in a dry ice/acetone bath (–78 °C). Ammonia (approx. 200 mL) was condensed, and a solution of 4-methylbenzoic acid (10.9 g, 80.0 mmol, 1.0 equiv) in THF (50 mL) was then added dropwise. Then, lithium (2.27 g, 328 mmol, 4.0 equiv) was added portionwise until the deep dark blue of the solution persisted. After the mixture was stirred at –78 °C for 1 h, iodomethane (19.9 mL, 320 mmol, 4.0 equiv) was added dropwise. After addition, the mixture was removed from the cold bath and allowed to stir at room temperature overnight for the evaporation of ammonia. Water (100 mL) was added, and the aqueous phase was acidified with HCl (37%, aq.) under ice bath until pH = 2 was achieved. The mixture was extracted with Et_2O (3 × 150 mL) and the combined organic layers were dried over anhydrous $MgSO_4$, filtered and concentrated under reduced pressure. The residue was purified by flash column chromatography on silica gel with Et_2O/*n*-pentane = 1:6 as the eluent to afford **5ac** as a yellow oil (10.82 g, 89%). The NMR spectroscopic data are in

accordance with those reported in the literature.[S4]

2.3.2 1,4-Dimethylcyclohexa-2,5-diene-1-carbonyl Chloride (2ac)

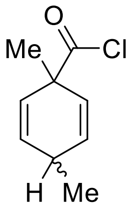

2ac
$C_9H_{11}ClO$
M = 170.64 g/mol

To a solution of 1,4-dimethylcyclohexa-2,5-diene-1-carboxylic acid (**5ac**, 3.50 g, 23.0 mmol, 1.00 equiv) in dry CH_2Cl_2 (45 mL) was added oxalyl chloride (4.3 mL, 50.6 mmol, 2.2 equiv) dropwise under a nitrogen atmosphere. The solution was heated at reflux for 1 h, cooled to room temperature and stirred overnight. The solvent and the excess of oxalyl chloride were removed under vacuum, and the crude product was purified by vacuum distillation (42 °C head, $5.6x10^{-1}$ mbar) at 60 °C oil-bath temperature to afford **2ac** as a colorless liquid (3.13 g, 80%, d.r. = 56:44).

^{1}H NMR (500 MHz, C_6D_6): δ = 5.53–5.42 (m, 4H), 2.45–2.38 (m, 0.56H, major diastereomer), 2.35–2.28 (m, 0.44H, minor diastereomer), 1.17 (s, 1.31H, minor diastereomer), 1.16 (s, 1.66H, major diastereomer), 0.85 (d, *J* = 7.3 Hz, 1.38H, minor diastereomer), 0.75 (d, *J* = 7.4 Hz, 1.71H, major diastereomer) ppm. **^{13}C NMR** (101 MHz, C_6D_6, both diastereomers): δ = 176.5, 176.4, 133.4, 133.0, 125.9, 125.8, 54.5, 54.4, 30.8, 30.6, 26.7, 26.6, 21.0, 20.4 ppm. **HRMS** (APCI) calculated for $C_9H_{12}ClO^+$ $[M+H]^+$: 171.0572; found: 171.0570. **IR** (ATR): $\tilde{\nu}$ = 3028, 2966, 2930, 2875, 1783, 1452, 1371, 940, 902, 788, 734 cm^{-1}.

3 Preparation of Substrates

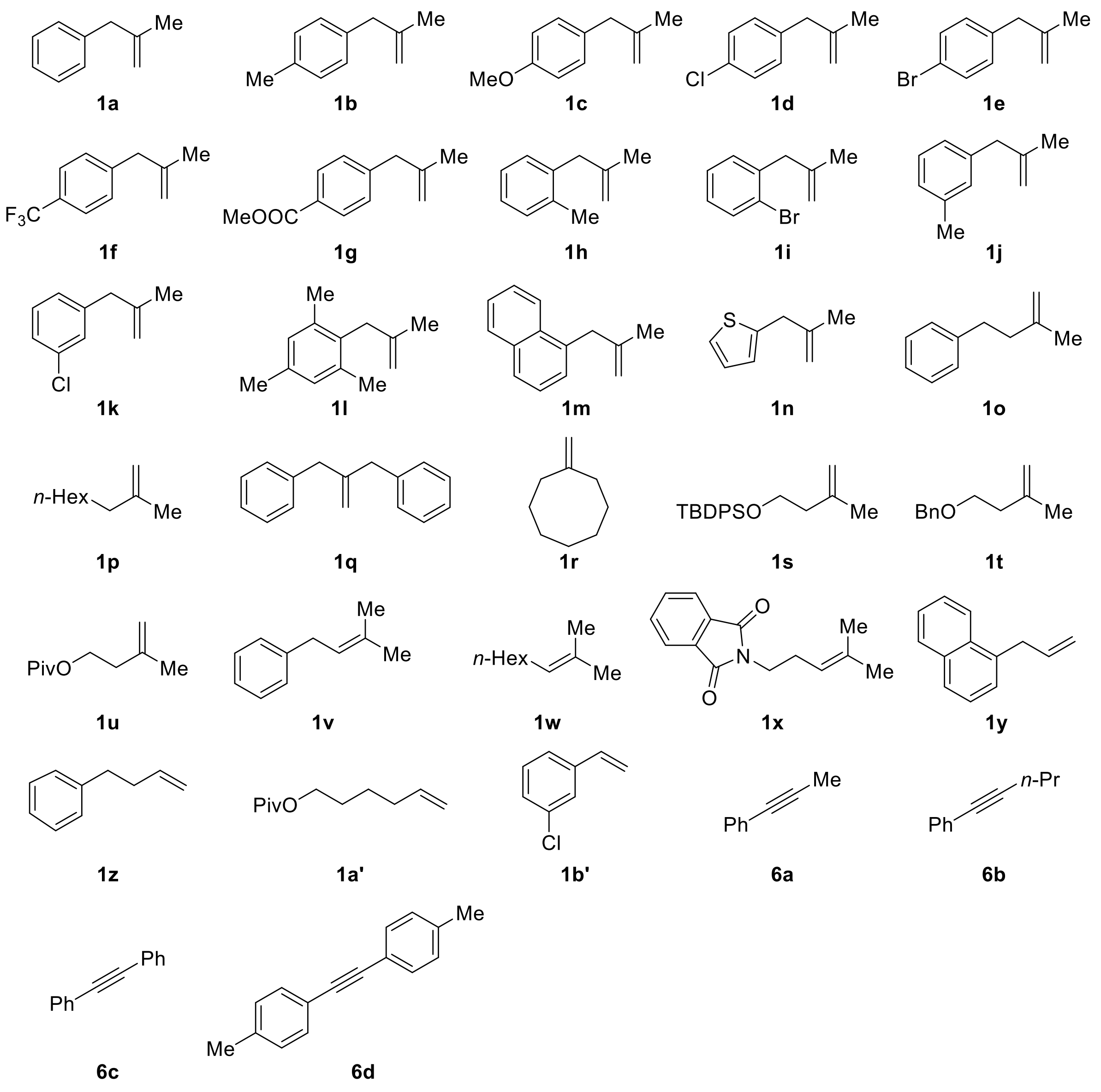

Figure S1. The collection of substrates.

3.1 General Procedure for the Synthesis of 1b–n and 1p

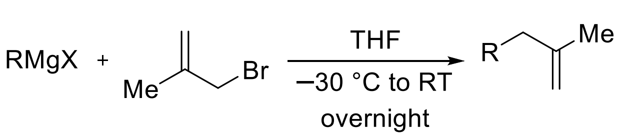

Scheme S4. Synthesis of alkene substrates.

According to a modified literature procedure,[S5] a dried Schlenk flask was charged with a solution of the indicated Grignard reagent (10.0 mmol, 1.0 equiv) in anhydrous THF (0.2M). The solution is cooled to –30 °C, and 3-bromo-2-methylprop-1-ene (12.0 mmol, 1.2 equiv) was

added dropwise under nitrogen atmosphere. The reaction mixture is stirred at –30 °C for 1 h, then slowly warmed to room temperature and stirred overnight. After addition of saturated aqueous NH_4Cl (15 mL), the mixture was extracted with *tert*-butyl methyl ether (3 × 20 mL). The combined organic phase was washed with brine, dried over Na_2SO_4 and concentrated under reduced pressure. The crude product was purified by flash column chromatography on silica gel to give the desired product.

Alkenes **1b**,[S6] **1c**,[S5] **1d**,[S5] **1e**,[S7] **1f**,[S5] **1g**,[S8] **1h**,[S5] **1i**,[S7] **1j**,[S7] **1l–n**,[S5] and **1p**[S9] were synthesized according to the general procedure and spectroscopic data were consistent with those reported.

1-Chloro-3-(2-methylallyl)benzene (**1k**) was prepared from 3-chlorophenylmagnesium bromide (10.0 mmol, 1.0 equiv) and 3-bromo-2-methylprop-1-ene (1.62 g, 12.0 mmol, 1.2 equiv) according to the general procedure. The title compound **1k** was obtained as a colorless oil (1.09 g, 65%). R_f = 0.71 (*n*-pentane). **^{1}H NMR** (400 MHz, $CDCl_3$): δ = 7.24–7.17 (m, 3H), 7.09–7.06 (m, 1H), 4.85–4.84 (m, 1H), 4,76–4.75 (m, 1H), 3.30 (s, 2H), 1.68 (s, 3H) ppm. **^{13}C NMR** (101 MHz, $CDCl_3$): δ = 144.4, 141.9, 134.2, 129.6, 129.1, 127.2, 126.4, 112.7, 44.4, 22.1 ppm. **HRMS** (APCI) calculated for $C_{10}H_{12}Cl^+$ $[M+H]^+$: 167.0622; found: 167.0619. **IR** (ATR): $\tilde{\nu}$ = 3075, 2971, 2908, 1650, 1595, 1572, 1472, 1430, 1374, 1077, 890, 773, 726, 684 cm^{-1}.

Alkenes **1o**,[S10] **1q**,[S6] **1r**,[S11] **1s**,[S12] **1t**,[S13] **1u**,[S14] **1v**,[S15] **1w**,[S16] **1x**,[S17] **1a'**,[S18] and alkyne **6d**[S19] were synthesized according to reported procedures. Alkenes **1y–z**,**b'** and alkynes **6a–c** were purchased from commercial suppliers and used as received.

4 Decarbonylative Transfer Hydrochlorination

4.1 General Procedure for the Decarbonylative Transfer Hydrochlorination

Method A (1.2 equiv of **2ab** and 5.0 mol% of $B(C_6F_5)_3$)

In a glovebox, a 1.5-mL GLC vial equipped with a magnetic stir bar was charged with the indicated alkene or alkyne (1.0 equiv) and surrogate **2ab** (1.2 equiv). CH_2Cl_2 (0.5 M) was added followed by the addition of $B(C_6F_5)_3$ (5.0 mol%). The vial was capped, and the solution was stirred in the glovebox at room temperature for 3 h or 24 h (for **1g**, **1n**, **1t**, **1u**, and **1x**). The reaction was then removed from the glovebox, filtered through a small column (covered with 2.0 cm silica gel) eluting with *n*-pentane or *n*-pentane/Et_2O = 5:1 (for **3c**, **3g**, **3s**, **3t**, **3u**, and **3x**), and all volatiles were removed under reduced pressure to obtain the analytically pure alkyl or alkenyl chloride. If necessary, the crude product is purified by flash column chromatography on silica gel.

Method B (1.2 equiv of **2ab** and 5.0 mol% of BCl_3)

For the substrates where the corresponding products are unstable towards silica gel.

In a glovebox, a 1.5-mL GLC vial equipped with a magnetic stir bar was charged with the indicated alkene (1.0 equiv) and surrogate **2ab** (1.2 equiv). CH_2Cl_2 (0.5 M) was added followed by the addition of BCl_3 (5.0 mol%, 1.0 M in toluene). The vial was capped, and the solution was stirred in the glovebox at room temperature for 3 h. The reaction was then removed from the glovebox, methanol (0.5 mL/mmol) was added to the mixture, and the mixture was stirred for 5 min. All volatiles were removed under reduced pressure to afford the analytically pure alkyl chloride.

Method C (3.0 equiv of **2ab** and 20 mol% $B(C_6F_5)_3$)

In a glovebox, a 1.5-mL GLC vial equipped with a magnetic stir bar was charged with the indicated alkene (0.20 mmol, 1.0 equiv) and surrogate **2ab** (3.0 equiv). CH_2Cl_2 (0.4 mL) was added followed by the addition of $B(C_6F_5)_3$ (20 mol%). The vial was capped, and the solution was stirred in the glovebox at room temperature for 24 h. The reaction was then removed from the glovebox, filtered through a small column (covered with 2.0 cm silica gel) eluting with *n*-

pentane or *n*-pentane/Et_2O = 5:1 (for **3a'**), and all volatiles were removed under reduced pressure to obtain the analytically pure chloroalkane. If necessary, the crude product is purified by flash column chromatography on silica gel.

Method D (3.0 equiv of **2ab** and 10 mol% of BCl_3)

In a glovebox, a 1.5-mL GLC vial equipped with a magnetic stir bar was charged with **1b'** (28 mg, 0.20 mmol, 1.0 equiv) and surrogate **2ab** (94 mg, 0.60 mmol, 3.0 equiv). C_6D_6 (0.4 mL) was added followed by the addition of BCl_3 (20 µL, 20 µmol, 10 mol%, 1.0 M in toluene). The vial was capped, and the solution was stirred in the glovebox at room temperature for 24 h. The reaction was then removed from the glovebox, methanol (0.1 mL) was added to the mixture, and the mixture was stirred for 5 min. All volatiles were removed under reduced pressure to afford the analytically pure alkyl chloride **3b'**.

Method E (2.0 equiv of **2ab** and 10 mol% of $B(C_6F_5)_3$)

In a glovebox, a 1.5-mL GLC vial equipped with a magnetic stir bar was charged with the indicated alkyne (1.0 equiv) and surrogate **2ab** (2.0 equiv). CH_2Cl_2 (0.5 M) was added followed by the addition of $B(C_6F_5)_3$ (10 mol%). The vial was capped, and the solution was stirred in the glovebox at room temperature for 24 h. The reaction was then removed from the glovebox, filtered through a small column (covered with 2.0 cm silica gel) eluting with *n*-pentane, and all volatiles were removed under reduced pressure to obtain the analytically pure alkenyl chloride.

4.2 Characterization Data of Alkyl Chlorides 3a–z and 3a',b' as well as Alkenyl Chlorides 7a–d

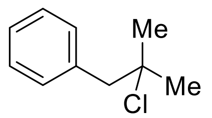

3a
$C_{10}H_{13}Cl$
M = 168.66 g/mol

(2-Chloro-2-methylpropyl)benzene (3a). Prepared from (2-methylallyl)benzene (**1a**, 39.7 mg, 0.30 mmol, 1.0 equiv), **2ab** (56.4 mg, 0.36 mmol, 1.2 equiv), and $B(C_6F_5)_3$ (7.7 mg, 15 µmol, 5.0 mol%) according to **Method A**. **3a** was obtained as a colorless oil (39.6 mg, 78%). **^{1}H NMR** (400 MHz, $CDCl_3$): δ = 7.33–7.24 (m, 5H), 3.07 (s, 2H), 1.57 (s, 6H) ppm. **^{13}C NMR** (101 MHz, $CDCl_3$): δ = 137.1, 130.9, 128.1, 127.0, 70.2, 52.0, 32.3 ppm. **HRMS** (APCI) calculated for $C_{10}H_{13}^+$ $[M\text{-}Cl]^+$: 133.1012; found: 133.1012. **IR** (ATR): $\tilde{\nu}$ = 2972, 2926, 1494, 1452, 1385, 1368, 1206, 1103, 741, 700 cm^{-1}. The spectroscopic data are in accordance with those reported.[S20]

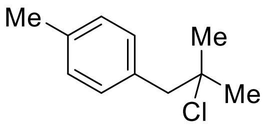

3b
$C_{11}H_{15}Cl$
M = 182.69 g/mol

1-(2-Chloro-2-methylpropyl)-4-methylbenzene (3b). Prepared from 1-methyl-4-(2-methylallyl)benzene (**1b**, 29.2 mg, 0.20 mmol, 1.0 equiv), **2ab** (37.6 mg, 0.24 mmol, 1.2 equiv), and $B(C_6F_5)_3$ (5.1 mg, 10 µmol, 5.0 mol%) according to **Method A**. **3b** was obtained as a colorless oil (34.4 mg, 94%). **^{1}H NMR** (400 MHz, $CDCl_3$): δ = 7.18–7.13 (m, 4H), 3.06 (s, 2H), 2.36 (s, 3H), 1.59 (s, 6H) ppm. **^{13}C NMR** (101 MHz, $CDCl_3$): δ = 136.5, 134.0, 130.8, 128.8, 70.4, 51.6, 32.2, 21.2 ppm. **HRMS** (APCI) calculated for $C_{11}H_{15}Cl^+$ $[M]^+$: 182.0857; found: 182.0858. **IR** (ATR): $\tilde{\nu}$ = 2972, 2924, 1513, 1453, 1384, 1368, 1206, 1110, 839, 821, 794, 756 cm^{-1}.

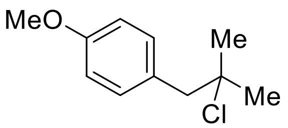

3c
$C_{11}H_{15}ClO$
M = 198.69 g/mol

1-(2-Chloro-2-methylpropyl)-4-methoxybenzene (3c). Prepared from 1-methoxy-4-(2-methylallyl)benzene (**1c**, 32.4 mg, 0.20 mmol, 1.0 equiv), **2ab** (37.6 mg, 0.24 mmol, 1.2 equiv), and $B(C_6F_5)_3$ (5.1 mg, 10 µmol, 5.0 mol%) according to **Method A**. **3c** was obtained as a colorless oil (38.3 mg, 96%). **^{1}H NMR** (400 MHz, $CDCl_3$): δ = 7.20–7.16 (m, 2H), 6.87–6.84 (m, 2H), 3.81 (s, 3H), 3.02 (s, 2H), 1.56 (s, 6H) ppm. **^{13}C NMR** (101 MHz, $CDCl_3$): δ = 158.7, 131.9, 129.2, 113.5, 70.6, 55.3, 51.1, 32.2 ppm. **HRMS** (LIFDI) calculated for $C_{11}H_{15}ClO^+$ $[M]^+$: 198.0806; found: 198.0808. **IR** (ATR): $\tilde{\nu}$ = 2966, 2927, 1610, 1510, 1459, 1368, 1301, 1246, 1177, 1111, 1035, 840, 799, 760 cm^{-1}.

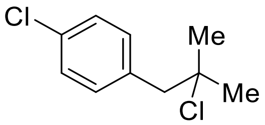

3d
$C_{10}H_{12}Cl_2$
M = 203.11 g/mol

1-Chloro-4-(2-chloro-2-methylpropyl)benzene (3d). Prepared from 1-chloro-4-(2-methylallyl)benzene (**1d**, 33.3 mg, 0.20 mmol, 1.0 equiv), **2ab** (37.6 mg, 0.24 mmol, 1.2 equiv), and $B(C_6F_5)_3$ (5.1 mg, 10 µmol, 5.0 mol%) according to **Method A**. **3d** was obtained as a colorless oil (37.5 mg, 92%). **^{1}H NMR** (400 MHz, $CDCl_3$): δ = 7.30–7.27 (m, 2H), 7.21–7.18 (m, 2H), 3.02 (s, 2H), 1.56 (s, 6H) ppm. **^{13}C NMR** (101 MHz, $CDCl_3$): δ = 135.5, 133.0, 132.2, 128.2, 69.7, 51.1, 32.3 ppm. **HRMS** (APCI) calculated for $C_{10}H_{12}Cl_2^+$ $[M]^+$: 202.0311; found: 202.0311. **IR** (ATR): $\tilde{\nu}$ = 2972, 2927, 1490, 1460, 1406, 1386, 1369, 1205, 1110, 1093, 1015, 845, 789, 720, 667 cm^{-1}.

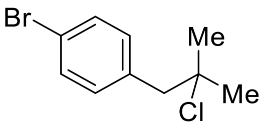

3e
$C_{10}H_{12}BrCl$
M = 247.56 g/mol

1-Bromo-4-(2-chloro-2-methylpropyl)benzene (3e). Prepared from 1-bromo-4-(2-methylallyl)benzene (**1e**, 42.2 mg, 0.20 mmol, 1.0 equiv), **2ab** (37.6 mg, 0.24 mmol, 1.2 equiv), and $B(C_6F_5)_3$ (5.1 mg, 10 µmol, 5.0 mol%) according to **Method A**. **3e** was obtained as a colorless oil (49.4 mg, >99%). **^{1}H NMR** (400 MHz, $CDCl_3$): δ = 7.46–7.42 (m, 2H), 7.16–7.12 (m, 2H), 3.01 (s, 2H), 1.56 (s, 6H) ppm. **^{13}C NMR** (101 MHz, $CDCl_3$): δ = 136.0, 132.6, 131.2, 121.1, 69.6, 51.2, 32.3 ppm. **HRMS** (APCI) calculated for $C_{10}H_{12}BrCl^+$ $[M]^+$: 245.9806; found: 245.9808. **IR** (ATR): $\tilde{\nu}$ = 2971, 2924, 1486, 1453, 1403, 1386, 1368, 1204, 1110, 1071, 1011, 842, 785, 714 cm^{-1}.

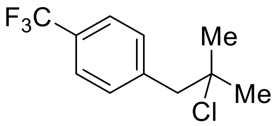

3f
$C_{11}H_{12}ClF_3$
M = 236.66 g/mol

1-(2-Chloro-2-methylpropyl)-4-(trifluoromethyl)benzene (3f). Prepared from 1-(2-methylallyl)-4-(trifluoromethyl)benzene (**1f**, 40.0 mg, 0.20 mmol, 1.0 equiv), **2ab** (37.6 mg, 0.24 mmol, 1.2 equiv), and $B(C_6F_5)_3$ (5.1 mg, 10 µmol, 5.0 mol%) according to **Method A**. **3f** was obtained as a colorless oil (43.5 mg, 92%). **^{1}H NMR** (400 MHz, $CDCl_3$): δ = 7.57 (d, *J* = 8.1 Hz, 2H), 7.39 (d, *J* = 8.1 Hz, 2H), 3.11 (s, 2H), 1.59 (s, 6H) ppm. **^{13}C NMR** (101 MHz, $CDCl_3$): δ = 141.0, 131.3, 129.4 (q, *J* = 32.5 Hz), 125.0 (q, *J* = 3.8 Hz), 124.4 (q, *J* = 272.5 Hz), 69.3, 51.5, 32.4 ppm. **^{19}F NMR** (659 MHz, $CDCl_3$) δ = -62.44 (s, 3F) ppm**.** **HRMS** (APCI) calculated for $C_{11}H_{12}F_3^+$ $[M-Cl]^+$: 201.0886; found: 201.0888. **IR** (ATR): $\tilde{\nu}$ = 2975, 2928, 1619, 1417, 1322, 1162, 1114, 1067, 1019, 854, 802 cm^{-1}.

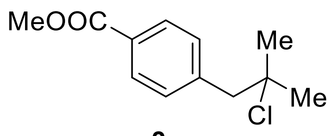

3g
$C_{12}H_{15}ClO_2$
M = 226.70 g/mol

Methyl 4-(2-chloro-2-methylpropyl)benzoate (3g). Prepared from 1 methyl 4-(2-methylallyl)benzoate (**1g**, 38.0 mg, 0.20 mmol, 1.0 equiv), **2ab** (37.6 mg, 0.24 mmol, 1.2 equiv), and $B(C_6F_5)_3$ (5.1 mg, 10 µmol, 5.0 mol%) according to **Method A**. **3g** was obtained as a colorless oil (44.5 mg, 98%). ^{1}**H NMR** (400 MHz, $CDCl_3$): δ = 8.00–7.96 (m, 2H), 7.35–7.32 (m, 2H), 3.91 (s, 3H), 3.11 (s, 2H), 1.57 (s, 6H) ppm. ^{13}**C NMR** (101 MHz, $CDCl_3$): δ = 167.1, 142.3, 131.0, 129.3, 128.9, 69.5, 52.2, 51.7, 32.4 ppm. **HRMS** (APCI) calculated for $C_{12}H_{16}ClO_2^+$ $[M+H]^+$: 227.0834; found: 227.0835. **IR** (ATR): $\tilde{\nu}$ = 2928, 1717, 1609, 1434, 1274, 1179, 1106, 1020, 864, 756, 707 cm^{-1}.

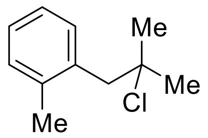

3h
$C_{11}H_{15}Cl$
M = 182.69 g/mol

1-(2-Chloro-2-methylpropyl)-2-methylbenzene (3h). Prepared from 1-methyl-2-(2-methylallyl)benzene (**1h**, 29.2 mg, 0.20 mmol, 1.0 equiv), **2ab** (37.6 mg, 0.24 mmol, 1.2 equiv), and $B(C_6F_5)_3$ (5.1 mg, 10 µmol, 5.0 mol%) according to **Method A**. **3h** was obtained as a colorless oil (35.0 mg, 96%). ^{1}**H NMR** (400 MHz, $CDCl_3$): δ = 7.28–7.25 (m, 1H), 7.20–7.13 (m, 3H), 3.16 (s, 2H), 2.39 (s, 3H), 1.63 (s, 6H) ppm. ^{13}**C NMR** (101 MHz, $CDCl_3$): δ = 137.4, 135.6, 131.9, 130.7, 127.1, 125.5, 71.4, 47.7, 32.6, 20.7 ppm. **HRMS** (APCI) calculated for $C_{11}H_{15}Cl^+$ $[M]^+$: 182.0857; found: 182.0858. **IR** (ATR): $\tilde{\nu}$ = 2971, 2926, 1493, 1457, 1383, 1368, 1117, 741 cm^{-1}.

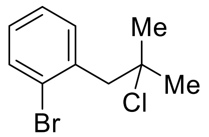

3i
$C_{10}H_{12}BrCl$
M = 247.56 g/mol

1-Bromo-2-(2-chloro-2-methylpropyl)benzene (3i). Prepared from 1-bromo-2-(2-methylallyl)benzene (**1i**, 42.2 mg, 0.20 mmol, 1.0 equiv), **2ab** (37.6 mg, 0.24 mmol, 1.2 equiv), and $B(C_6F_5)_3$ (5.1 mg, 10 µmol, 5.0 mol%) according to **Method A**. **3i** was obtained as a colorless oil (46.9 mg, 95%). ^{1}**H NMR** (400 MHz, $CDCl_3$): δ = 7.58 (dd, *J* = 8.0, 1.3 Hz, 1H), 7.50 (dd, *J* = 7.7, 1.8 Hz, 1H), 7.28 (td, *J* = 7.5, 1.3 Hz, 1H), 7.13 (td, *J* = 7.7, 1.8 Hz, 1H), 3.33 (s, 2H), 1.65 (s, 6H) ppm. ^{13}**C NMR** (101 MHz, $CDCl_3$): δ = 136.7, 133.1, 133.0, 128.7, 127.1, 126.2, 71.0, 49.8, 32.6 ppm. **HRMS** (APCI) calculated for $C_{10}H_{12}BrCl^+$ $[M]^+$: 245.9806; found: 245.9807. **IR** (ATR): $\tilde{\nu}$ = 2973, 2924, 1468, 1434, 1386, 1368, 1110, 1092, 1024, 746 cm^{-1}.

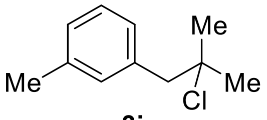

3j
$C_{11}H_{15}Cl$
M = 182.69 g/mol

1-(2-Chloro-2-methylpropyl)-3-methylbenzene (3j). Prepared from 1-methyl-3-(2-methylallyl)benzene (**1j**, 29.2 mg, 0.20 mmol, 1.0 equiv), **2ab** (37.6 mg, 0.24 mmol, 1.2 equiv), and $B(C_6F_5)_3$ (5.1 mg, 10 µmol, 5.0 mol%) according to **Method A**. **3j** was obtained as a colorless oil (34.1 mg, 93%). **^{1}H NMR** (400 MHz, $CDCl_3$): δ = 7.24–7.18 (m, 1H), 7.11–7.06 (m, 3H), 3.06 (s, 2H), 2.36 (s, 3H), 1.59 (s, 6H) ppm. **^{13}C NMR** (101 MHz, $CDCl_3$): δ = 137.6, 137.0, 131.7, 128.0, 127.9, 127.7, 70.3, 51.9, 32.3, 21.6 ppm. **HRMS** (APCI) calculated for $C_{11}H_{15}Cl^+$ $[M]^+$: 182.0857; found: 182.0859. **IR** (ATR): $\tilde{\nu}$ = 2972, 2924, 1606, 1487, 1454, 1384, 1368, 1107, 783, 742, 701 cm^{-1}.

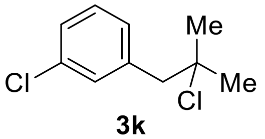

3k
$C_{10}H_{12}Cl_2$
M = 203.11 g/mol

1-Chloro-3-(2-chloro-2-methylpropyl)benzene (3k). Prepared from 1-chloro-3-(2-methylallyl)benzene (**1k**, 33.3 mg, 0.20 mmol, 1.0 equiv), **2ab** (37.6 mg, 0.24 mmol, 1.2 equiv), and $B(C_6F_5)_3$ (5.1 mg, 10 µmol, 5.0 mol%) according to **Method A**. **3k** was obtained as a colorless oil (39.8 mg, 92%). **^{1}H NMR** (400 MHz, $CDCl_3$): δ = 7.31–7.27 (m, 3H), 7.20–7.18 (m, 1H), 3.07 (s, 2H), 1.62 (s, 6H) ppm. **^{13}C NMR** (101 MHz, $CDCl_3$): δ = 139.0, 133.8, 130.9, 129.3, 129.1, 127.2, 69.5, 51.4, 32.3 ppm. **HRMS** (APCI) calculated for $C_{10}H_{12}Cl_2^+$ $[M]^+$: 202.0311; found: 202.0313. **IR** (ATR): $\tilde{\nu}$ = 2972, 2927, 1596, 1572, 1473, 1428, 1386, 1369, 1205, 1107, 1089, 776, 706, 683 cm^{-1}.

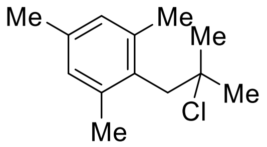

3l
$C_{13}H_{19}Cl$
M = 210.75 g/mol

2-(2-Chloro-2-methylpropyl)-1,3,5-trimethylbenzene (3l). Prepared from 1,3,5-trimethyl-2-(2-methylallyl)benzene (**1l**, 34.9 mg, 0.20 mmol, 1.0 equiv), **2ab** (37.6 mg, 0.24 mmol, 1.2 equiv), and BCl_3 (10 µL, 10 µmol, 5.0 mol%, 1.0 M in toluene) according to **Method B**. **3l** was obtained as a colorless oil (40.2 mg, 95%). **^{1}H NMR** (400 MHz, $CDCl_3$):δ = 6.89 (s, 2H), 3.28 (s, 2H), 2.39 (s, 6H), 2.28 (s,3H), 1.65 (s, 6H) ppm. **^{13}C NMR** (101 MHz, $CDCl_3$): δ = 138.1, 136.0, 131.7, 129.5, 73.1, 43.5, 33.5, 21.8, 20.9 ppm. **HRMS** (APCI) calculated for $C_{13}H_{19}Cl^+$ $[M]^+$: 210.1170; found: 210.1174. **IR** (ATR): $\tilde{\nu}$ = 2970, 2921, 1611, 1455, 1381, 1368, 1108, 850 cm^{-1}.

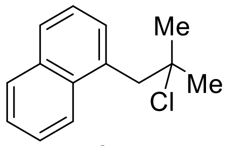

3m
$C_{14}H_{15}Cl$
M = 218.72 g/mol

1-(2-Chloro-2-methylpropyl)naphthalene (3m). Prepared from 1-(2-methylallyl)naphthalene (**1m**, 36.5 mg, 0.20 mmol, 1.0 equiv), **2ab** (37.6 mg, 0.24 mmol, 1.2 equiv), and $B(C_6F_5)_3$ (5.1 mg, 10 µmol, 5.0 mol%) according to **Method A**. **3m** was obtained as a colorless oil (42.9 mg, 98%). **^{1}H NMR** (400 MHz, $CDCl_3$): δ = 8.14 (d, *J* = 8.4 Hz, 1H), 7.87–7.85 (m, 1H), 7.81–7.78 (m, 1H), 7.54–7.43 (m, 4H), 3.63 (s, 2H), 1.64 (s, 6H) ppm. **^{13}C NMR** (101 MHz, $CDCl_3$): δ = 134.1, 133.5, 133.0, 129.8, 128.9, 127.9, 125.9, 125.5, 125.2, 124.9, 71.3, 47.0, 33.0 ppm. **HRMS** (APCI) calculated for $C_{14}H_{15}Cl^+$ $[M]^+$: 218.0857; found: 218.0860. **IR** (ATR): $\tilde{\nu}$ = 2972, 2927, 1594, 1509, 1450, 1386, 1368, 1104, 775 cm^{-1}.

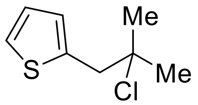

3n
$C_8H_{11}ClS$
M = 174.69 g/mol

2-(2-Chloro-2-methylpropyl)thiophene (3n). Prepared from 2-(2-methylallyl)thiophene (**1n**, 41.5 mg, 0.30 mmol, 1.0 equiv), **2ab** (56.4 mg, 0.36 mmol, 1.2 equiv), and $B(C_6F_5)_3$ (7.7 mg, 15 µmol, 5.0 mol%) according to **Method A**. **3n** was obtained as a colorless oil (32.7 mg, 62%). **^{1}H NMR** (500 MHz, $CDCl_3$): δ = 7.21 (dd, *J* = 5.1, 1.2 Hz, 1H), 6.98 (dd, *J* = 5.1, 3.5 Hz, 1H), 6.93–6.92 (m, 1H), 3.30 (s, 2H), 1.62 (s, 6H) ppm. **^{13}C NMR** (125 MHz, $CDCl_3$): δ = 138.7, 127.9, 126.7, 124.8, 69.5, 46.2, 32.1 ppm. **HRMS** (LIFDI) calculated for $C_8H_{11}ClS^+$ $[M]^+$: 174.0265; found: 174.0269. **IR** (ATR): $\tilde{\nu}$ = 2959, 2923, 1807, 1453, 1385, 1369, 1111, 1018, 694 cm^{-1}.

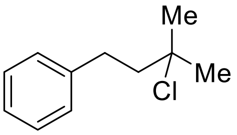

3o
$C_{11}H_{15}Cl$
M = 182.69 g/mol

(3-chloro-3-methylbutyl)benzene (3o). Prepared from (3-methylbut-3-en-1-yl)benzene (**1o**, 29.2 mg, 0.20 mmol, 1.0 equiv) or (3-methylbut-2-en-1-yl)benzene (**1s**, 29.2 mg, 0.20 mmol, 1.0 equiv), **2ab** (37.6 mg, 0.24 mmol, 1.2 equiv), and $B(C_6F_5)_3$ (5.1 mg, 10 µmol, 5.0 mol%) according to **Method A**. **3o** was obtained as a colorless oil (from **1o**: 34.2 mg, 94%; from **1v**: 33.8 mg, 93%). **^{1}H NMR** (400 MHz, $CDCl_3$): δ = 7.34–7.29 (m, 2H), 7.24–7.20 (m, 3H), 2.87–2.83 (m, 2H), 2.09–2.05 (m, 2H), 1.67 (s, 6H) ppm. **^{13}C NMR** (101 MHz, $CDCl_3$): δ = 141.9, 128.6, 128.5, 126.1, 70.7, 48.1, 32.6, 31.8 ppm. **HRMS** (APCI) calculated for $C_{11}H_{15}Cl^+$ $[M]^+$: 182.0857; found: 182.0857. **IR** (ATR): $\tilde{\nu}$ = 2972, 2926, 1496, 1453, 1369, 1111, 746, 698 cm^{-1}. The spectroscopic data are in accordance with those reported.[S21]

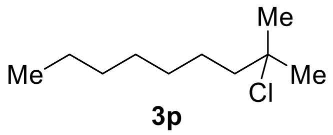

3p
$C_{10}H_{21}Cl$
M = 176.73 g/mol

2-Chloro-2-methylnonane (3p). Prepared from 2-methylnon-1-ene (**1p**, 42.1 mg, 0.30 mmol, 1.0 equiv) or 2-methylnon-2-ene (**1t**, 42.1 mg, 0.30 mmol, 1.0 equiv), **2ab** (56.4 mg, 0.36 mmol, 1.2 equiv), and $B(C_6F_5)_3$ (7.7 mg, 15 µmol, 5.0 mol%) according to **Method A**. **3p** was obtained as a colorless oil (from **1p**: 41.6 mg, 78%; from **1w**: 34.8 mg, 66%). **^{1}H NMR** (400 MHz, $CDCl_3$): δ = 1.75–1.71 (m, 2H), 1.56 (s, 6H), 1.50–1.43 (m, 2H), 1.35–1.23 (m, 8H), 0.89 (t, *J* = 7.1 Hz, 3H) ppm. **^{13}C NMR** (101 MHz, $CDCl_3$): δ = 71.5, 46.3, 32.6, 32.0, 29.8, 29.4, 25.3, 22.8, 14.2 ppm. **HRMS** (APCI) calculated for $C_{10}H_{21}^+$ $[M\text{-}Cl]^+$: 141.1638; found: 141.1637. **IR** (ATR): $\tilde{\nu}$ = 2924, 2854, 1463 cm^{-1}.

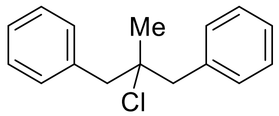

3q
$C_{16}H_{17}Cl$
M = 244.76 g/mol

(2-Chloro-2-methylpropane-1,3-diyl)dibenzene (3q). Prepared from (2-methylenepropane-1,3-diyl)dibenzene (**1q**, 41.7 mg, 0.20 mmol, 1.0 equiv), **2ab** (37.6 mg, 0.24 mmol, 1.2 equiv), and $B(C_6F_5)_3$ (5.1 mg, 10 µmol, 5.0 mol%) according to **Method A**. **3q** was obtained as a colorless oil (49.5 mg, quant.). **^{1}H NMR** (400 MHz, $CDCl_3$): δ = 7.36–7.28 (m, 10H), 3.18 (d, *J* = 13.8 Hz, 2H), 3.09 (d, *J* = 13.8 Hz, 2H), 1.46 (s, 3H) ppm. **^{13}C NMR** (101 MHz, $CDCl_3$): δ = 136.7, 131.4, 128.1, 127.1, 72.9, 50.7, 29.1 ppm. **HRMS** (APCI) calculated for $C_{16}H_{17}Cl^+$ $[M]^+$: 244.1014; found: 244.1015. **IR** (ATR): $\tilde{\nu}$ = 3027, 2921, 1492, 1451, 1377, 1082, 750, 736, 697 cm^{-1}.

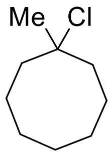

3r
$C_9H_{17}Cl$
M = 160.69 g/mol

1-Chloro-1-methylcyclooctane (3r). Prepared from methylenecyclooctane (**1r**, 37.3 mg, 0.30 mmol, 1.0 equiv), **2ab** (56.4 mg, 0.36 mmol, 1.2 equiv), and BCl_3 (15 µL, 15 µmol, 5.0 mol%, 1.0 M in toluene) according to **Method B**. **3r** was obtained as a colorless oil (35.6 mg, 74%). **^{1}H NMR** (400 MHz, $CDCl_3$): δ = 2.21–2.11 (m, 2H), 1.91–1.82 (m, 2H), 1.75–1.41 (m, 13H). **^{13}C NMR** (101 MHz, $CDCl_3$): δ = 76.8, 40.4, 32.7, 28.2, 24.9, 23.7 ppm. **HRMS** (LIFDI) calculated for $C_9H_{17}^+$ $[M\text{-}Cl]^+$: 125.1325; found: 125.1325. **IR** (ATR): $\tilde{\nu}$ = 2918, 2852, 1445, 1109 cm^{-1}.

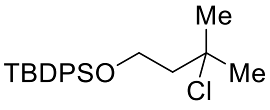

3s
$C_{21}H_{29}ClOSi$
M = 361.00 g/mol

***tert*-Butyl(3-chloro-3-methylbutoxy)diphenylsilane (3s)**. Prepared from *tert*-butyl((3-methylbut-3-en-1-yl)oxy)diphenylsilane (**1s**, 32.5 mg, 0.10 mmol, 1.0 equiv), **2ab** (18.8 mg, 0.12 mmol, 1.2 equiv), and $B(C_6F_5)_3$ (2.6 mg, 5 μmol, 5.0 mol%) according to **Method A**. **3s** was obtained as a colorless oil (36.1mg, 100%). **^{1}H NMR** (400 MHz, $CDCl_3$): δ = 7.70–7.68 (m, 4H), 7.45–7.38 (m, 6H), 3.91 (t, *J* = 6.8 Hz, 2H), 2.08 (t, *J* = 6.8 Hz, 2H), 1.60 (s, 6H), 1.07 (s, 9H) ppm. **^{13}C NMR** (101 MHz, $CDCl_3$): δ = 135.7, 133.8, 129.8, 127.8, 69.7, 61.2, 48.1, 33.2, 27.0, 19.3 ppm. **HRMS** (APCI) calculated for $C_{21}H_{30}ClOSi^+$ $[M+H]^+$: 361.1749; found: 361.1748. **IR** (ATR): $\tilde{\nu}$ = 2929, 2888, 2855, 1470, 1427, 1388, 1107, 833, 738, 701 cm^{-1}. The spectroscopic data are in accordance with those reported.[S21]

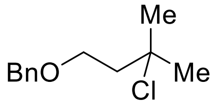

3t
$C_{12}H_{17}ClO$
M = 212.72 g/mol

((3-Chloro-3-methylbutoxy)methyl)benzene (3t). Prepared from (((3-methylbut-3-en-1-yl)oxy)methyl)benzene (**1t**, 35.3 mg, 0.20 mmol, 1.0 equiv), **2ab** (37.6 mg, 0.24 mmol, 1.2 equiv), and $B(C_6F_5)_3$ (10.2 mg, 20 μmol, 10.0 mol%) according to **Method A**. The crude product needs to be purified by flash column chromatography on silica gel with Et_2O/*n*-pentane = 1:50 as the eluent to afford **3t** as a colorless oil (30.8mg, 72%). ***R$_f$*** = 0.42 (50:1 *n*-pentane:Et_2O). **^{1}H NMR** (400 MHz, $CDCl_3$): δ = 7.38–7.27 (m, 5H), 4.52 (s, 2H), 3.72 (t, *J* = 6.7 Hz, 2H), 2.12 (t, *J* = 6.7 Hz, 2H), 1.62 (s, 6H) ppm. **^{13}C NMR** (101 MHz, $CDCl_3$): δ = 138.5, 128.5, 127.8, 127.7, 73.2, 69.6, 67.6, 45.3, 33.1 ppm. **HRMS** (APCI) calculated for $C_{12}H_{18}ClO$ $[M+H]^+$: 213.1041; found: 213.1043. **IR** (ATR): $\tilde{\nu}$ = 2970, 2925, 2859, 1453, 1368, 1113, 1028, 736, 687 cm^{-1}. The spectroscopic data are in accordance with those reported.[S22]

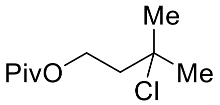

3u
$C_{10}H_{19}ClO_2$
M = 206.71 g/mol

3-Chloro-3-methylbutyl pivalate (3u). Prepared from 3-methylbut-3-en-1-yl pivalate (**1u**, 34.1 mg, 0.20 mmol, 1.0 equiv), **2ab** (37.6 mg, 0.24 mmol, 1.2 equiv), and $B(C_6F_5)_3$ (10.2 mg, 20 μmol, 10.0 mol%) according to **Method A**. **3u** was obtained as a light yellow oil (38.9 mg, 94%). **^{1}H NMR** (400 MHz, $CDCl_3$): δ = 4.29 (t, *J* = 6.8 Hz, 2H), 2.10 (t, *J* = 6.8 Hz, 2H), 1.62 (s, 6H), 1.19 (s, 9H) ppm. **^{13}C NMR** (101 MHz, $CDCl_3$): δ = 178.6, 68.7, 61.6, 44.2, 38.8, 33.0, 27.3 ppm. **HRMS** (APCI) calculated for $C_{10}H_{20}ClO_2^+$ $[M+H]^+$: 207.1147; found: 207.1146. **IR** (ATR): $\tilde{\nu}$ = 2960, 2921, 2852, 1730, 1459, 1283, 1153 cm^{-1}.

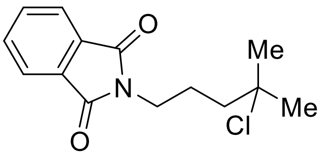

3x
$C_{14}H_{16}ClNO_2$
M = 265.74 g/mol

2-(4-Chloro-4-methylpentyl)isoindoline-1,3-dione (3x). Prepared from 2-(4-methylpent-3-en-1-yl)isoindoline-1,3-dione (**1x**, 22.7 mg, 0.10 mmol, 1.0 equiv), **2ab** (18.8 mg, 0.12 mmol, 1.2 equiv), and $B(C_6F_5)_3$ (5.1 mg, 10 µmol, 10.0 mol%) according to **Method A**. **3x** was obtained as a white solid (24.6mg, 93%). **M.P.** = 86–88 °C. **^{1}H NMR** (400 MHz, $CDCl_3$): δ = 7.86–7.81 (m, 2H), 7.73–7.68 (m, 2H), 3.71 (t, *J* = 6.9 Hz, 2H), 1.94–1.85 (m, 2H), 1.80–1.76 (m, 2H), 1.55 (s, 6H) ppm. **^{13}C NMR** (101 MHz, $CDCl_3$): δ = 168.5, 134.1, 132.2, 123.4, 70.2, 43.1, 38.0, 32.5, 24.7 ppm. **HRMS** (APCI) calculated for $C_{14}H_{17}ClNO_2^+$ $[M+H]^+$: 266.0942; found: 266.0941. **IR** (ATR): $\tilde{\nu}$ = 2971, 2930, 1772, 1707, 1466, 1437, 1395, 1360, 1083, 1030, 719 cm^{-1}. The spectroscopic data are in accordance with those reported.[S22]

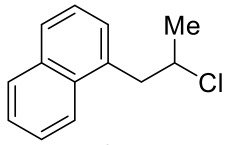

3y
$C_{13}H_{13}Cl$
M = 204.70 g/mol

1-(2-Chloropropyl)naphthalene (3y). Prepared from 1-allylnaphthalene (**1y**, 41.7 mg, 0.20 mmol, 1.0 equiv), **2ab** (94.0 mg, 0.60 mmol, 3.0 equiv), and $B(C_6F_5)_3$ (20.5 mg, 40 µmol, 20 mol%) according to **Method C**. **3y** was obtained as a colorless oil (40.1 mg, 98%). **^{1}H NMR** (400 MHz, $CDCl_3$): δ = 8.04–8.02 (m, 1H), 7.91–7.89 (m, 1H), 7.81 (d, *J* = 7.9 Hz, 1H), 7.59–7.50 (m, 2H), 7.47–7.44 (m, 1H), 7.41–7.39 (m, 1H), 4.49–4.41 (m, 1H), 3.66 (dd, *J* = 14.1, 6.5 Hz, 1H), 3.41 (dd, *J* = 14.1, 7.8 Hz, 1H), 1.57 (d, *J* = 6.5 Hz, 3H) ppm. **^{13}C NMR** (101 MHz, $CDCl_3$): δ = 134.2, 134.1, 132.0, 129.1, 128.0, 127.9, 126.3, 125.8, 125.5, 123.6, 57.7, 44.1, 25.1 ppm. **HRMS** (APCI) calculated for $C_{13}H_{13}Cl^+$ $[M]^+$: 204.0701; found: 204.0701. **IR** (ATR): $\tilde{\nu}$ = 2971, 2925, 1595, 1509, 1443, 1394, 1376, 1013, 792, 773 cm^{-1}.

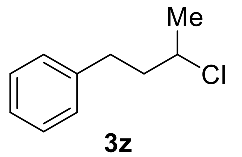

3z
$C_{10}H_{13}Cl$
M = 168.66 g/mol

(3-Chlorobutyl)benzene (3z). Prepared from but-3-en-1-ylbenzene (**1z**, 26.4 mg, 0.20 mmol, 1.0 equiv), **2ab** (94.0 mg, 0.60 mmol, 3.0 equiv), and $B(C_6F_5)_3$ (20.5 mg, 40 µmol, 20 mol%) according to **Method C**. **3z** was obtained as a colorless oil (29.6 mg, 88%). **^{1}H NMR** (400 MHz, $CDCl_3$): δ = 7.33–7.27 (m, 2H), 7.24–7.19 (m, 3H), 4.05–3.97 (m, 1H), 2.90–2.83 (m, 1H), 2.80–2.72 (m, 1H), 2.06–2.00 (m, 2H), 1.54 (d, *J* = 6.6 Hz, 3H) ppm. **^{13}C NMR** (101 MHz, $CDCl_3$): δ = 141.2, 128.6, 128.6, 126.2, 58.1, 42.0, 33.0, 25.6 ppm. **HRMS** (LIFDI) calculated for $C_{10}H_{13}Cl^+$ $[M]^+$: 168.0701; found: 168.0700. **IR** (ATR): $\tilde{\nu}$ = 2969, 2925, 1494, 1452, 746, 689 cm^{-1}. The spectroscopic data are in accordance with those reported.[S21]

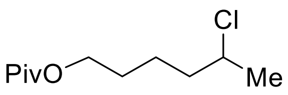

3a'
$C_{11}H_{21}ClO_2$
M = 220.74 g/mol

5-Chlorohexyl pivalate (3a'). Prepared from hex-5-en-1-yl pivalate (**1a'**, 36.9mg, 0.20 mmol, 1.0 equiv), **2ab** (94.0 mg, 0.60 mmol, 3.0 equiv), and $B(C_6F_5)_3$ (20.5 mg, 40 μmol, 20.0 mol%) according to **Method C**. The crude product needs to be purified by flash column chromatography on silica gel with Et_2O/*n*-pentane = 1:50 as the eluent to afford **3a'** as a colorless oil (35.7 mg, 81%). $\boldsymbol{R_f}$ = 0.30 (50:1 *n*-pentane:Et_2O). ^{1}**H NMR** (400 MHz, $CDCl_3$): δ = 4.10–3.98 (m, 3H), 1.79–1.44 (m, 9H), 1.20 (s, 9H) ppm. ^{13}**C NMR** (101 MHz, $CDCl_3$): δ = 178.7, 64.1, 58.6, 40.0, 38.9, 28.3, 27.3, 25.5, 23.2 ppm. **HRMS** (APCI) calculated for $C_{11}H_{22}ClO_2^+$ $[M+H]^+$: 221.1303; found: 221.1303. **IR** (ATR): $\tilde{\nu}$ = 2969, 2933, 2870, 1727, 1479, 1459, 1284, 1155 cm^{-1}.

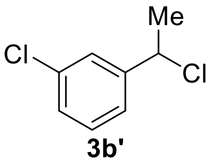

3b'
$C_8H_8Cl_2$
M = 175.05 g/mol

1-Chloro-3-(1-chloroethyl)benzene (3b'). Prepared from 1-chloro-3-vinylbenzene (**1b'**, 28 mg, 0.20 mmol, 1.0 equiv), **2ab** (94 mg, 0.60 mmol, 3.0 equiv), and BCl_3 (20 μL, 20 μmol, 10 mol%, 1.0 M in toluene) according to **Method D**. **3b'** was obtained as a colorless oil (22.9 mg, 65%). ^{1}**H NMR** (400 MHz, $CDCl_3$): δ = 7.42–7.41 (m, 1H), 7.30–7.27 (m, 3H), 5.04 (q, *J* = 6.8 Hz, 1H) 1.83 (d, *J* = 6.8 Hz, 3H) ppm. ^{13}**C NMR** (101 MHz, $CDCl_3$): δ = 144.9, 134.6, 130.1, 128.5, 126.9, 124.9, 57.8, 26.6 ppm. **HRMS** (LIFDI) calculated for $C_8H_8Cl_2^+$ $[M]^+$: 173.9998; found: 173.9997. **IR** (ATR): $\tilde{\nu}$ = 2962, 2925, 2854, 1595, 1573, 1476, 1431, 1082, 786, 696 cm^{-1}. The spectroscopic data are in accordance with those reported.[S23]

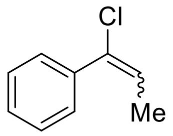

7a
C_9H_9Cl
M = 152.62 g/mol

(1-Chloroprop-1-en-1-yl)benzene (7a). Prepared from prop-1-yn-1-ylbenzene (**6a**, 34.85 mg, 0.30 mmol, 1.0 equiv), **2ab** (56.4 mg, 0.36 mmol, 1.2 equiv), and $B(C_6F_5)_3$ (7.7 mg, 15 μmol, 5.0 mol%) according to **Method A**. **7a** was obtained as a yellow oil (27.4 mg, 60%, *E*/*Z* = 72:28), the ratio of *E*/*Z* was determined by ^{1}H NMR analysis according to the reported literature.[S24] (*E*)-**7a**: ^{1}**H NMR** (400 MHz, $CDCl_3$): δ = 7.43–7.34 (m, 5H), 6.05 (q, *J* = 7.3 Hz, 1H), 1.74 (d, *J* = 7.3 Hz, 3H) ppm. ^{13}**C NMR** (101 MHz, $CDCl_3$): (*E*-**7a**) δ = 137.2, 131.0, 129.0, 128.5, 128.3, 124.7, 15.5 ppm. (*Z*)-**7a**: ^{1}**H NMR** (400 MHz, $CDCl_3$): δ = 7.58–7.55 (m, 2H), 7.34–7.30 (m, 3H), 6.21 (q, *J* = 6.7 Hz, 1H), 1.97 (d, *J* = 6.7 Hz, 3H) ppm. ^{13}**C NMR** (101 MHz, $CDCl_3$): δ = 138.6, 133.9, 129.0, 128.4, 126.4, 122.5, 15.3 ppm. **HRMS** (APCI) calculated for $C_9H_{10}Cl^+$ $[M+H]^+$: 153.0466; found: 153.0466. **IR** (ATR): $\tilde{\nu}$ =3058, 3023,

2922, 2853, 1491, 1422, 759, 699 cm^{-1}.

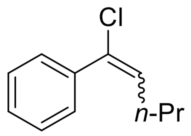

7b
$C_{11}H_{13}Cl$
M = 180.68 g/mol

(1-Chloropent-1-en-1-yl)benzene (7b). Prepared from pent-1-yn-1-ylbenzene (**6b**, 43.3 mg, 0.30 mmol, 1.0 equiv), **2ab** (56.4 mg, 0.36 mmol, 1.2 equiv), and $B(C_6F_5)_3$ (7.7 mg, 15 μmol, 5.0 mol%) according to **Method A**. **7b** was obtained as a yellow oil (49.8 mg, 92%, *E*/*Z* = 85:15), the stereochemistry of **7b** was determined by 2D-NMR analysis and the ratio of *E*/*Z* was determined by ^{1}H NMR analysis. (*E*)-**7b**: **^{1}H NMR** (400 MHz, $CDCl_3$): δ = 7.41–7.33 (m, 5H), 5.98 (t, *J* = 7.8 Hz, 1H), 2.12–2.06 (m, 2H), 1.44 (dq *J* = 7.4 Hz, 2H), 0.90 (t, *J* = 7.4 Hz, 3H) ppm. **^{13}C NMR** (101 MHz, $CDCl_3$): δ = 137.5, 130.4, 128.9, 128.5, 128.3, 126.5, 31.9, 22.9, 13.8 ppm. (*Z*)-**7b**: **^{1}H NMR** (400 MHz, $CDCl_3$): δ = 7.60–7.57 (m, 2H), 7.33–7.30 (m, 3H), 6.16 (t, *J* = 7.0 Hz, 1H), 2.42–2.36 (m, 2H), 1.55 (dq *J* = 7.4 Hz, 2H), 1.01 (t, *J* = 7.4 Hz, 3H) ppm. **^{13}C NMR** (101 MHz, $CDCl_3$): δ = 138.6, 132.9, 130.5, 128.4, 128.3, 128.1, 31.8, 22.0, 14.0 ppm. **HRMS** (APCI) calculated for $C_{11}H_{14}Cl^+$ [M+H]$^+$: 181.0779; found: 181.0777. **IR** (ATR): $\tilde{\nu}$ = 2958, 2928, 2869, 1442, 758, 695 cm^{-1}.

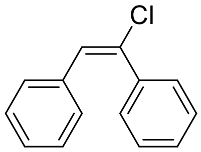

7c
$C_{14}H_{11}Cl$
M = 214.69 g/mol

(*E*)-(1-Chloroethene-1,2-diyl)dibenzene (7c). Prepared from 1,2-diphenylethyne (**6c**, 35.6 mg, 0.20 mmol, 1.0 equiv), **2ab** (62.6 mg, 0.40 mmol, 2.0 equiv), and $B(C_6F_5)_3$ (10.2 mg, 20 μmol, 10 mol%) according to **Method E**. **7c** was obtained as a colorless oil (41.4 mg, 96%, *E*/*Z* = 96:4), the ratio of *E*/*Z* was determined by ^{1}H NMR analysis according to the reported literature.[S25] **^{1}H NMR** (400 MHz, $CDCl_3$): (*E*-**7c**) δ = 7.43–7.39 (m, 2H), 7.36–7.32 (m, 3H), 7.18–7.15 (m, 3H), 7.04–7.01 (m, 2H), 6.97 (s, 1H) ppm. **^{13}C NMR** (101 MHz, $CDCl_3$): (*E*-**7c**) δ = 137.9, 135.5, 133.2, 131.7, 129.3, 129.0, 128.9, 128.7, 128.4, 127.5. **HRMS** (APCI) calculated for $C_{14}H_{12}Cl^+$ [M+H]$^+$: 215.0623; found: 215.0625. **IR** (ATR): $\tilde{\nu}$ = 1492, 1443, 932, 898, 754, 712, 688 cm^{-1}.

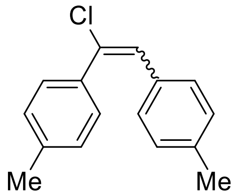

7d
$C_{16}H_{15}Cl$
M = 242.75 g/mol

4,4'-(1-Chloroethene-1,2-diyl)bis(methylbenzene) (7d). Prepared from 1,2-di-*p*-tolylethyne (**6d**, 20.6 mg, 0.1 mmol, 1.0 equiv), **2ab** (56.4 mg, 0.2 mmol, 2.0 equiv), and $B(C_6F_5)_3$ (5.1 mg, 10 μmol, 10 mol%) according to **Method E**. **7d** was obtained as a colorless oil (23.1 mg, 95%, *E*/*Z* = 85:15), the ratio of *E*/*Z* was determined by ^{1}H NMR analysis

according to the reported literature.[S26] (*E*)-**7d**: **^{1}H NMR** (500 MHz, $CDCl_3$): δ = 7.30–7.28 (m, 2H), 7.14–7.12 (m, 2H), 6.98–6.96 (m, 2H), 6.92–6.91 (m, 2H), 6.88 (s, 1H), 2.37 (s, 3H), 2.27 (s, 3H) ppm. **^{13}C NMR** (125 MHz, $CDCl_3$): (*E*-**7d**) δ = 139.0, 137.3, 135.2, 132.8, 132.5, 129.4, 129.2, 129.1, 128.8, 128.6, 21.5, 21.3 ppm. (*Z*)-**7d**: **^{1}H NMR** (500 MHz, $CDCl_3$): δ = 7.66–7.64 (m, 2H), 7.61–7.58 (m, 2H), 7.22–7.20 (m, 4H), 7.01 (s, 1H), 2.40 (s, 3H), 2.39 (s, 3H) ppm. **^{13}C NMR** (125 MHz, $CDCl_3$): δ = 138.8, 138.0, 136.8, 129.5, 129.1, 126.7, 125.3, 29.9 ppm (Due to the low concentration of the minor isomer, not all signals could be detected). **HRMS** (APCI) calculated for $C_{16}H_{16}Cl^+$ $[M+H]^+$: 243.0936; found: 243.0934. **IR** (ATR): $\tilde{\nu}$ = 2918, 1510, 913, 808, 789, 751, 713 cm^{-1}.

5 Scale-Up Experiment

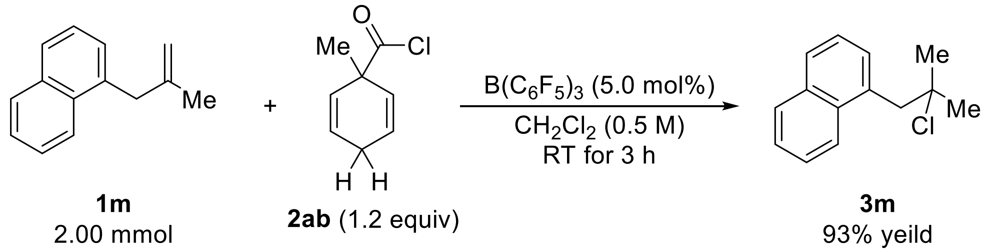

1m
2.00 mmol

2ab (1.2 equiv)

3m
93% yeild

Scheme 5. Sacle-up experiment.

In a glovebox, a 10-mL sealed tube equipped with a magnetic stir bar was charged with 1-(2-methylallyl)naphthalene (**1m**, 364 mg, 2.00 mmol, 1.0 equiv) and surrogate **2ab** (375.9 mg, 24.00 mmol, 1.2 equiv). CH_2Cl_2 (4 mL) was added followed by the addition of $B(C_6F_5)_3$ (51.2 mg, 100 µmol, 5.0 mol%). The tube was sealed and removed from the glovebox. The solution was stirred at room temperature for 3 h, and then filtered through a short column (covered with 2.0 cm silica gel) eluting with *n*-pentane, and all volatiles were removed under reduced pressure to afford **3m** as a colorless oil (408.3 mg, 93%).

6 NMR Spectra

Figure S2. Cyclohexa-2,5-diene-1-carbonyl chloride (2aa): ^{1}H NMR spectrum (400 MHz, $CDCl_3$, 298 K)

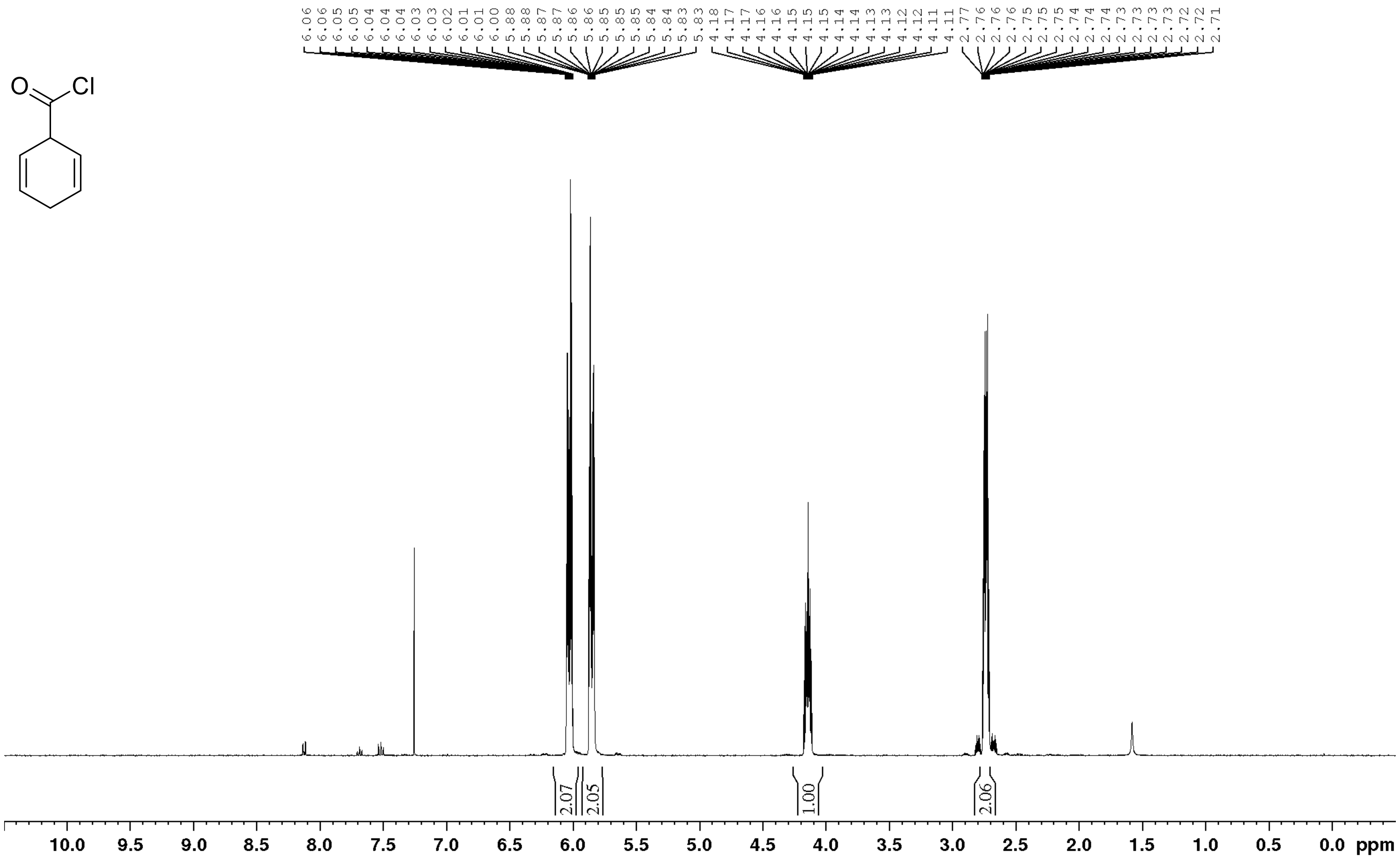

Figure S3. Cyclohexa-2,5-diene-1-carbonyl chloride (2aa): $^{13}C\{^{1}H\}$ NMR (101 MHz, $CDCl_3$, 298 K)

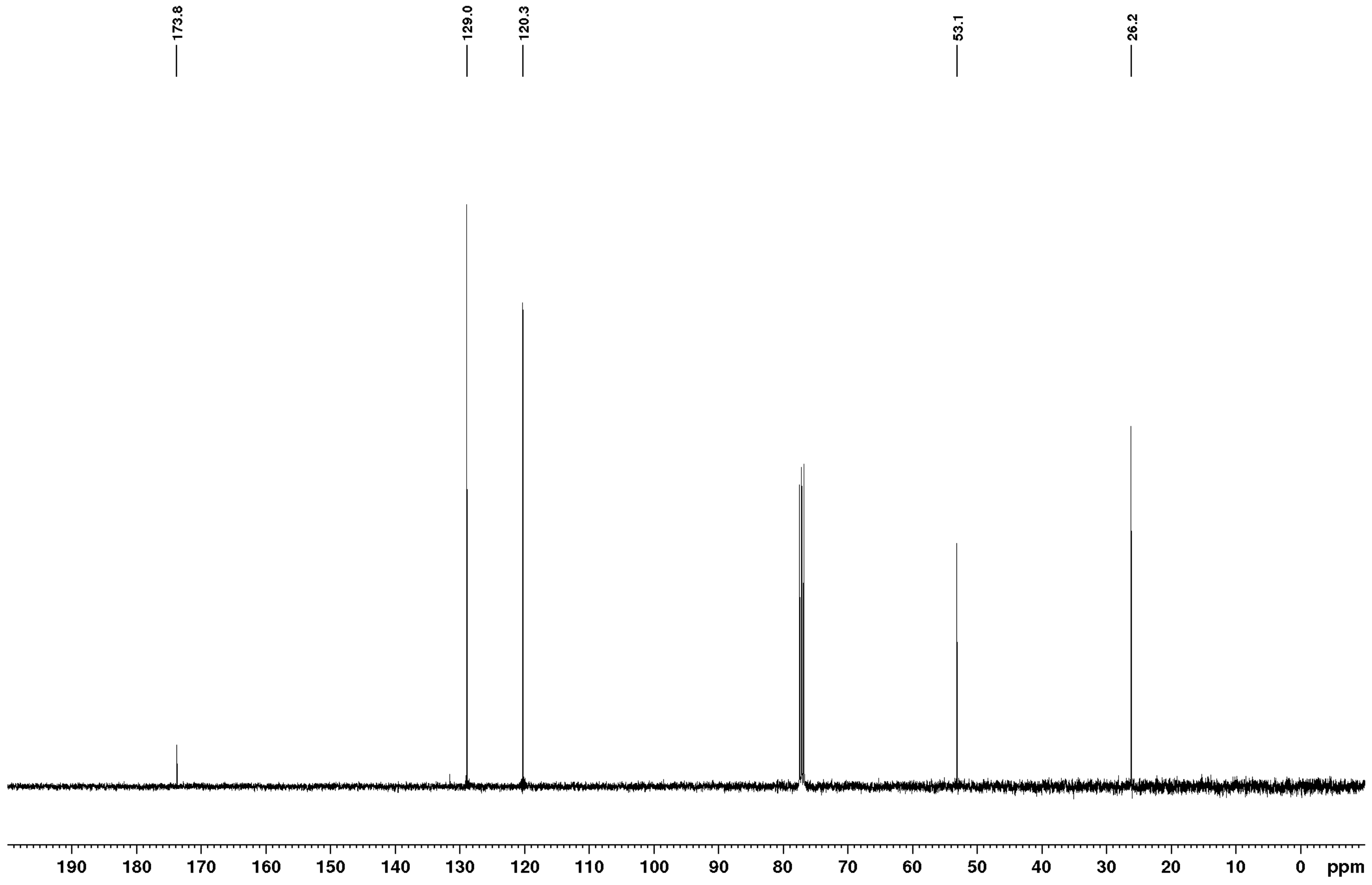

Figure S4. 1-Methylcyclohexa-2,5-diene-1-carbonyl chloride (2ab): ^{1}H NMR spectrum (400 MHz, $CDCl_3$, 298 K)

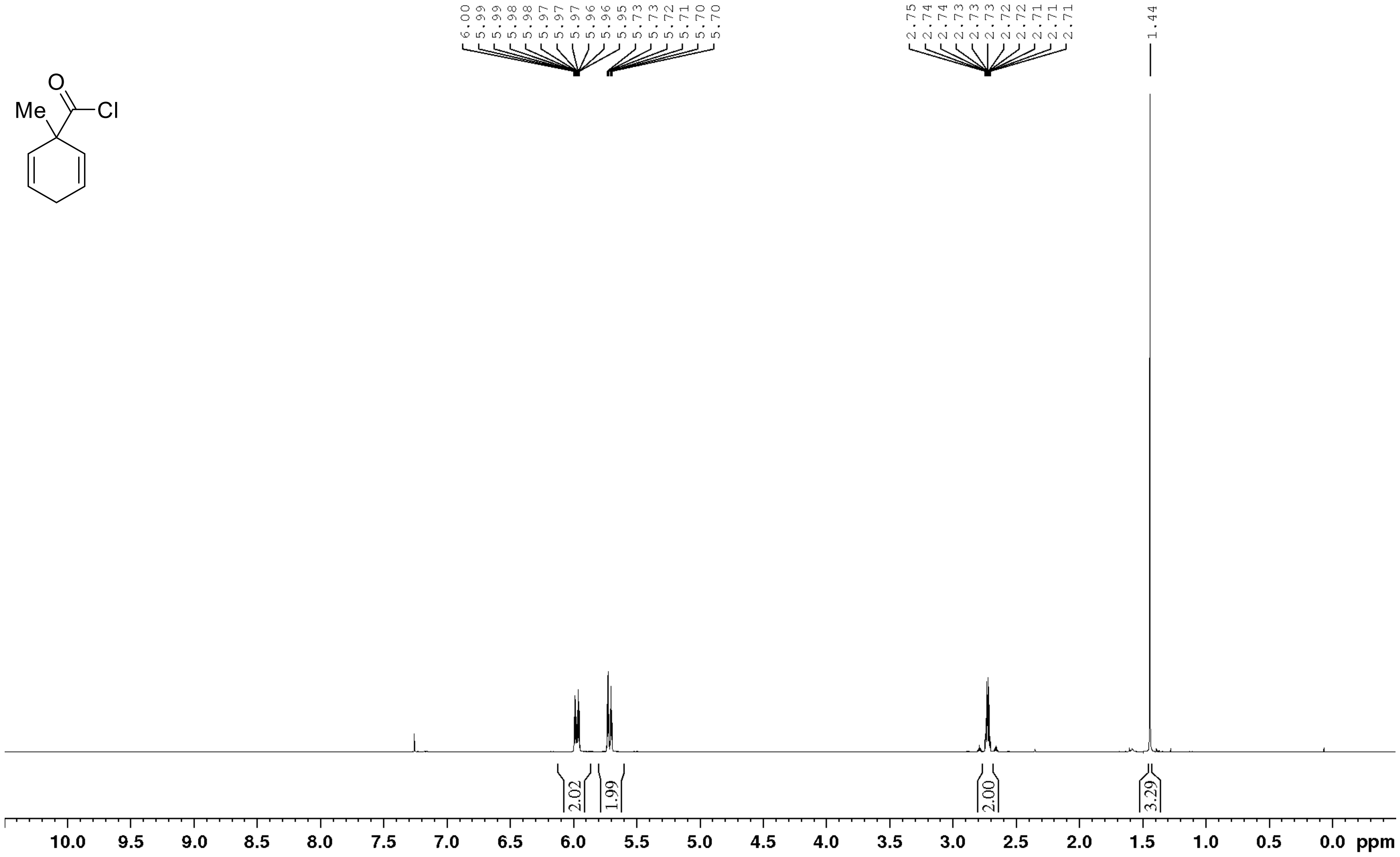

Figure S5. 1-Methylcyclohexa-2,5-diene-1-carbonyl chloride (2ab): $^{13}C\{^{1}H\}$ NMR (101 MHz, $CDCl_3$, 298 K)

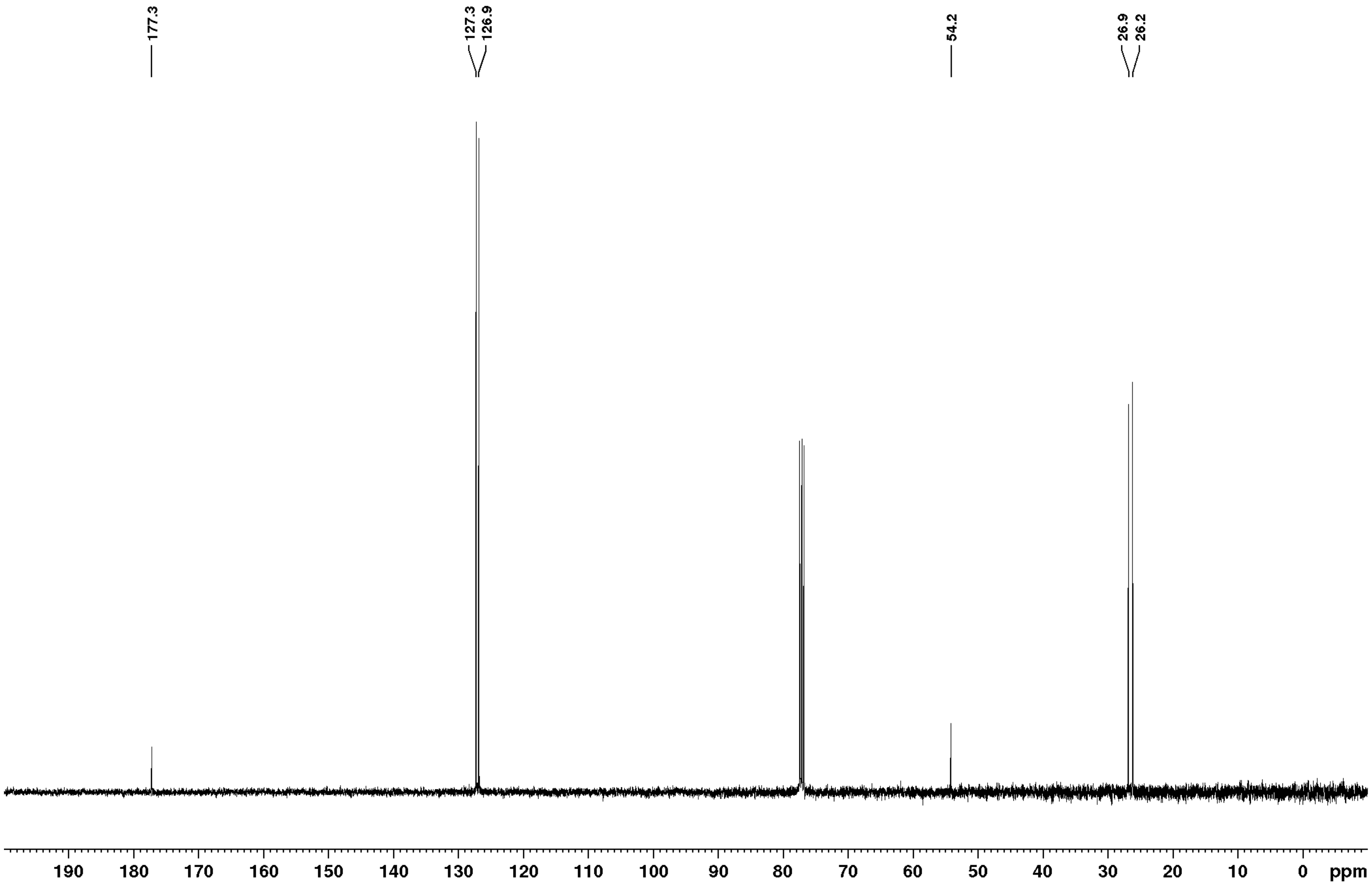

Figure S6. 1,4-Dimethylcyclohexa-2,5-diene-1-carbonyl chloride (2ac): ^{1}H NMR spectrum (500 MHz, C_6D_6, 298 K)

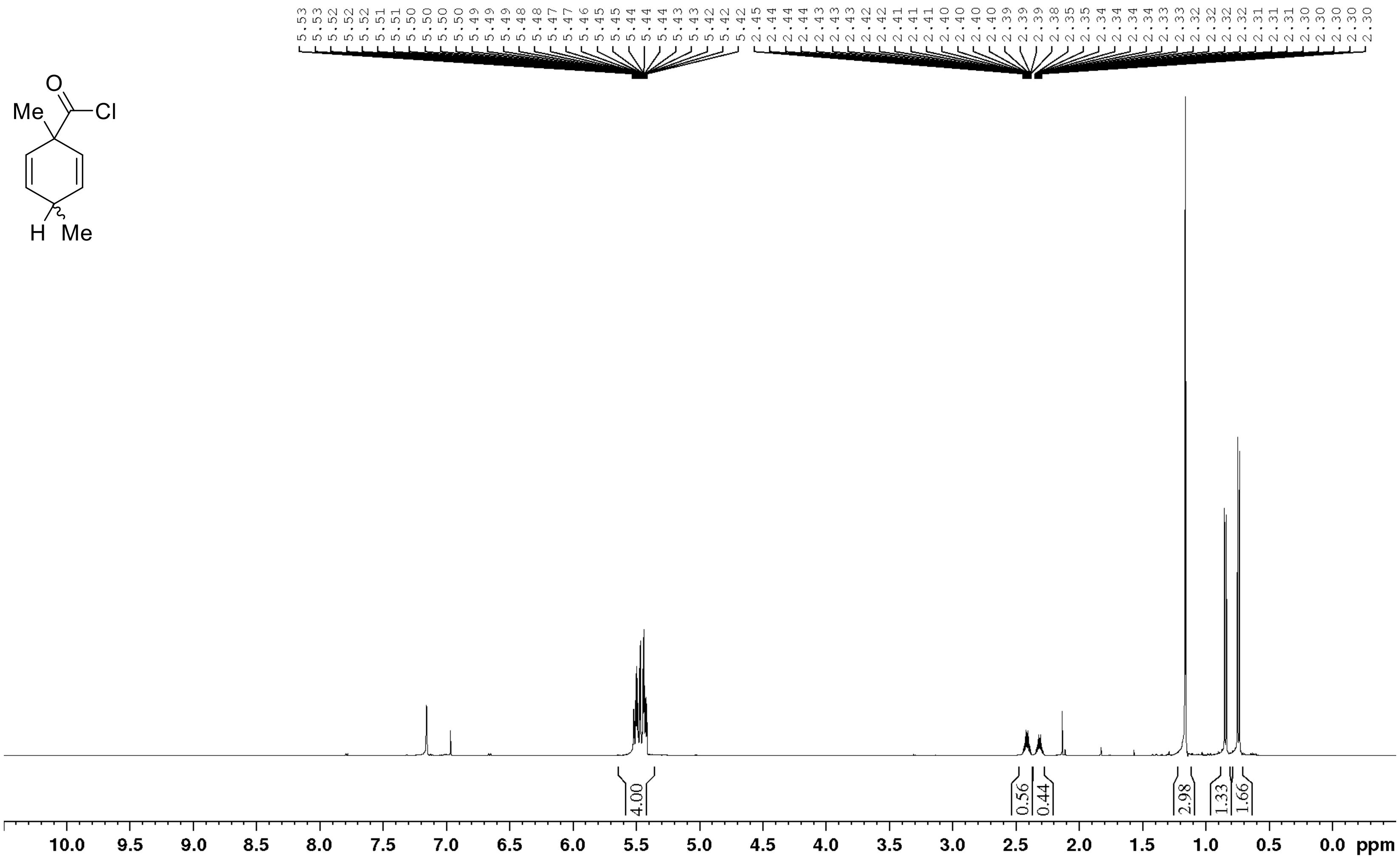

Figure S7. 1,4-Dimethylcyclohexa-2,5-diene-1-carbonyl chloride (2ac): $^{13}C\{^{1}H\}$ NMR (101 MHz, C_6D_6, 298 K)

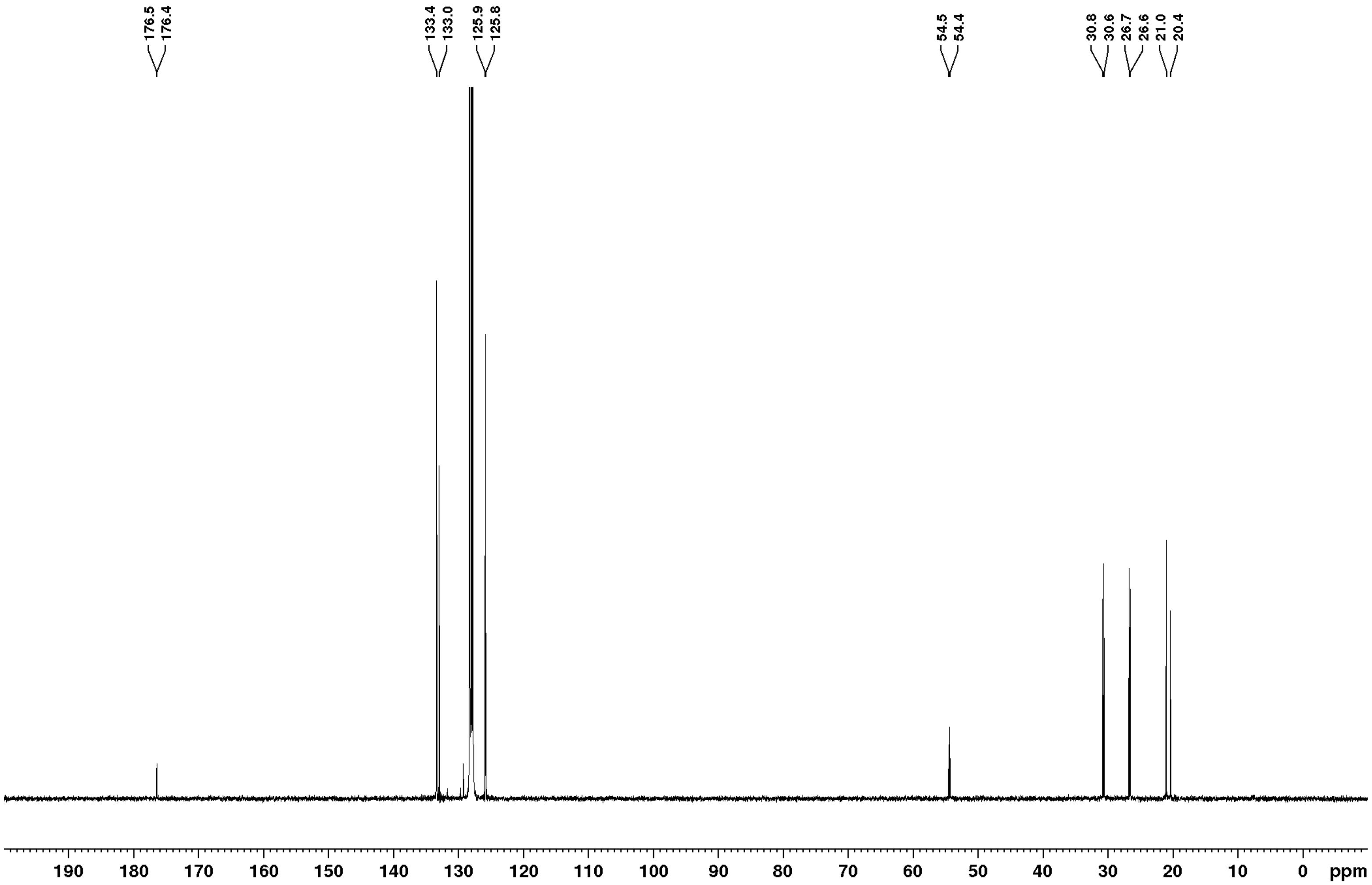

Figure S8. 1-Chloro-3-(2-methylallyl)benzene (**1k**): ^{1}H NMR spectrum (400 MHz, $CDCl_3$, 298 K)

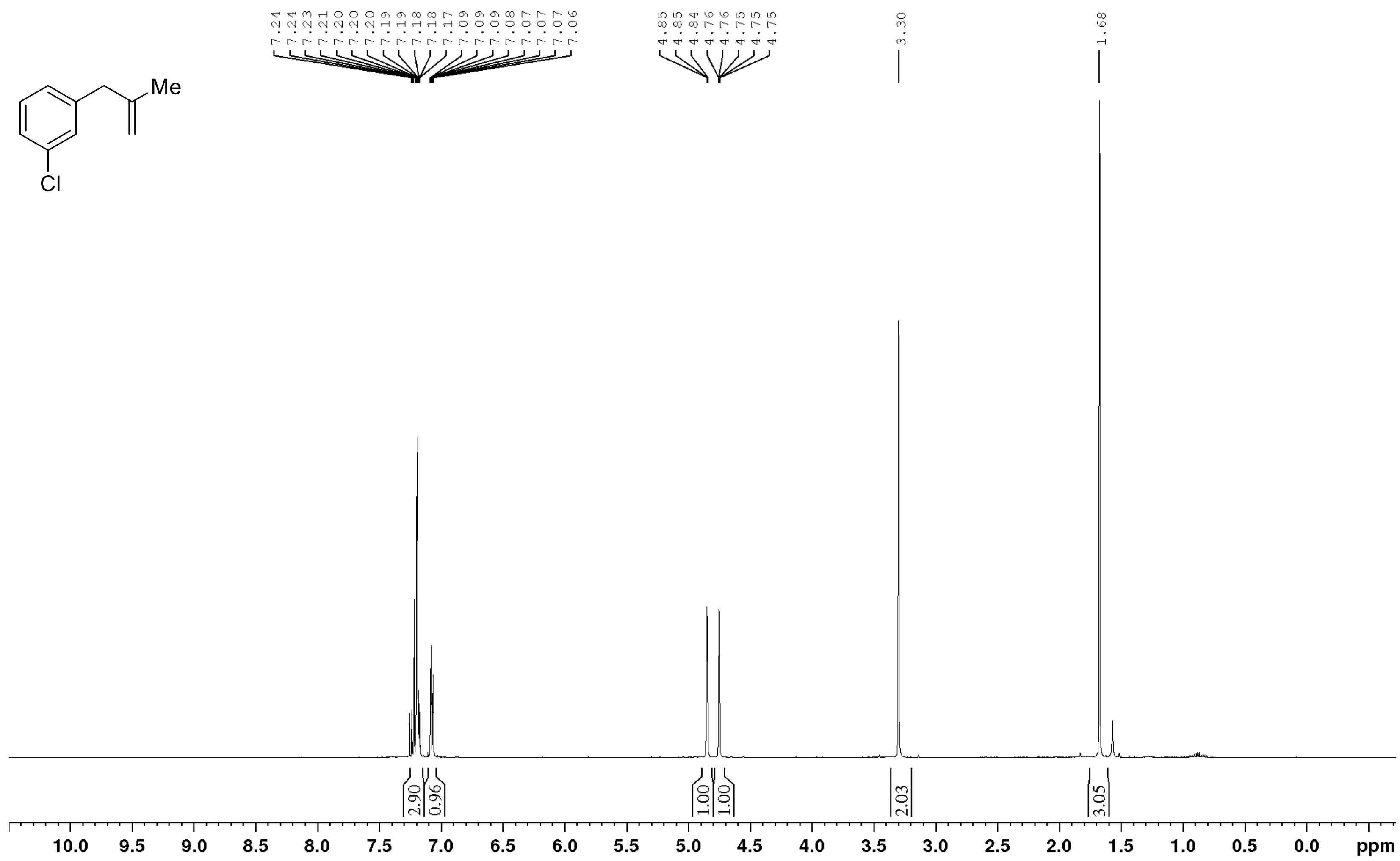

Figure S9. 1-Chloro-3-(2-methylallyl)benzene (**1k**): $^{13}C\{^{1}H\}$ NMR (101 MHz, $CDCl_3$, 298 K)

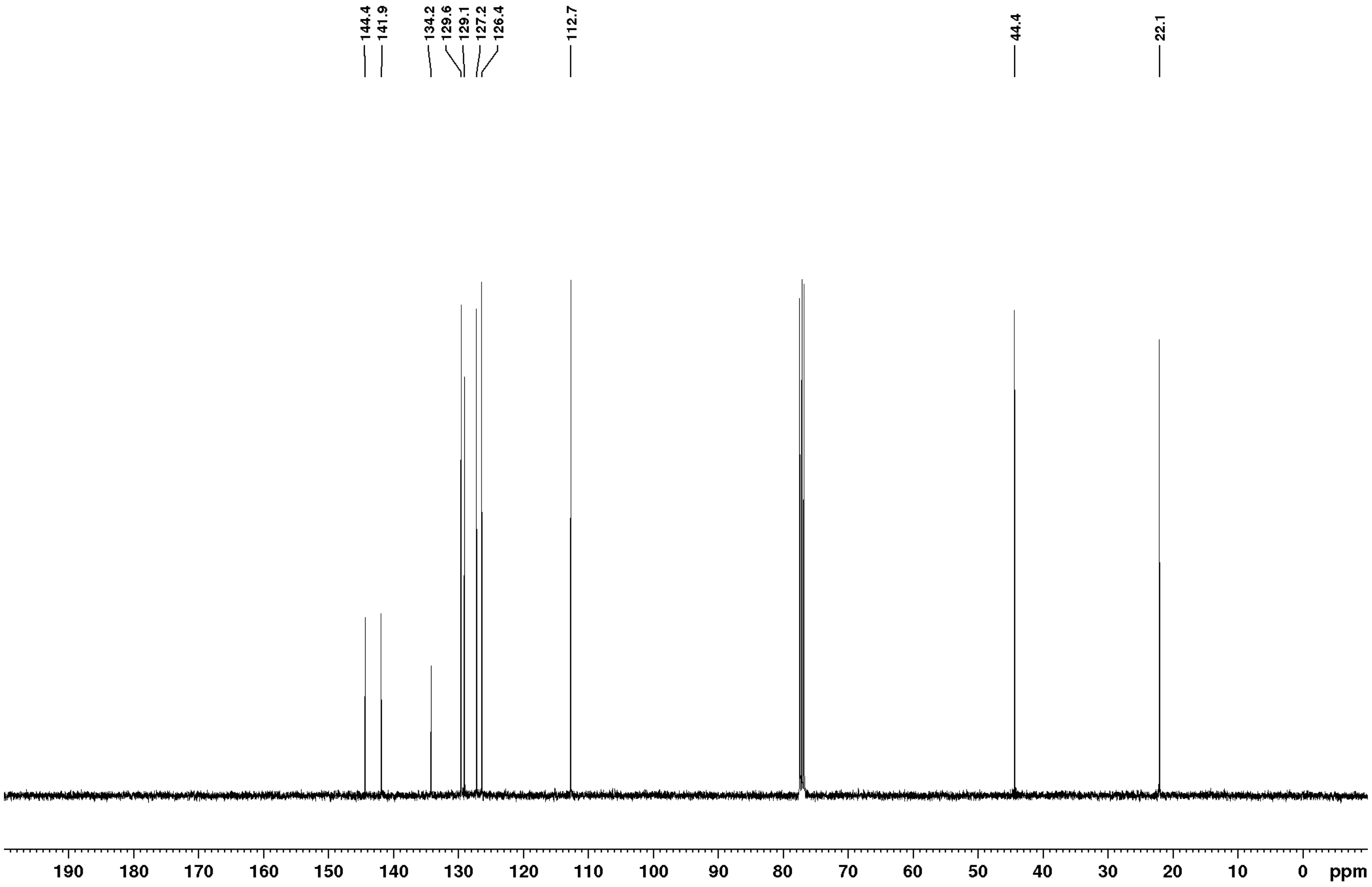

Figure S10. (2-Chloro-2-methylpropyl)benzene (3a): ^{1}H NMR spectrum (400 MHz, $CDCl_3$, 298 K)

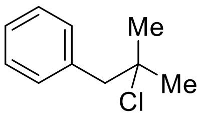

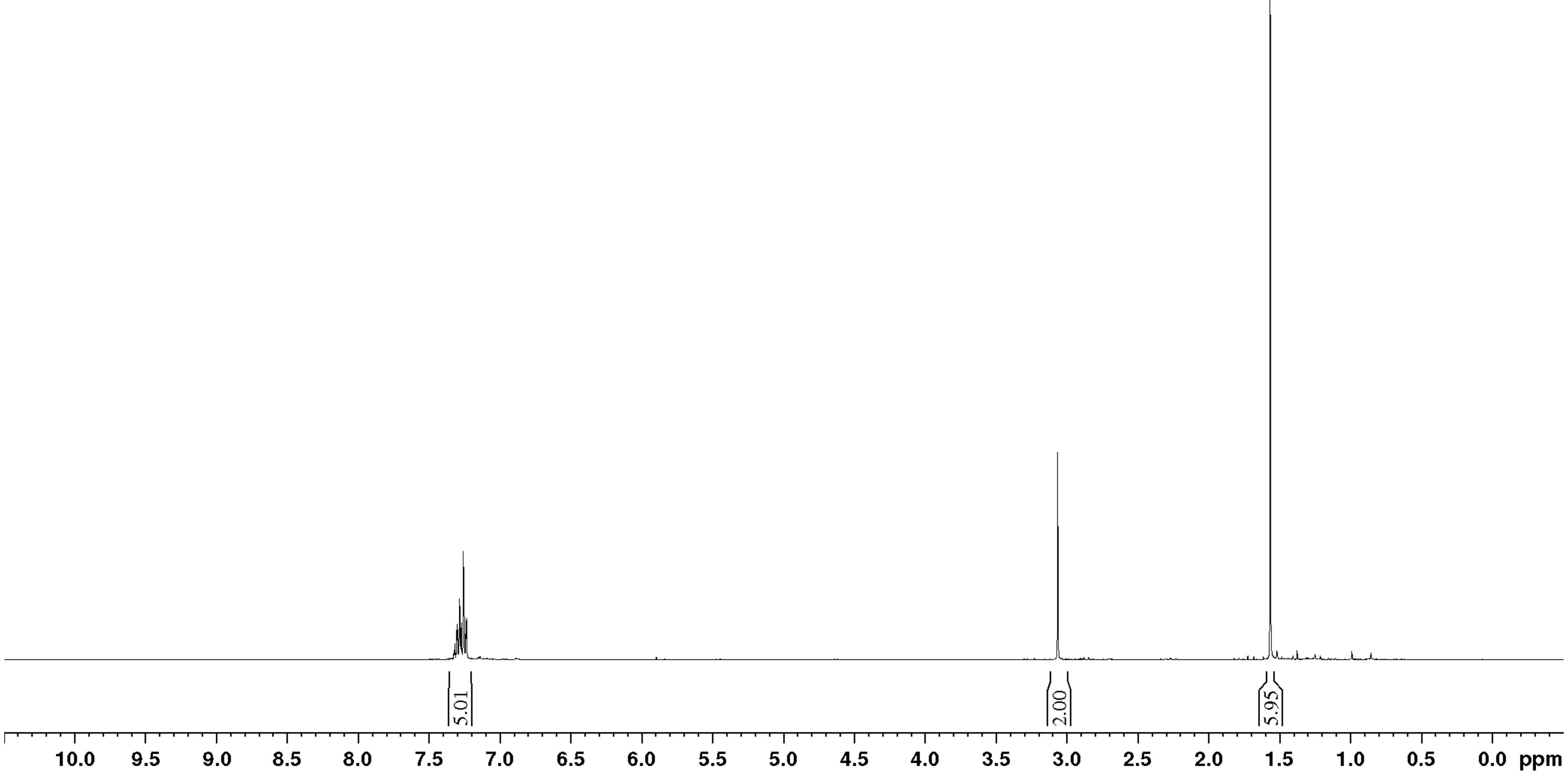

Figure S11. (2-Chloro-2-methylpropyl)benzene (3a): $^{13}C\{^{1}H\}$ NMR (101 MHz, $CDCl_3$, 298 K)

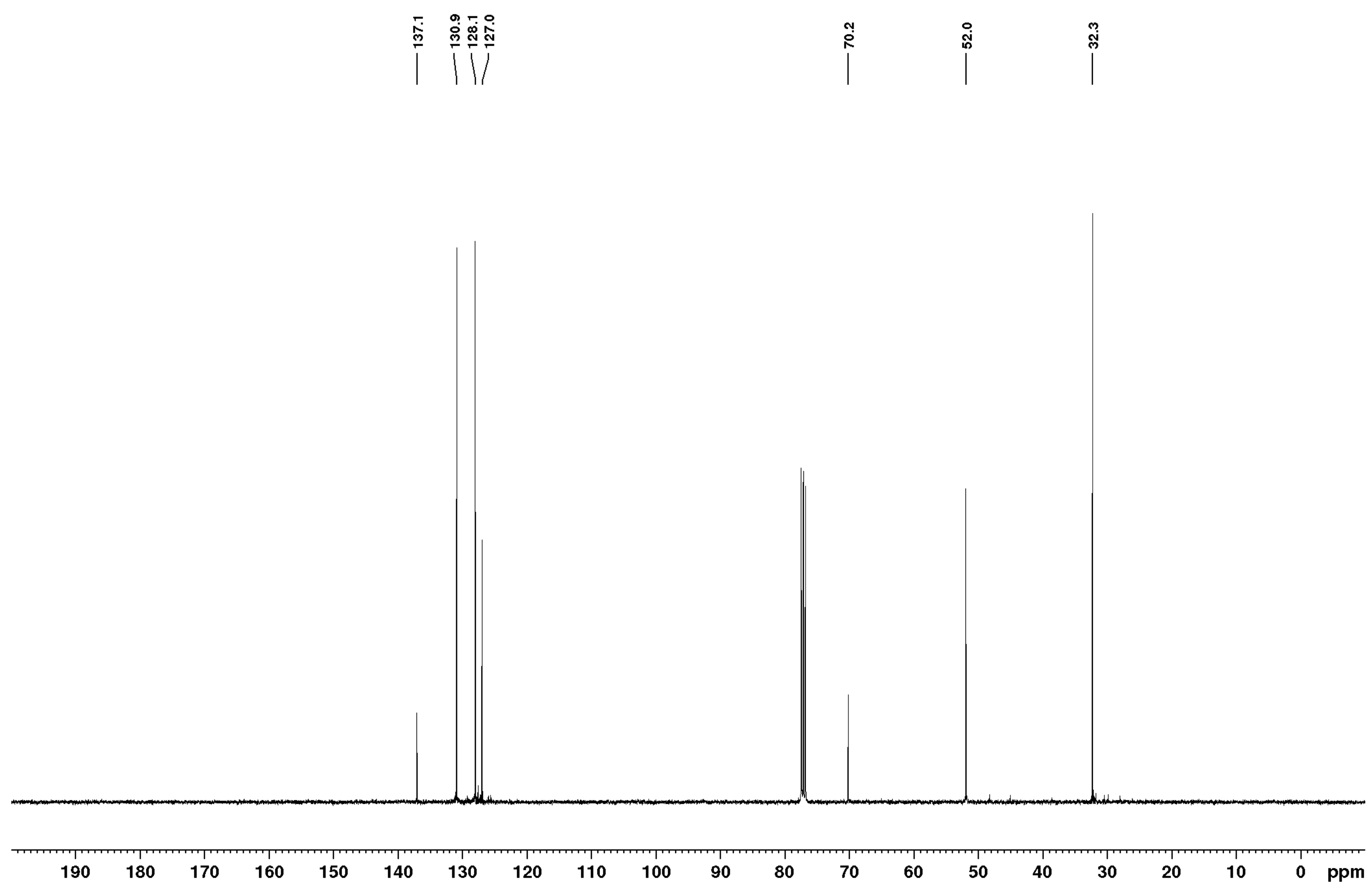

Figure S12. 1-(2-Chloro-2-methylpropyl)-4-methylbenzene (3b): ^{1}H NMR spectrum (400 MHz, $CDCl_3$, 298 K)

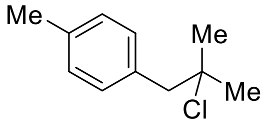

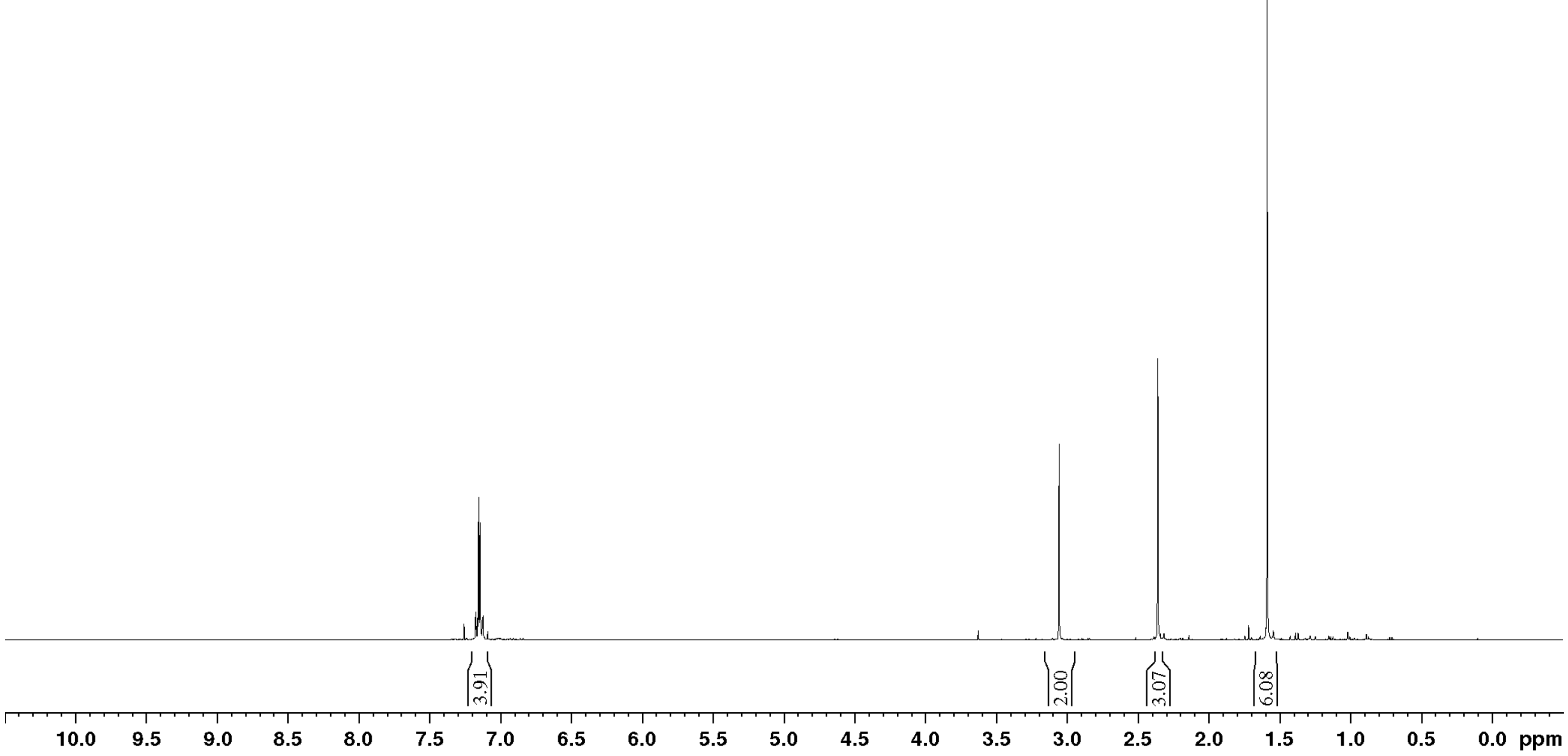

Figure S13. 1-(2-Chloro-2-methylpropyl)-4-methylbenzene (3b): $^{13}C\{^1H\}$ NMR (101 MHz, $CDCl_3$, 298 K)

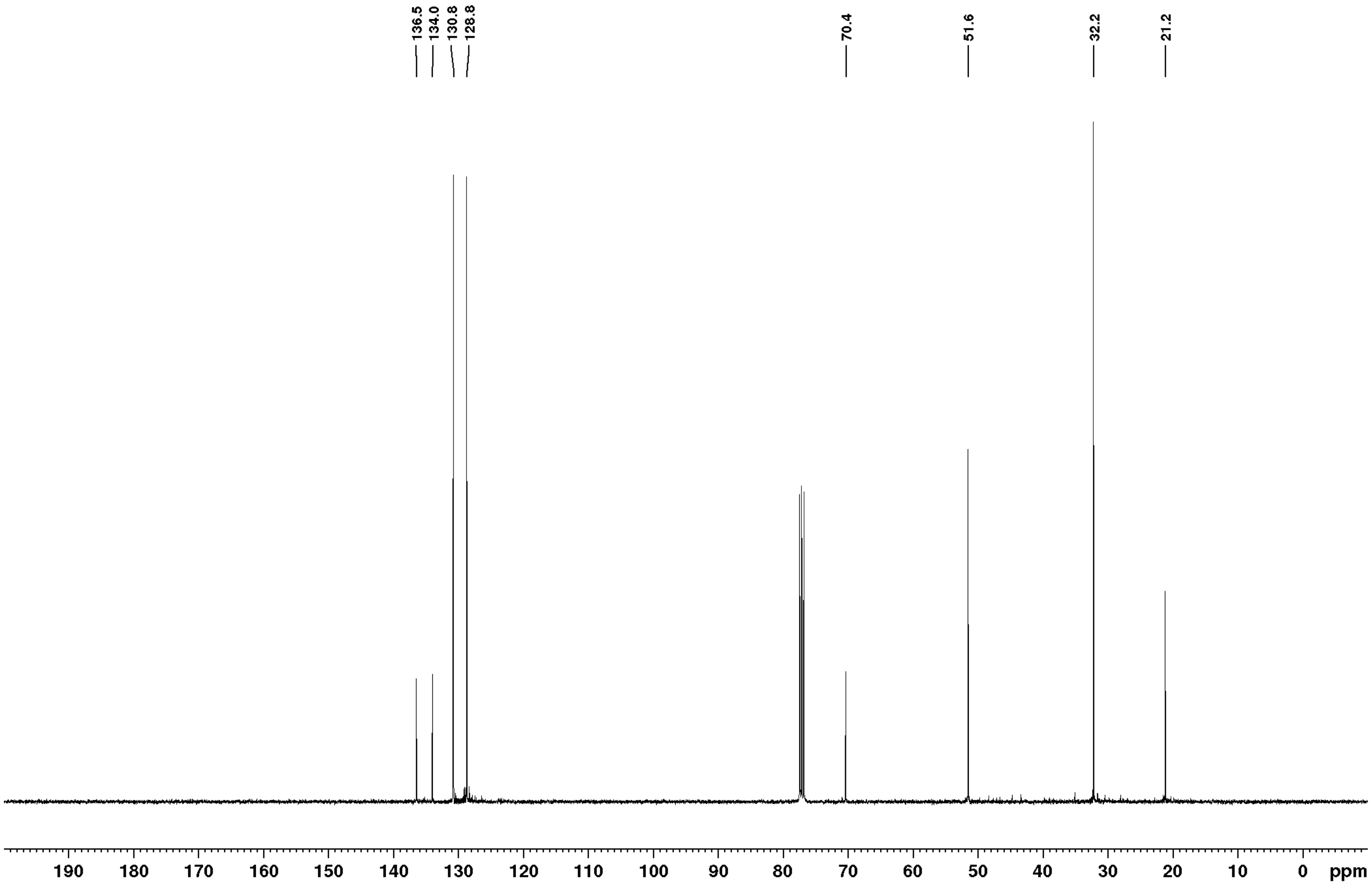

Figure S14. 1-(2-Chloro-2-methylpropyl)-4-methoxybenzene (3c): ^{1}H NMR spectrum (400 MHz, $CDCl_3$, 298 K)

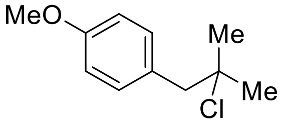

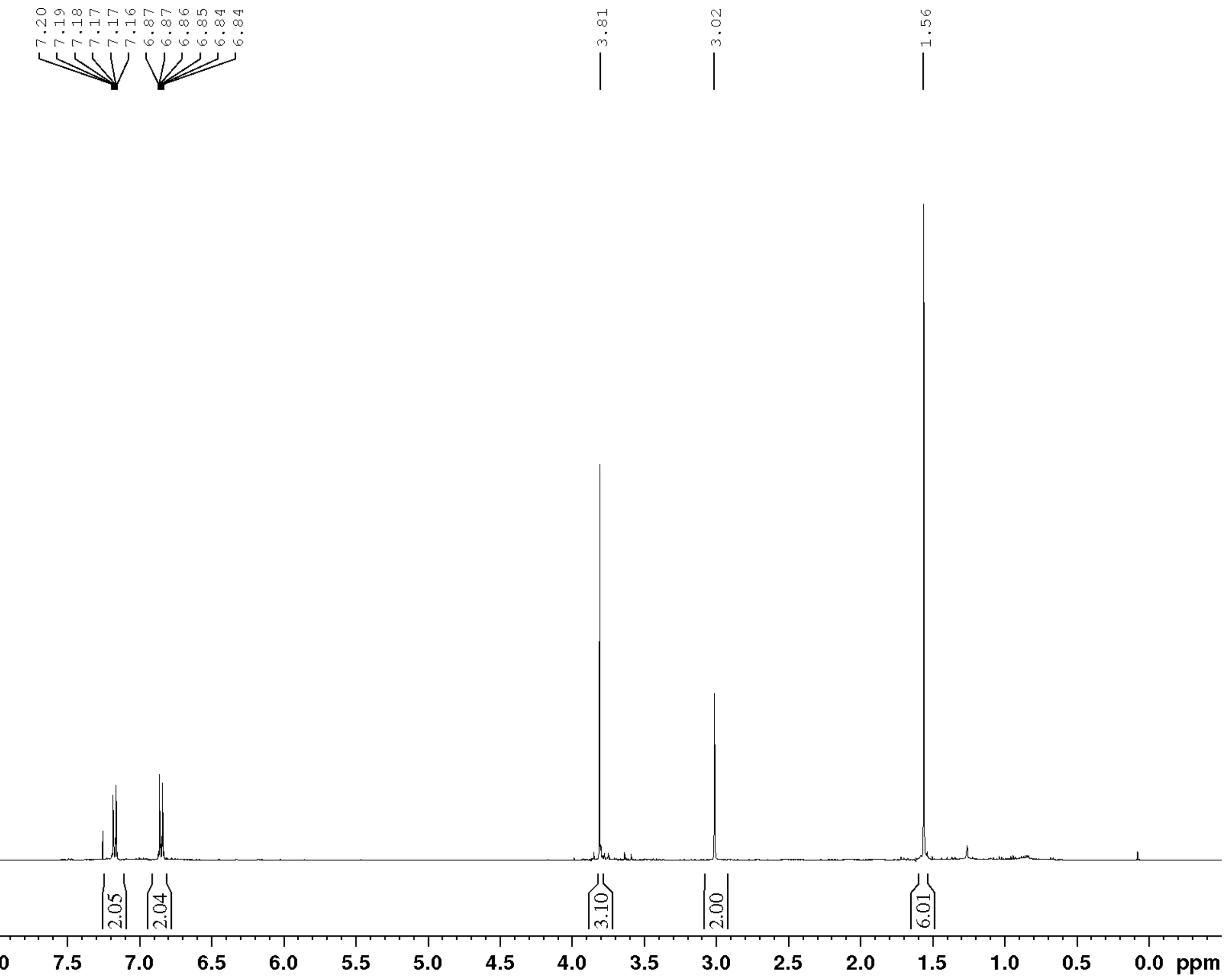

Figure S15. 1-(2-Chloro-2-methylpropyl)-4-methoxybenzene (3c): $^{13}C\{^{1}H\}$ NMR (101 MHz, $CDCl_3$, 298 K)

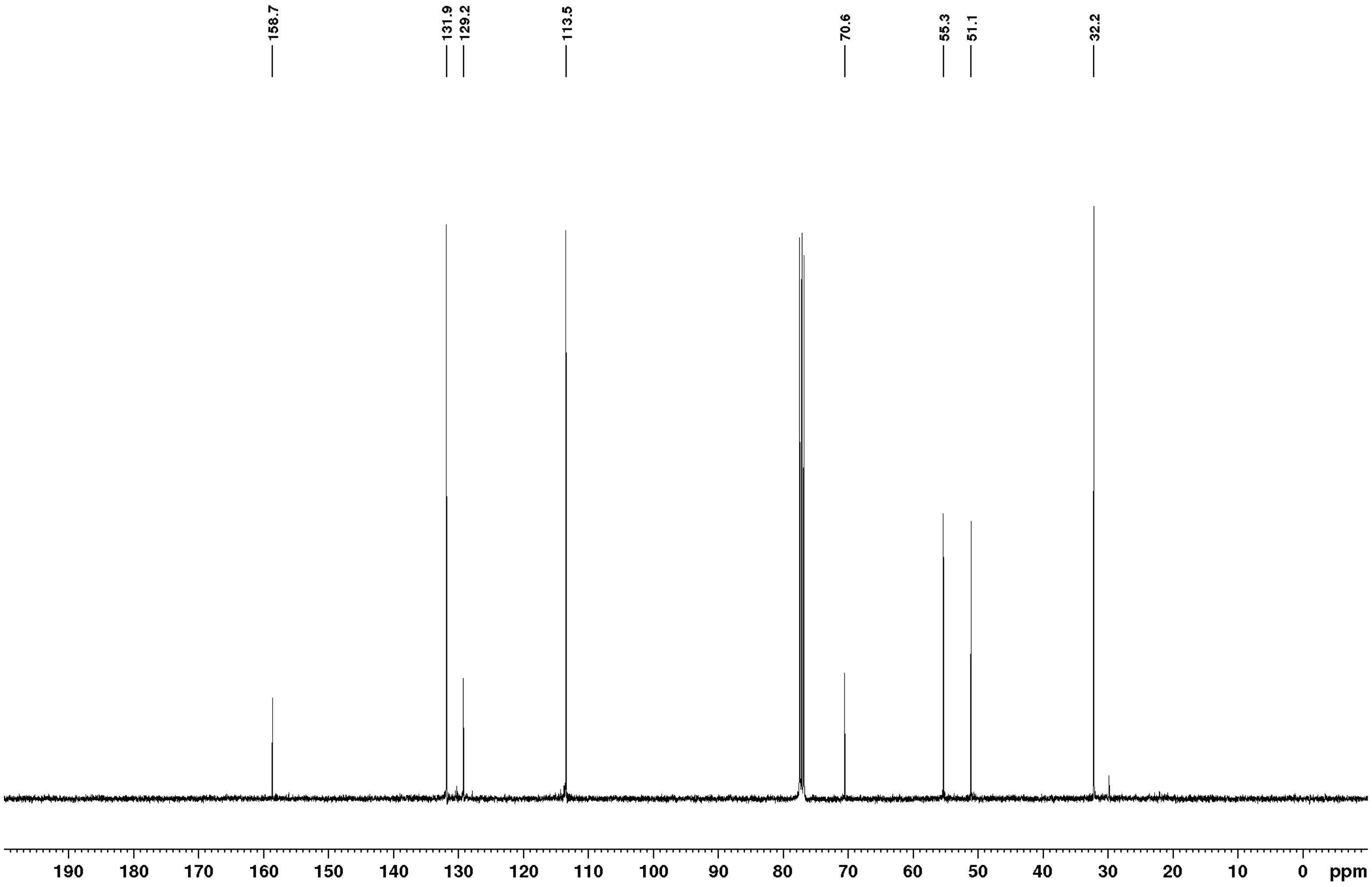

Figure S16. 1-Chloro-4-(2-chloro-2-methylpropyl)benzene (3d): ^{1}H NMR spectrum (400 MHz, $CDCl_3$, 298 K)

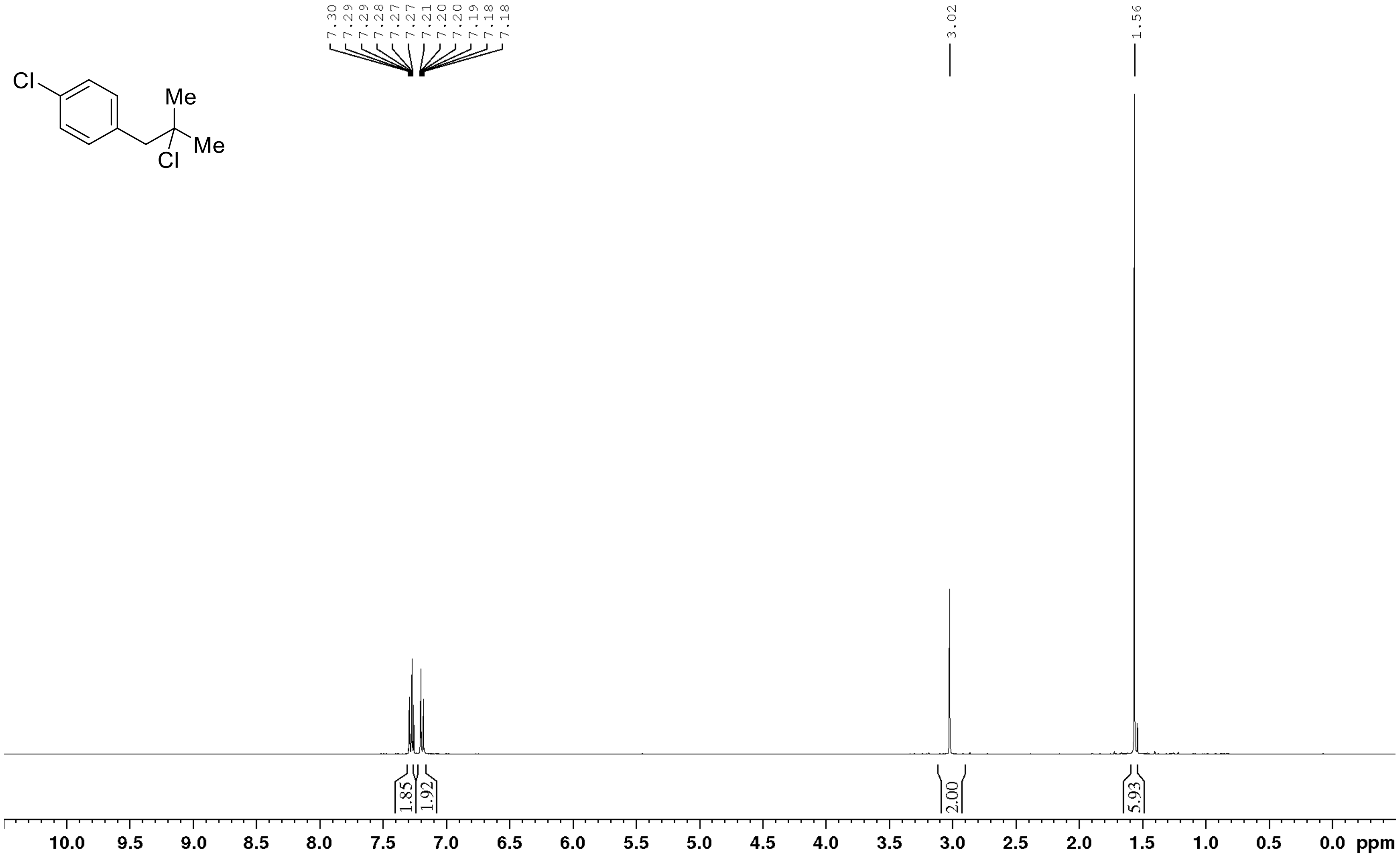

Figure S17. 1-Chloro-4-(2-chloro-2-methylpropyl)benzene (3d): $^{13}C\{^1H\}$ NMR (101 MHz, $CDCl_3$, 298 K)

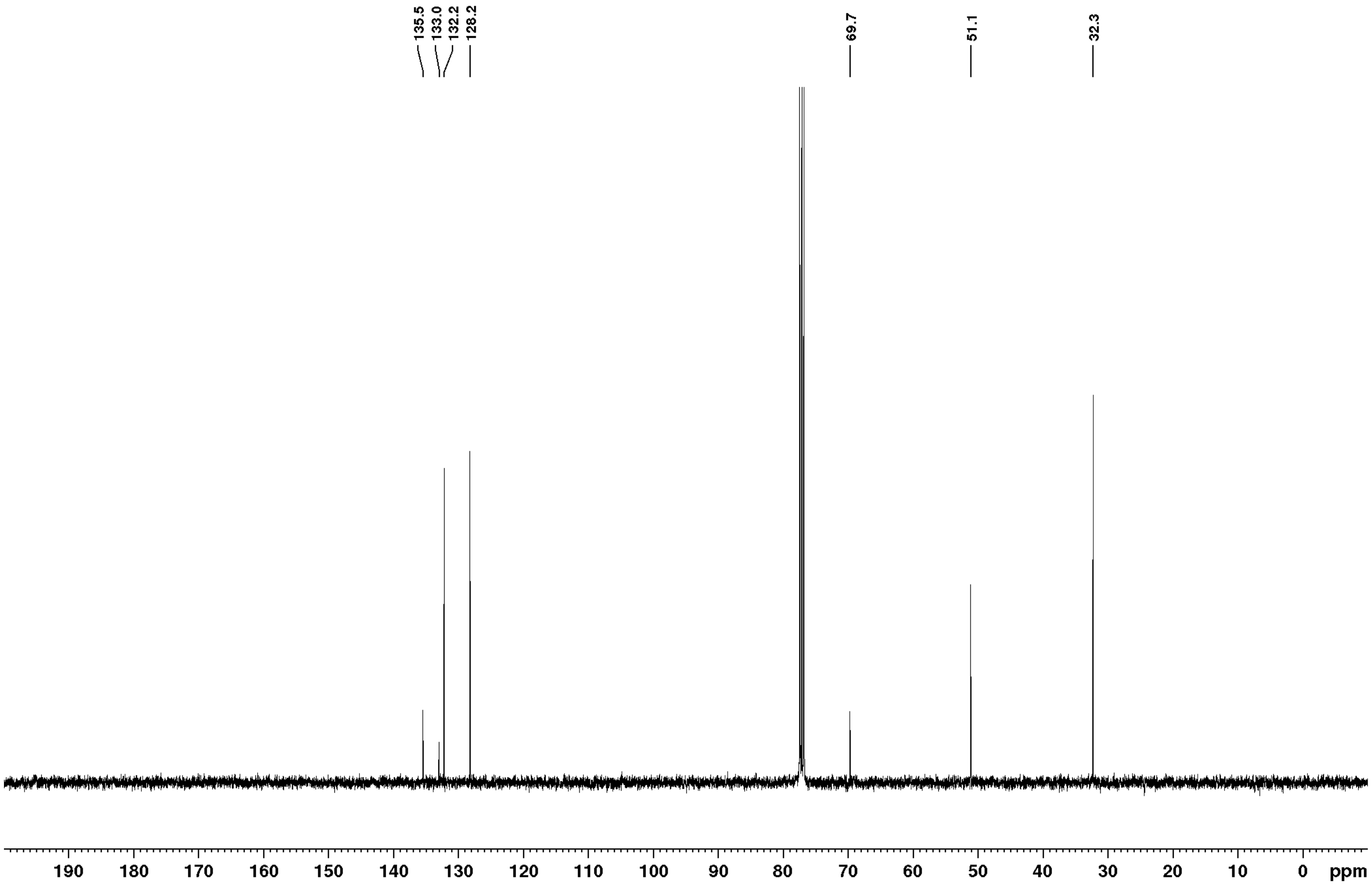

Figure S18. 1-Bromo-4-(2-chloro-2-methylpropyl)benzene (3e): ^{1}H NMR spectrum (400 MHz, $CDCl_3$, 298 K)

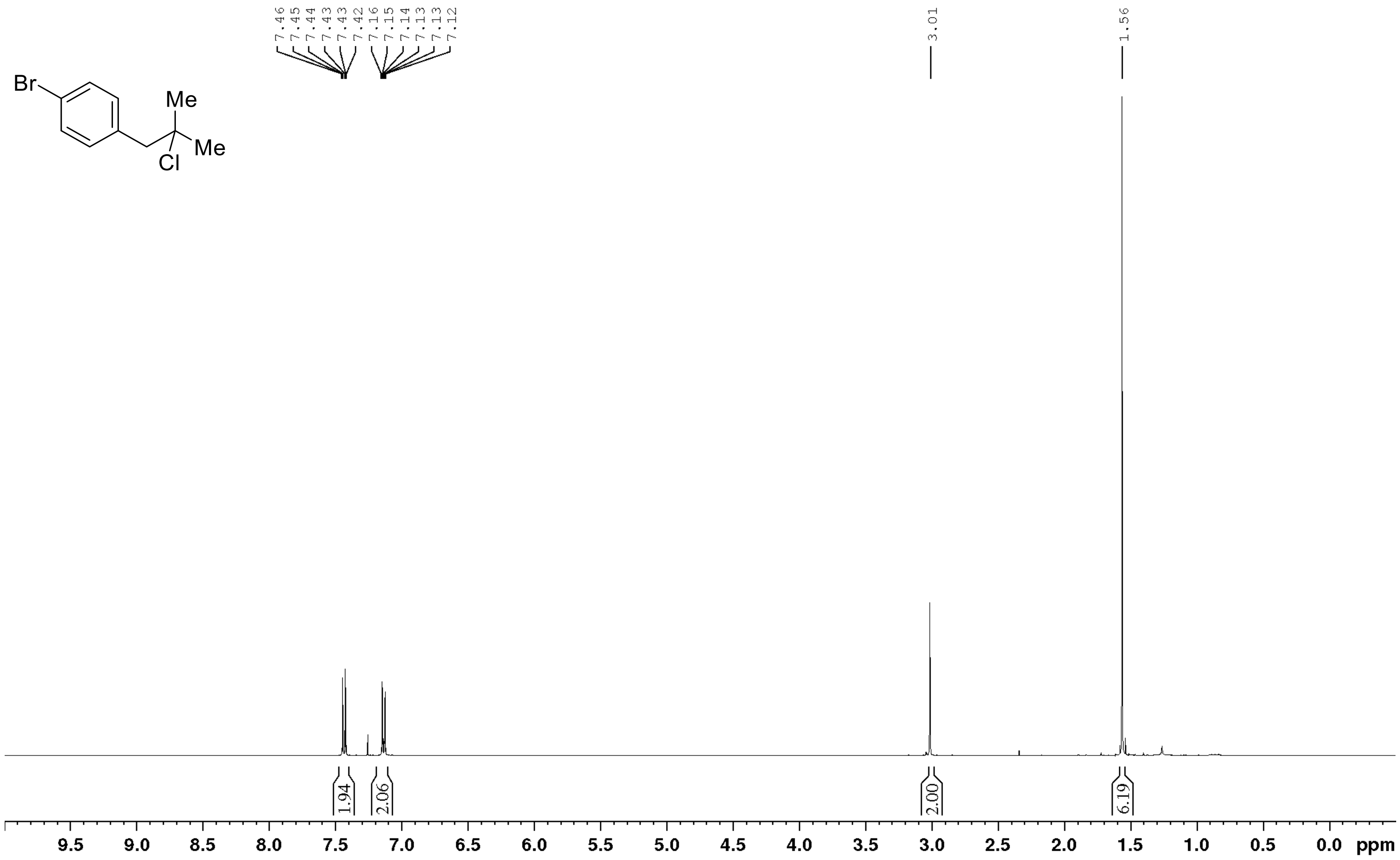

Figure S19. 1-Bromo-4-(2-chloro-2-methylpropyl)benzene (3e): $^{13}C\{^{1}H\}$ NMR (101 MHz, $CDCl_3$, 298 K)

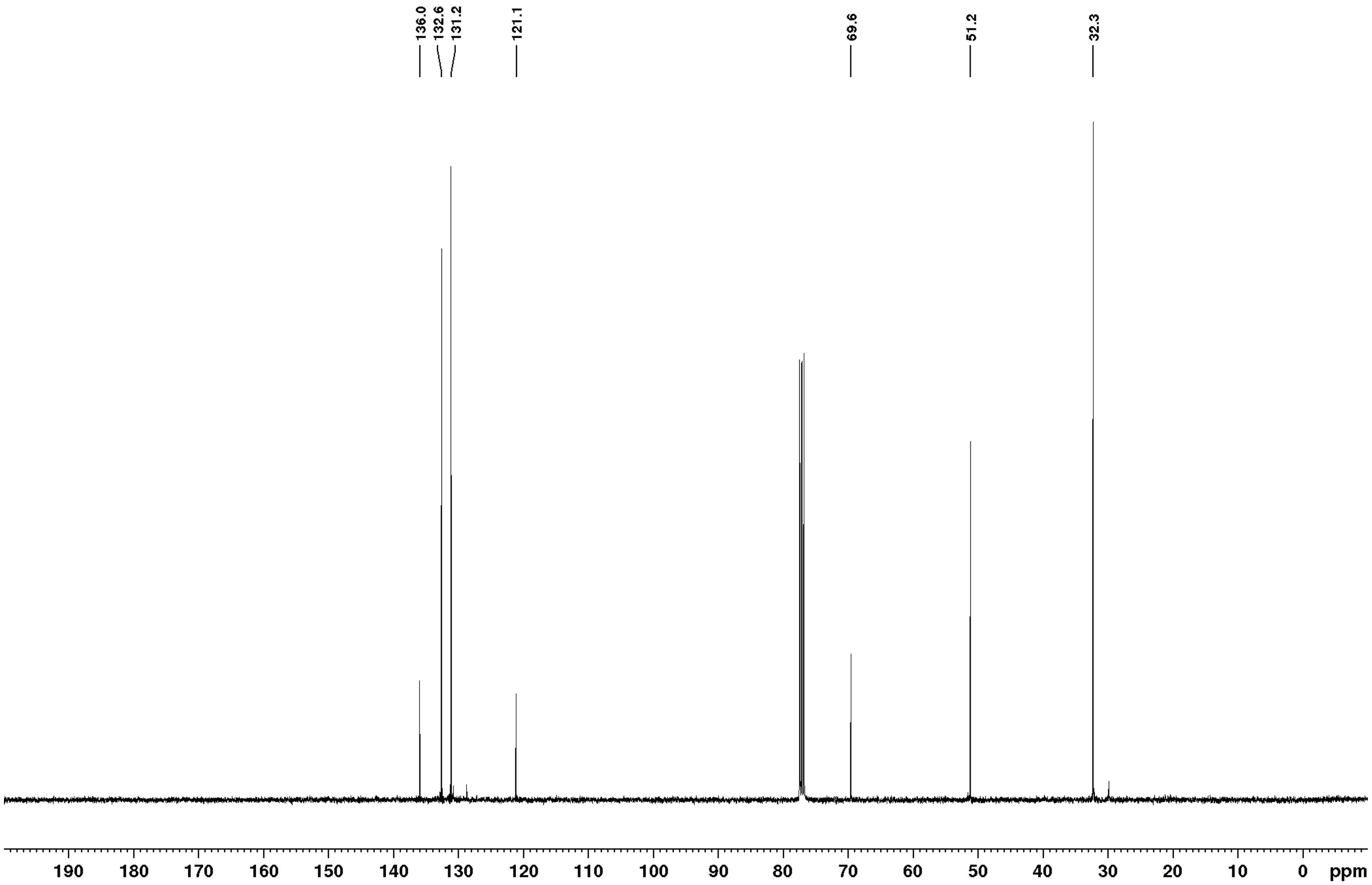

Figure S20. 1-(2-Chloro-2-methylpropyl)-4-(trifluoromethyl)benzene (3f): ^{1}H NMR spectrum (400 MHz, $CDCl_3$, 298 K)

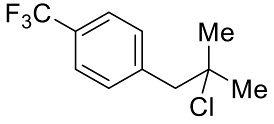

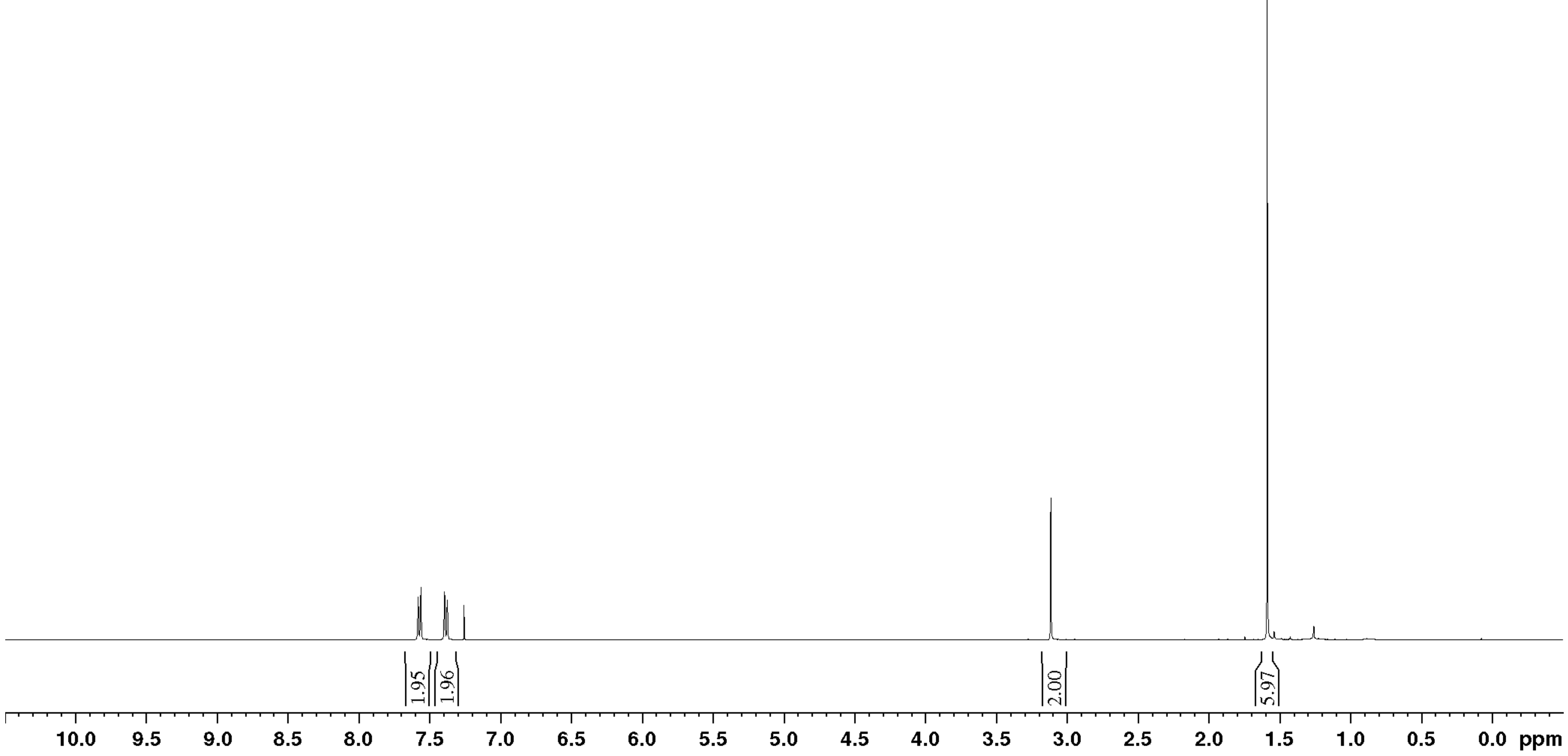

Figure S21. 1-(2-Chloro-2-methylpropyl)-4-(trifluoromethyl)benzene (3f): $^{13}C\{^1H\}$ NMR (101 MHz, $CDCl_3$, 298 K)

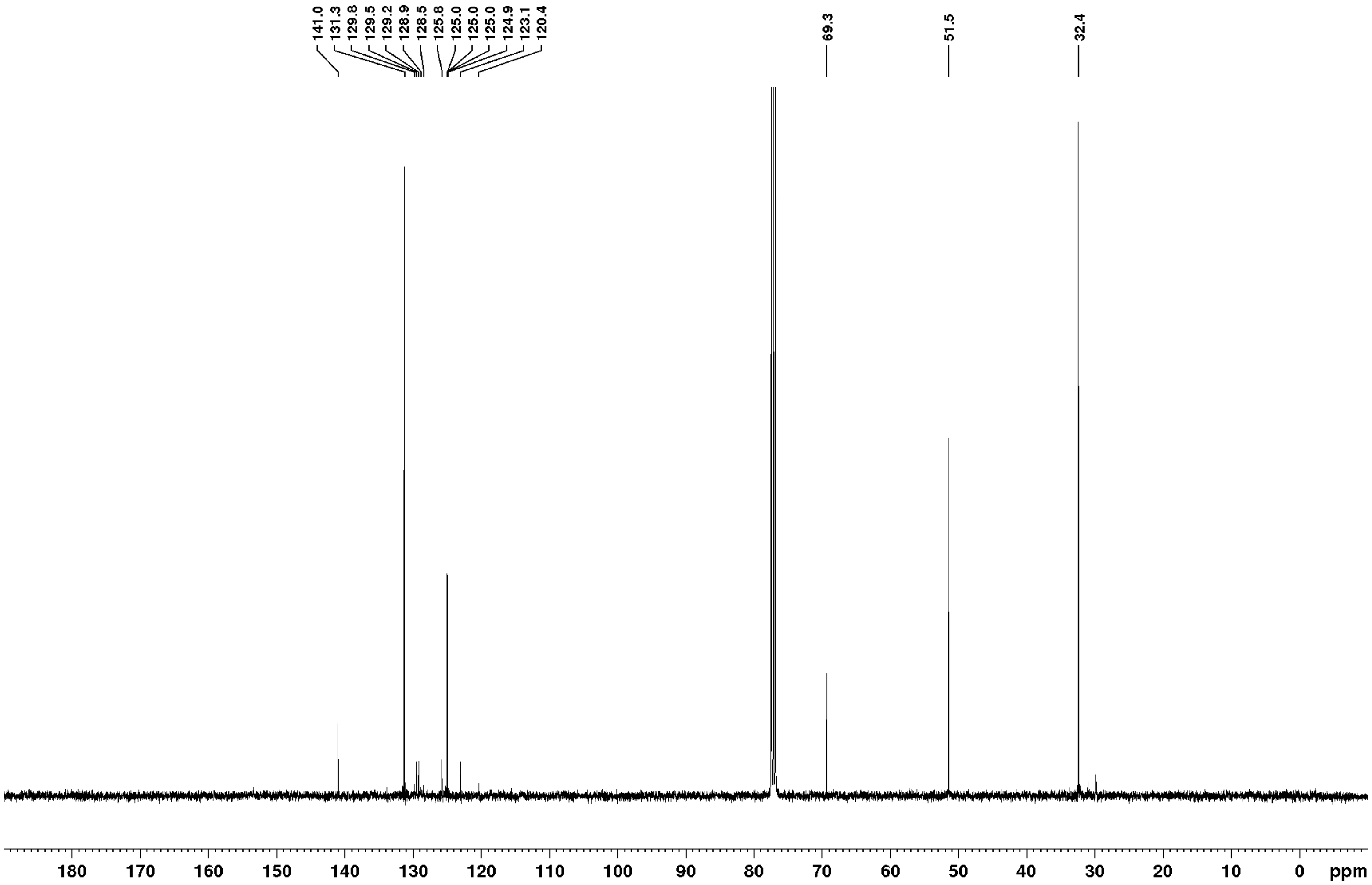

Figure S22. 1-(2-Chloro-2-methylpropyl)-4-(trifluoromethyl)benzene (3f): ^{19}F NMR (695 MHz, $CDCl_3$, 298 K)

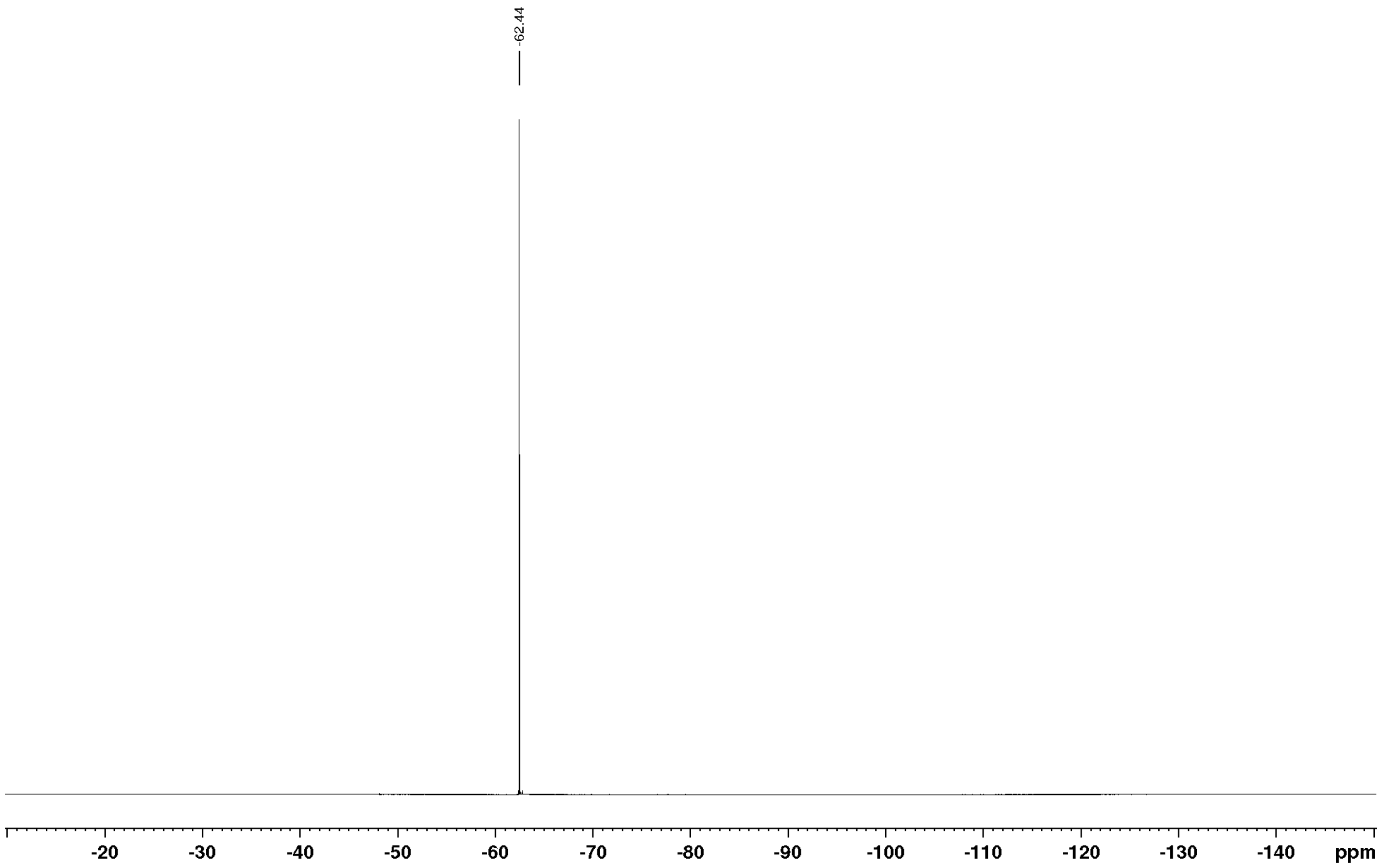

Figure S23. Methyl 4-(2-chloro-2-methylpropyl)benzoate (3g): ^{1}H NMR spectrum (400 MHz, $CDCl_3$, 298 K)

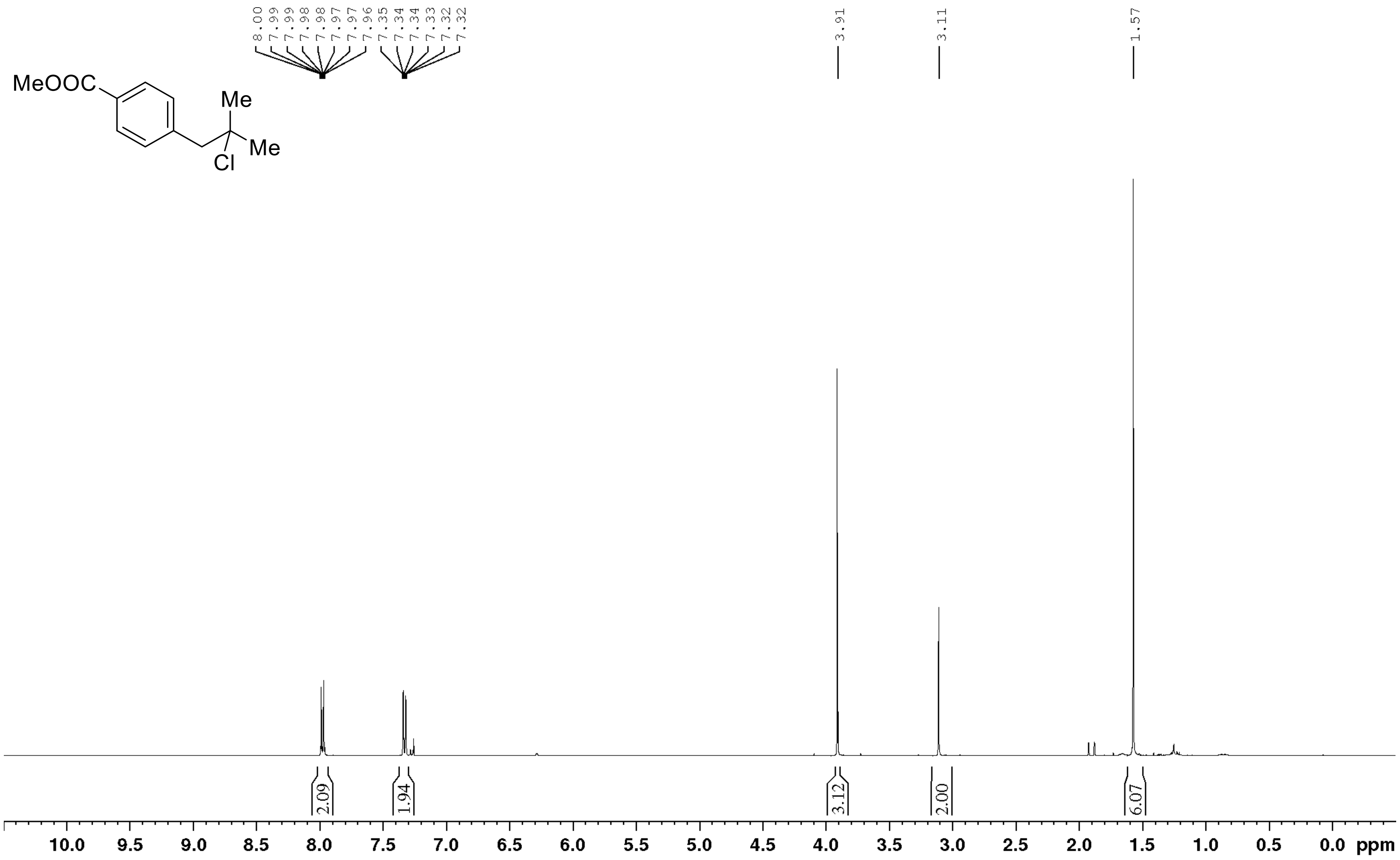

Figure S24. Methyl 4-(2-chloro-2-methylpropyl)benzoate (3g): $^{13}C\{^{1}H\}$ NMR (101 MHz, $CDCl_3$, 298 K)

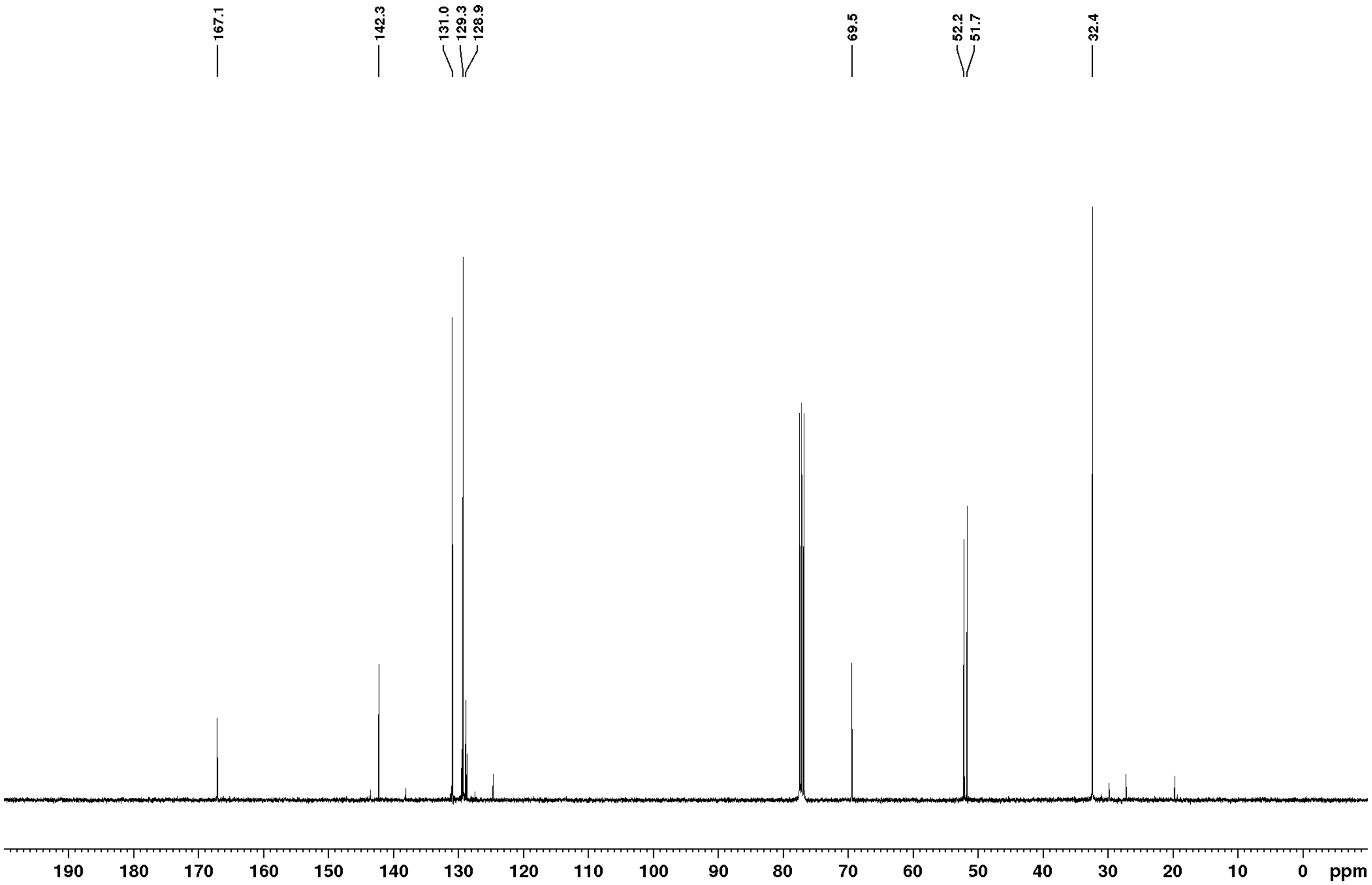

Figure S25. 1-(2-Chloro-2-methylpropyl)-2-methylbenzene (3h): ^{1}H NMR spectrum (400 MHz, $CDCl_3$, 298 K)

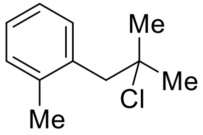

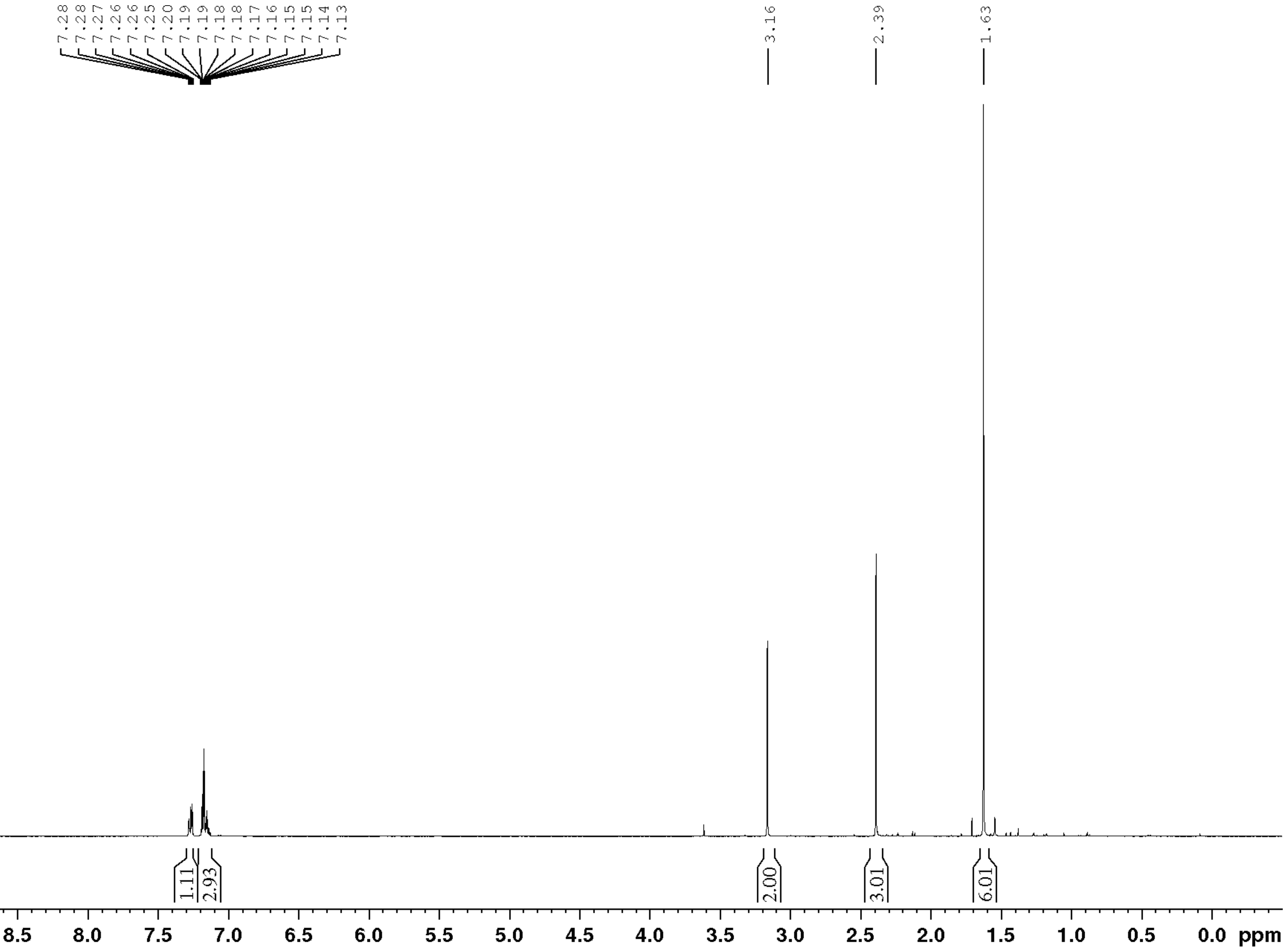

Figure S26. 1-(2-Chloro-2-methylpropyl)-2-methylbenzene (3h): $^{13}C\{^{1}H\}$ NMR (101 MHz, $CDCl_3$, 298 K)

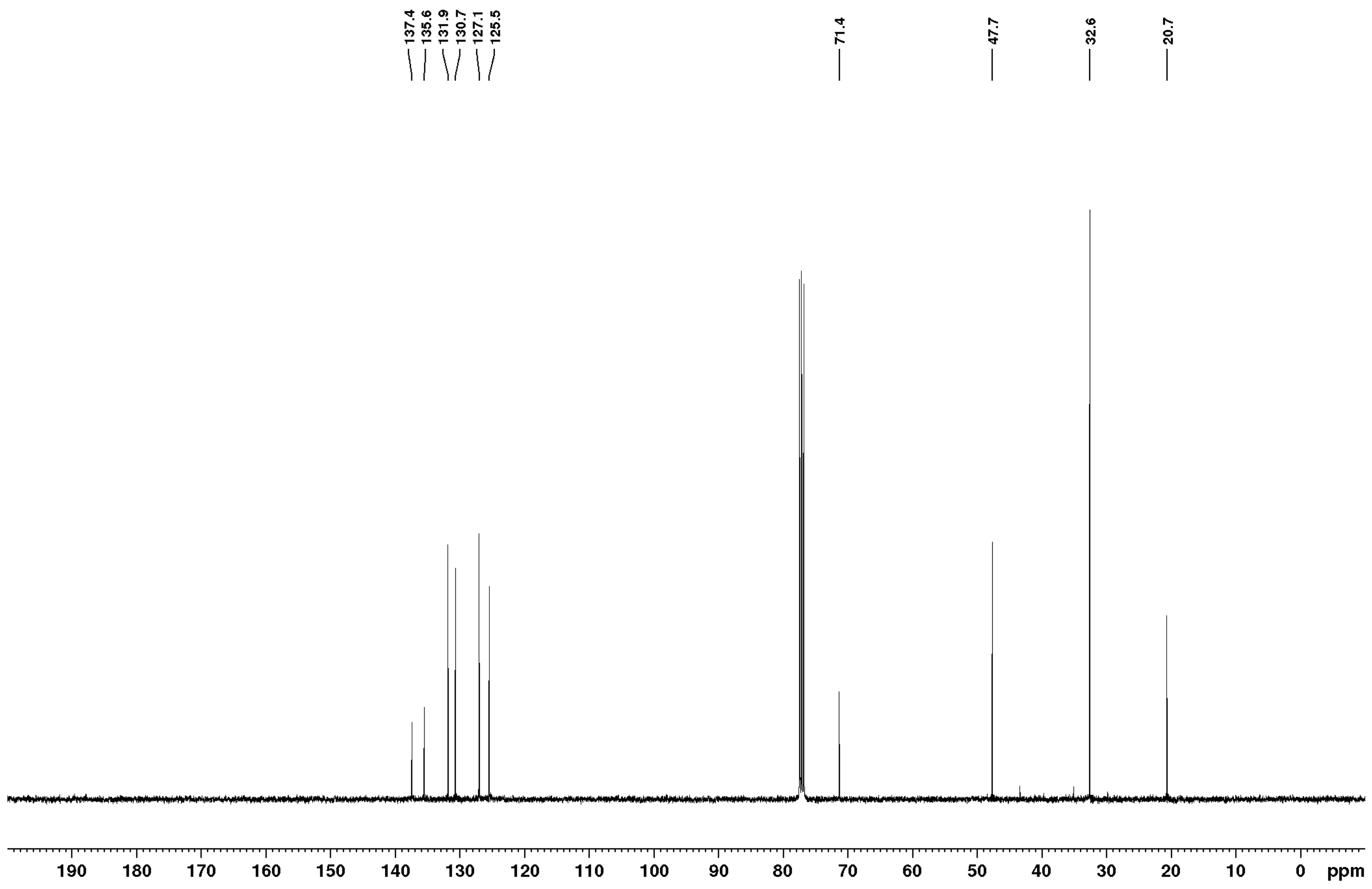

Figure S27. 1-Bromo-2-(2-chloro-2-methylpropyl)benzene (3i): ^{1}H NMR spectrum (400 MHz, $CDCl_3$, 298 K)

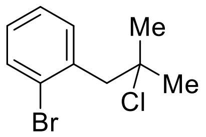

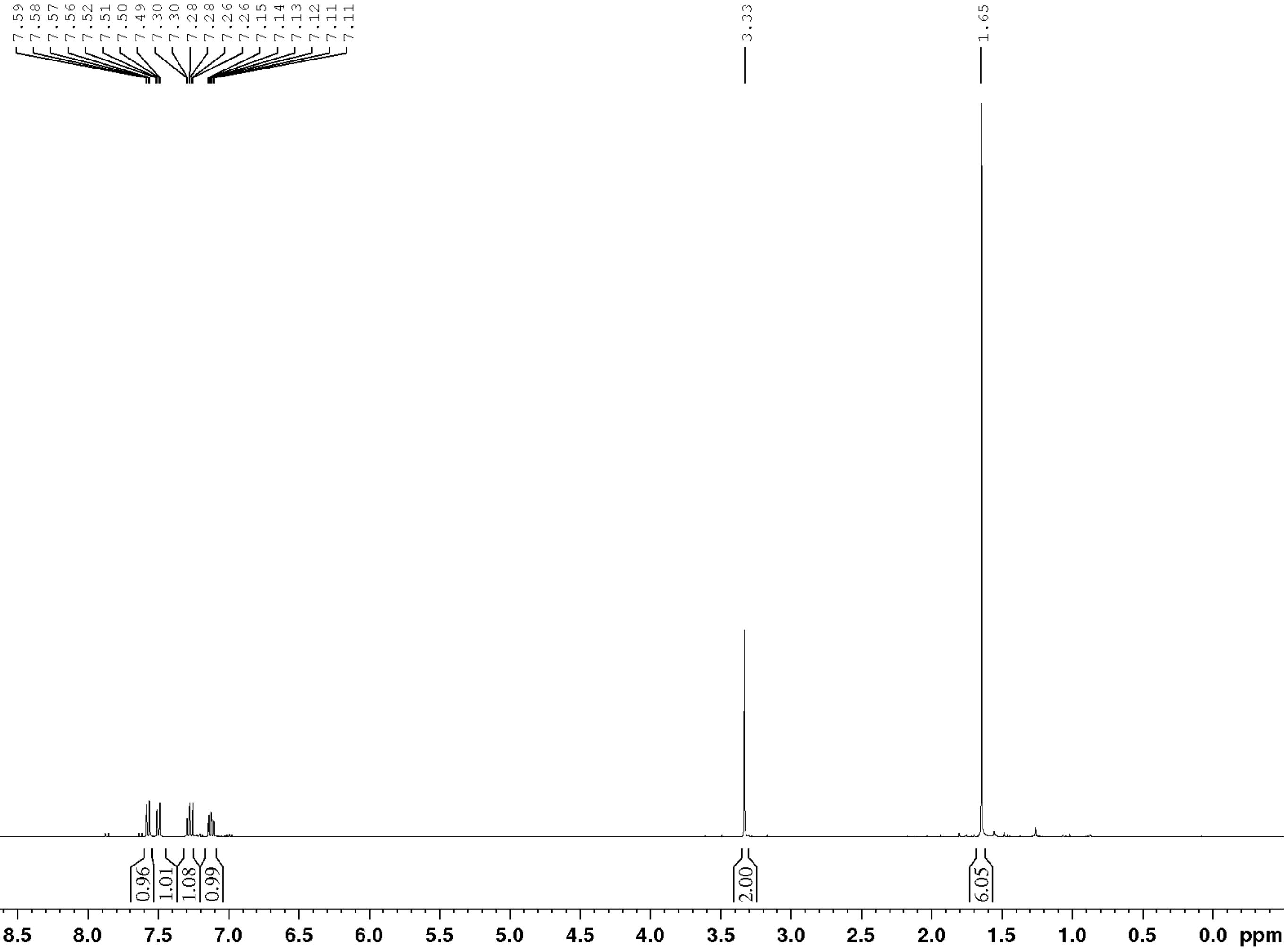

Figure S28. 1-Bromo-2-(2-chloro-2-methylpropyl)benzene (3i): $^{13}C\{^{1}H\}$ NMR (101 MHz, $CDCl_3$, 298 K)

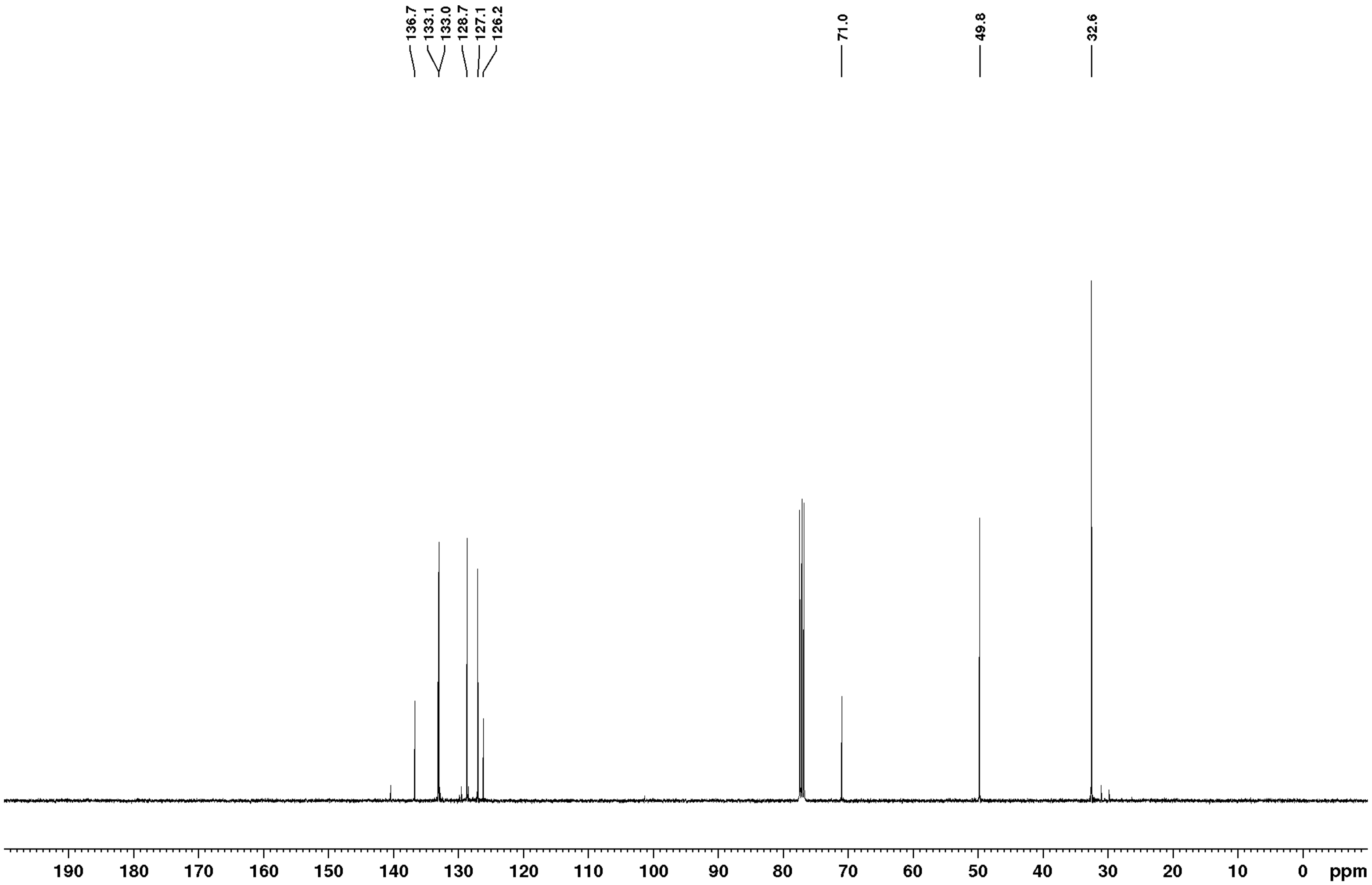

Figure S29. 1-(2-Chloro-2-methylpropyl)-3-methylbenzene (3j) : ^{1}H NMR spectrum (400 MHz, $CDCl_3$, 298 K)

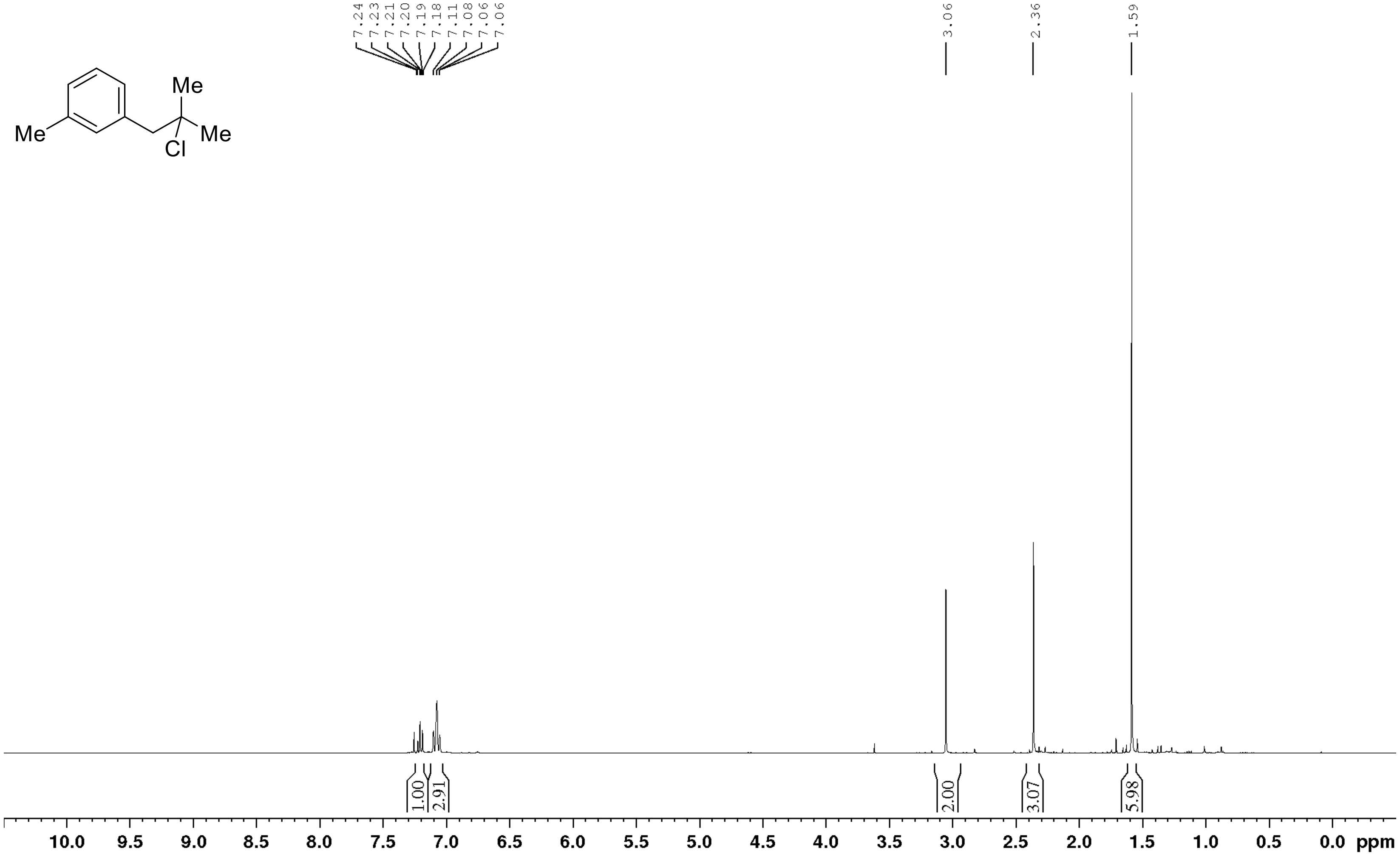

Figure S30. 1-(2-Chloro-2-methylpropyl)-3-methylbenzene (3j): $^{13}C\{^{1}H\}$ NMR (101 MHz, $CDCl_3$, 298 K)

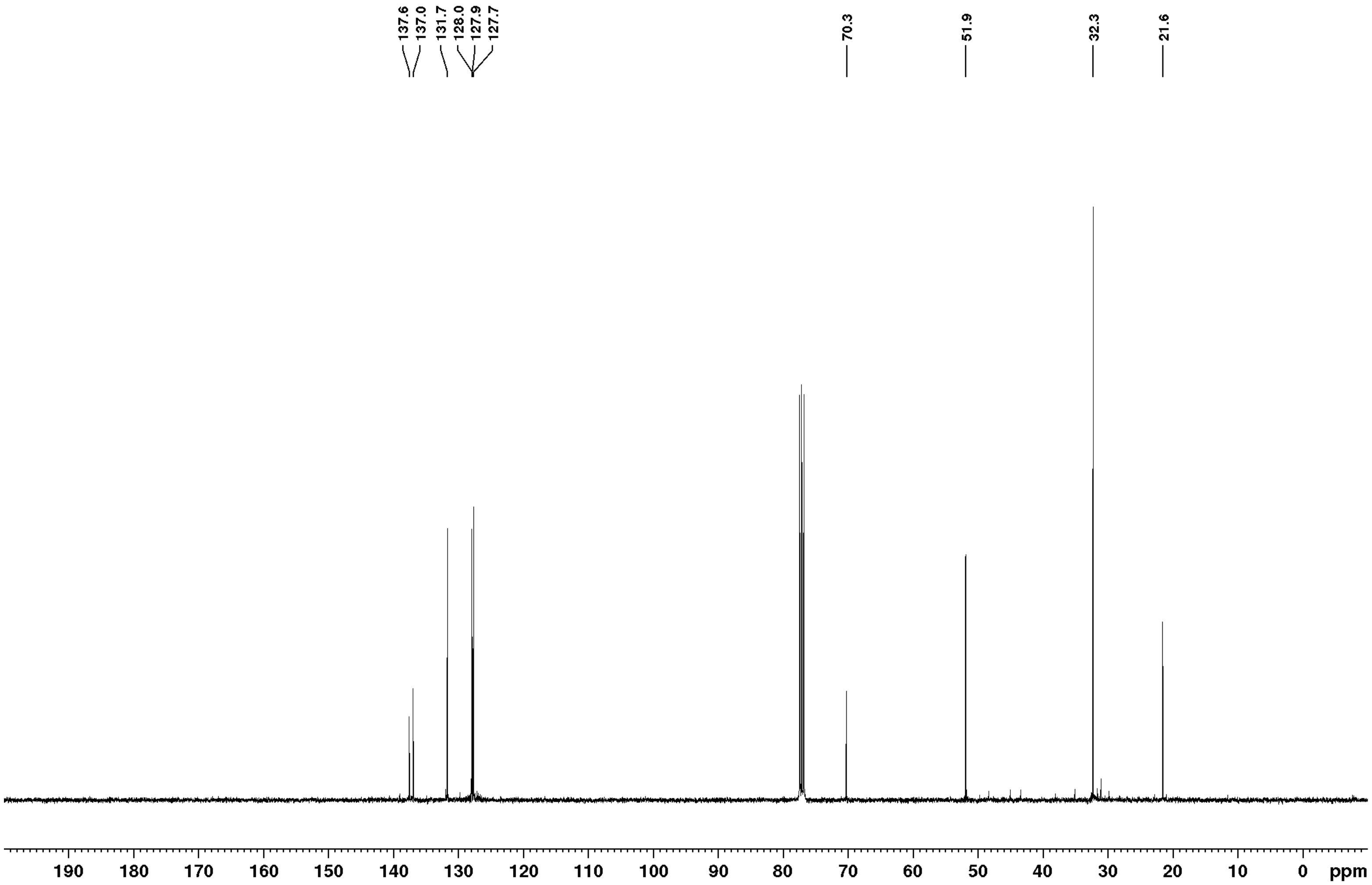

Figure S31. 1-Chloro-3-(2-chloro-2-methylpropyl)benzene (3k): ^{1}H NMR spectrum (400 MHz, $CDCl_3$, 298 K)

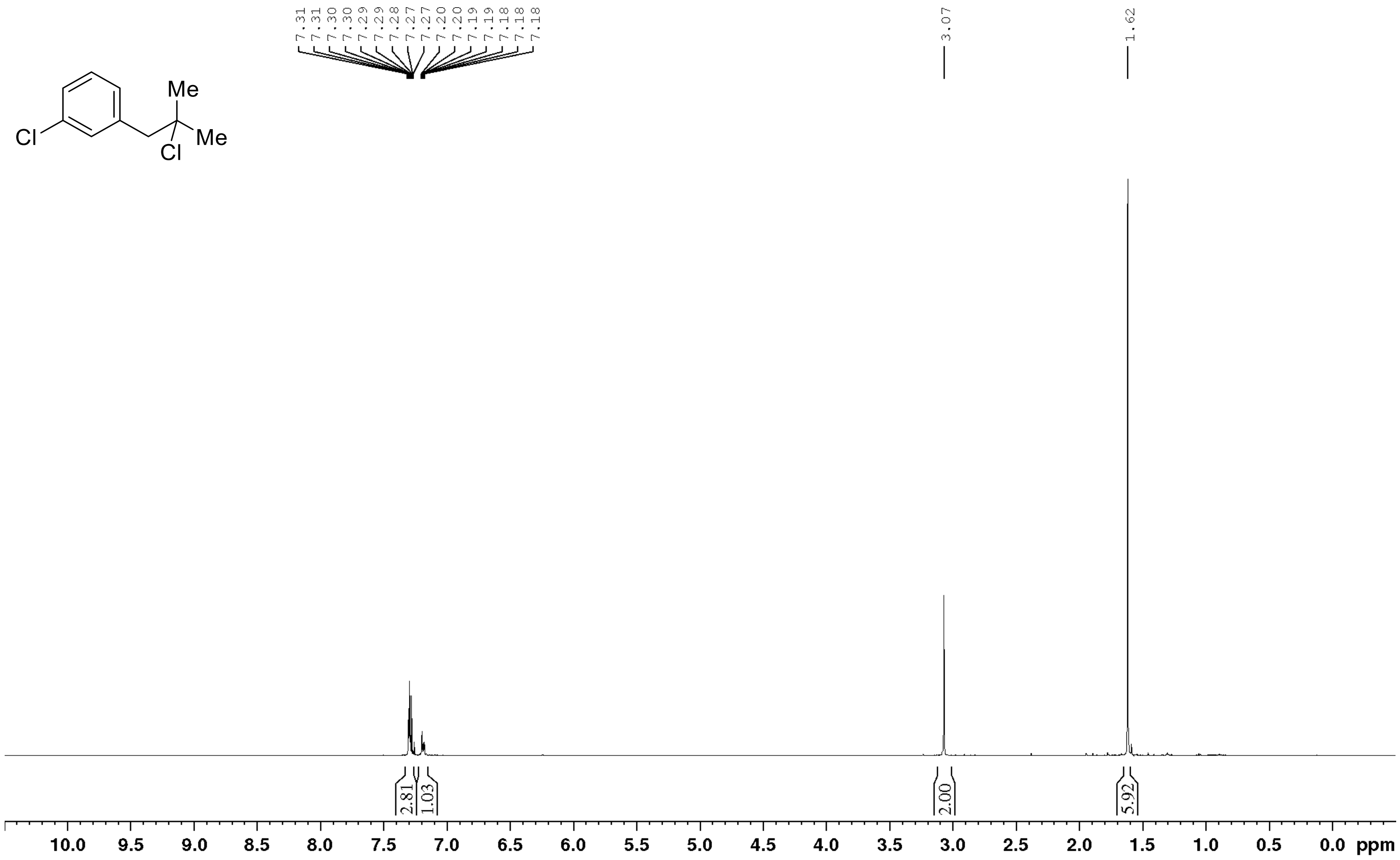

Figure S32. 1-Chloro-3-(2-chloro-2-methylpropyl)benzene (3k): $^{13}C\{^{1}H\}$ NMR (101 MHz, $CDCl_3$, 298 K)

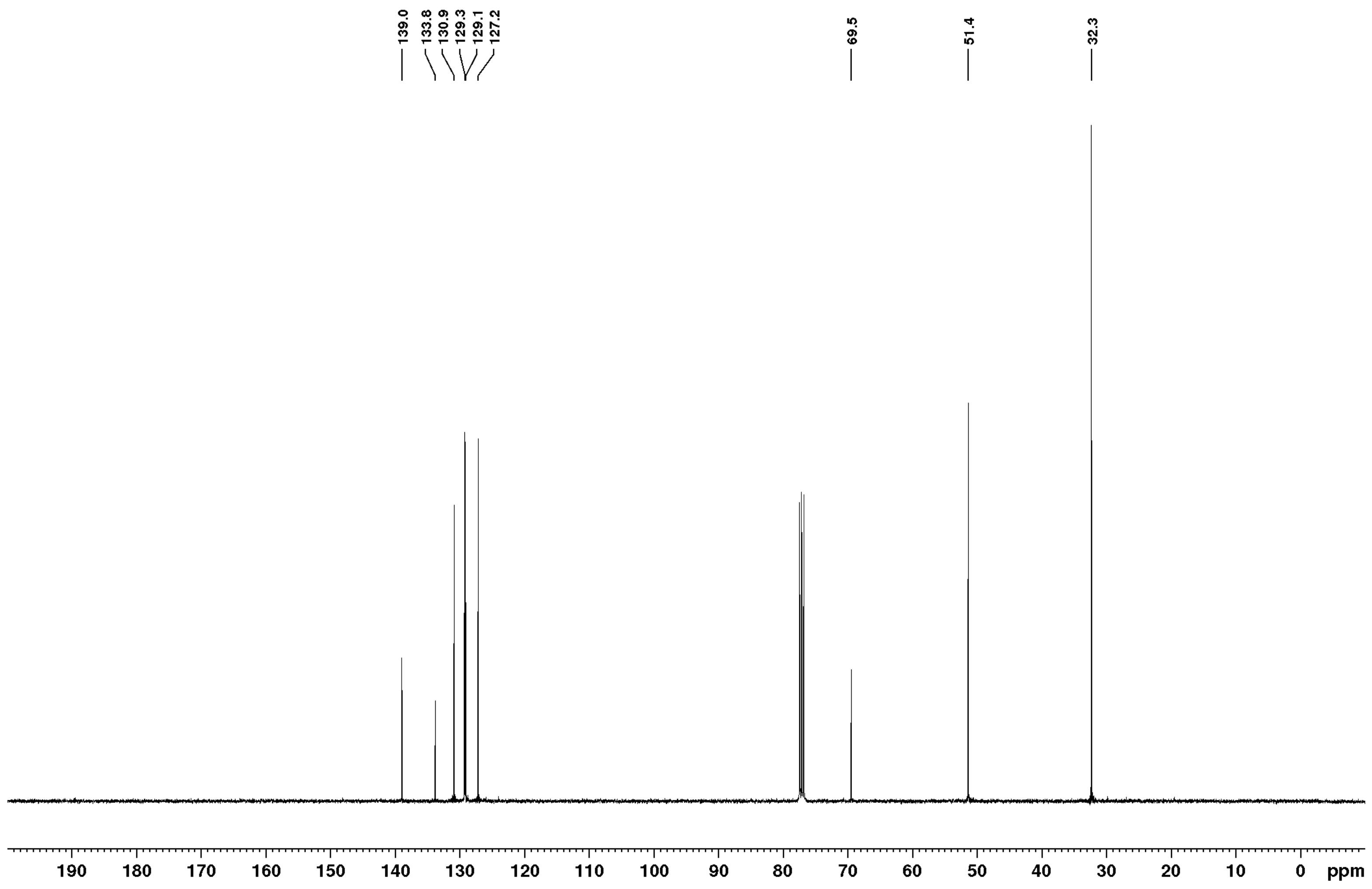

Figure S33. 2-(2-Chloro-2-methylpropyl)-1,3,5-trimethylbenzene (3l): ^{1}H NMR spectrum (400 MHz, $CDCl_3$, 298 K)

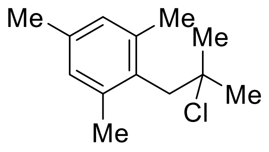

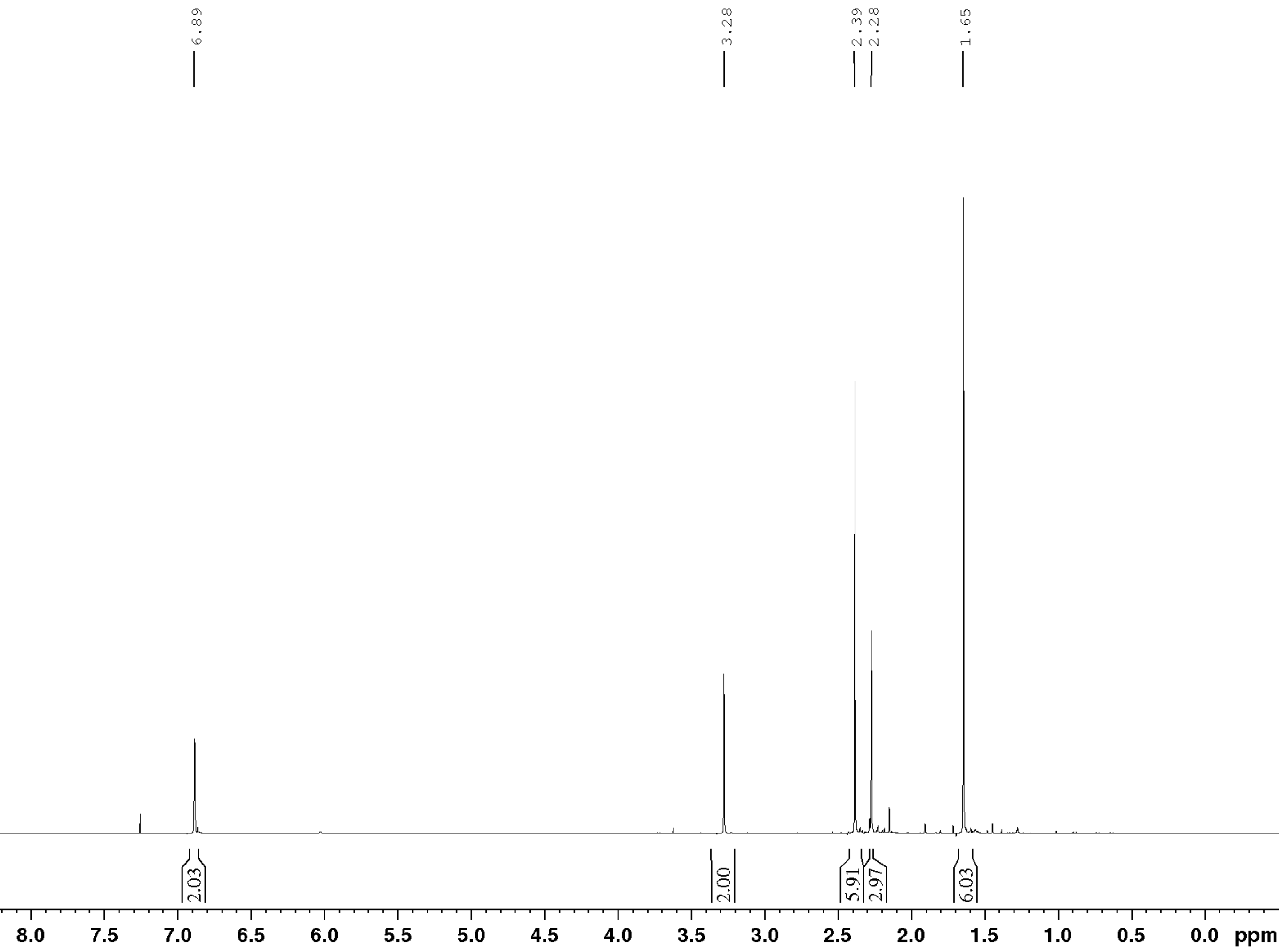

Figure S34. 2-(2-Chloro-2-methylpropyl)-1,3,5-trimethylbenzene (3l) : $^{13}C\{^{1}H\}$ NMR (101 MHz, $CDCl_3$, 298 K)

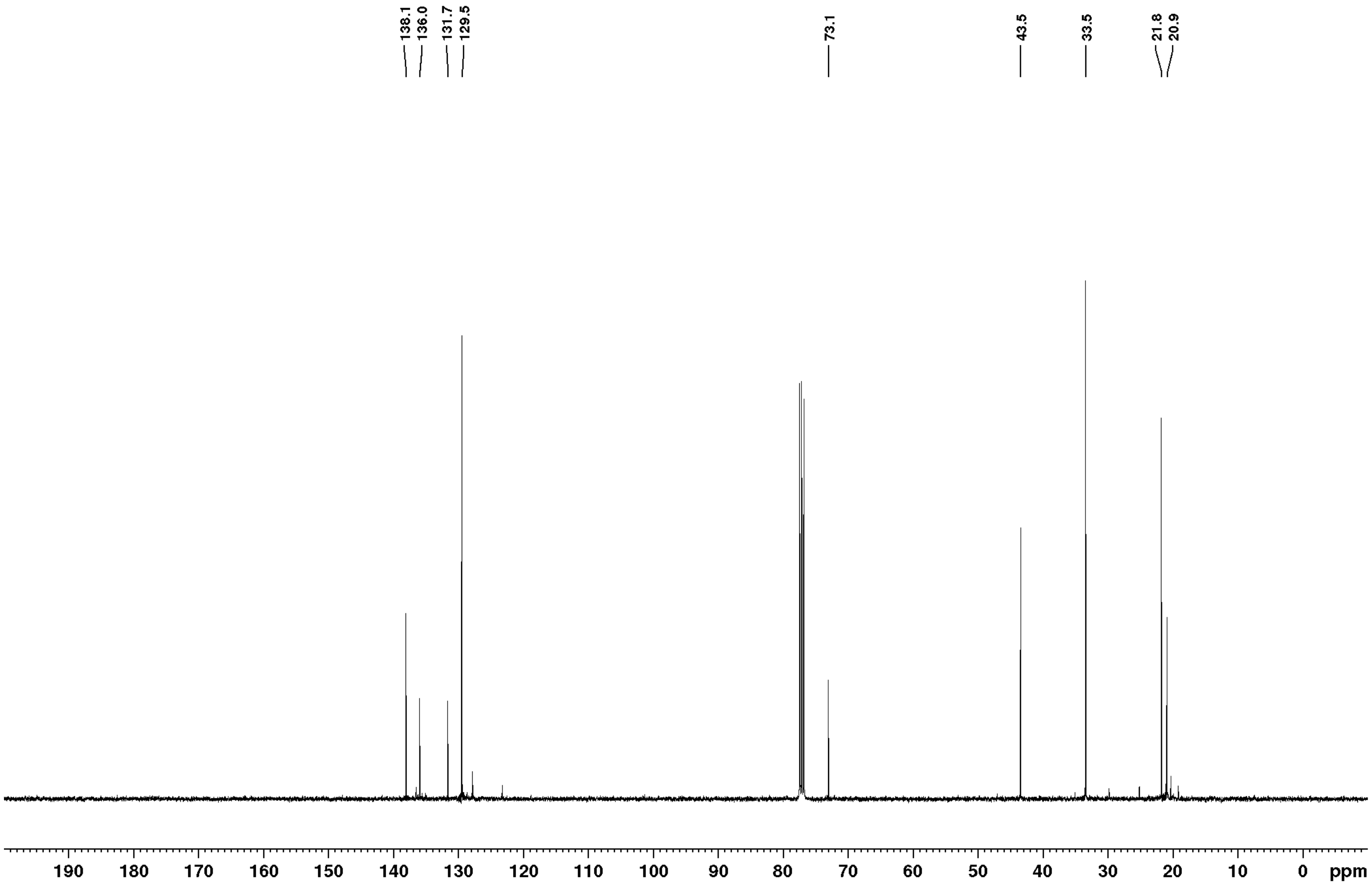

Figure S35. 1-(2-Chloro-2-methylpropyl)naphthalene (3m): ^{1}H NMR spectrum (400 MHz, $CDCl_3$, 298 K)

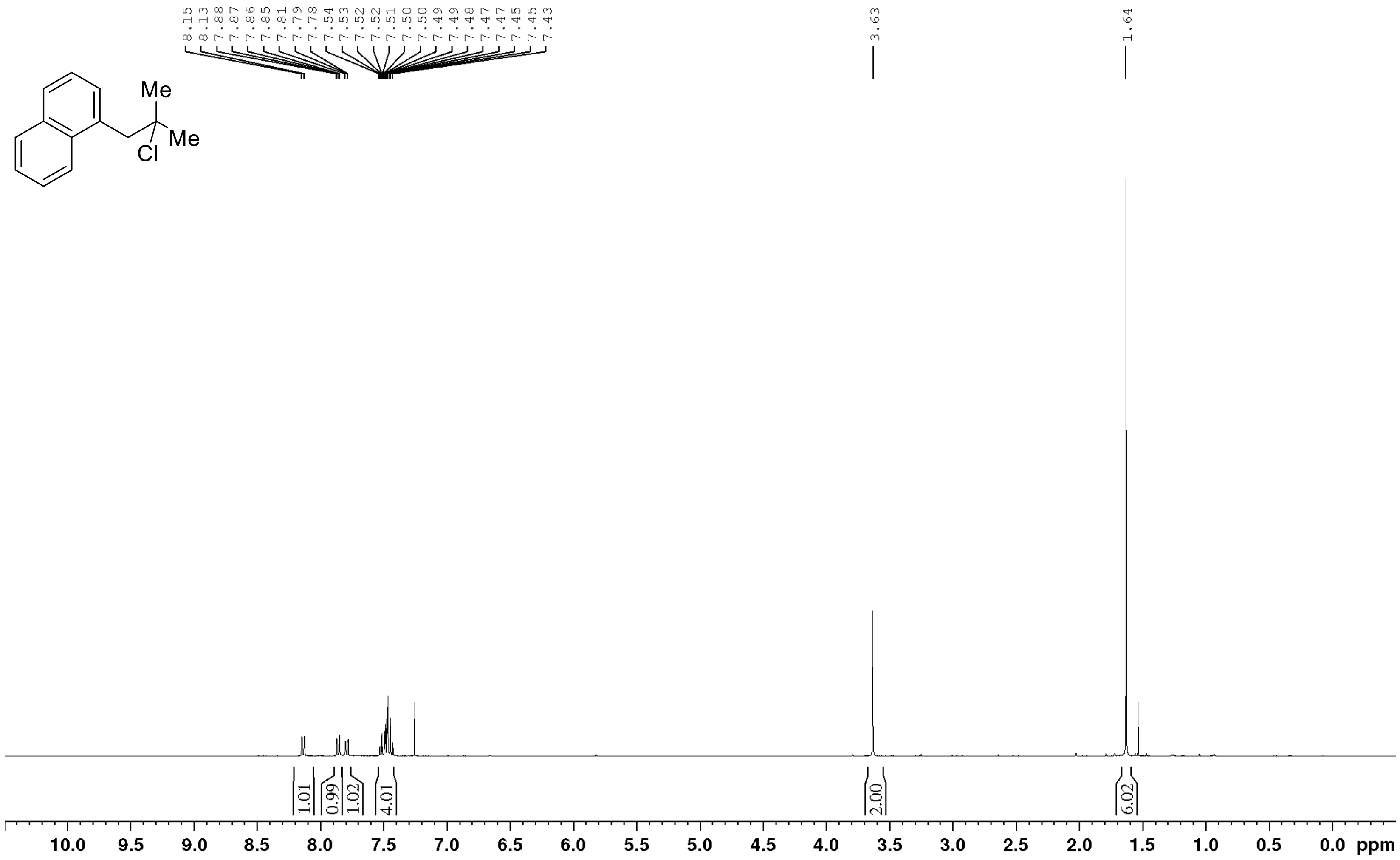

Figure S36. 1-(2-Chloro-2-methylpropyl)naphthalene (3m) : $^{13}C\{^{1}H\}$ NMR (101 MHz, $CDCl_3$, 298 K)

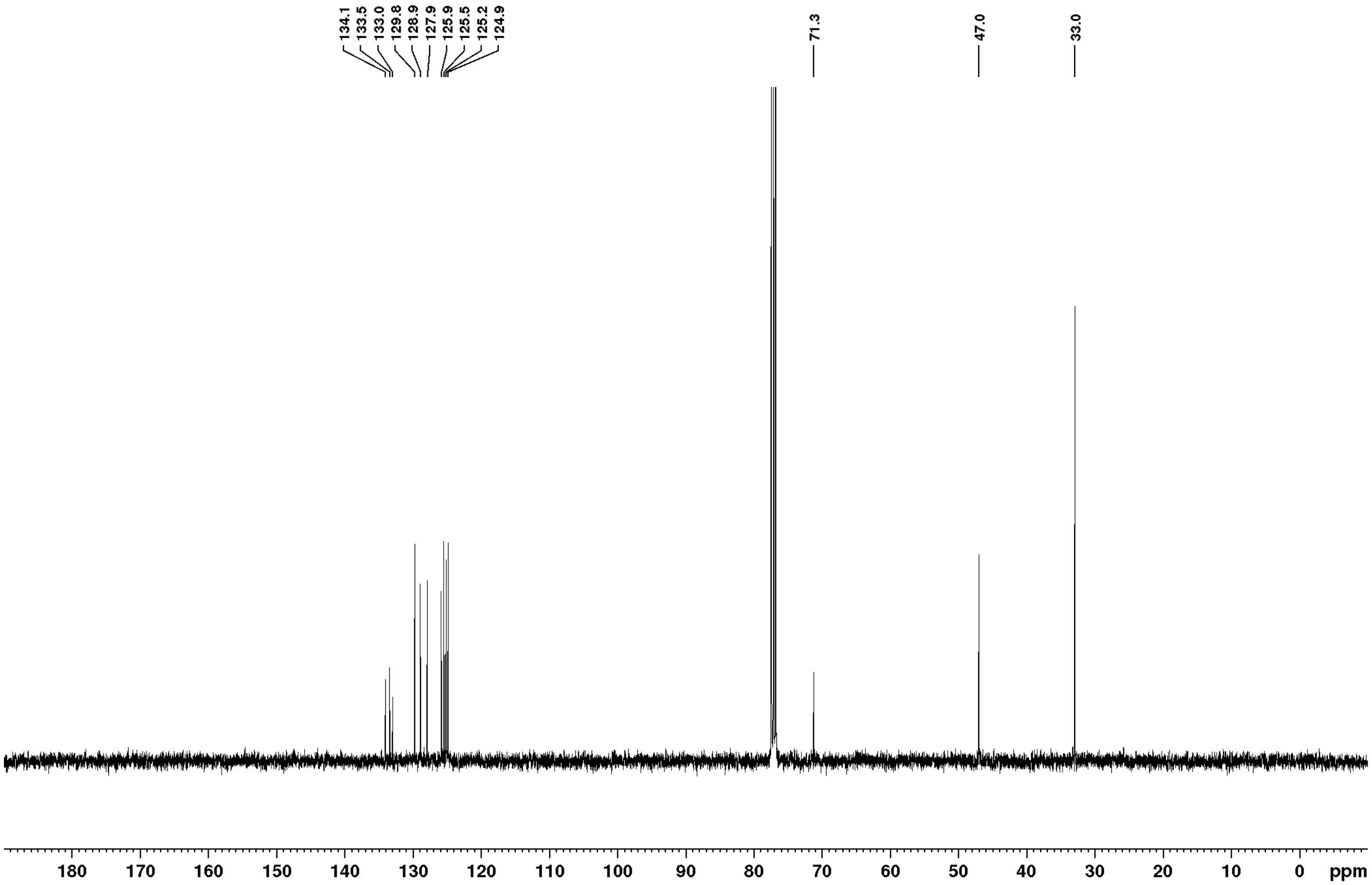

Figure S37. 2-(2-Chloro-2-methylpropyl)thiophene (3n): ^{1}H NMR spectrum (500 MHz, $CDCl_3$, 298 K)

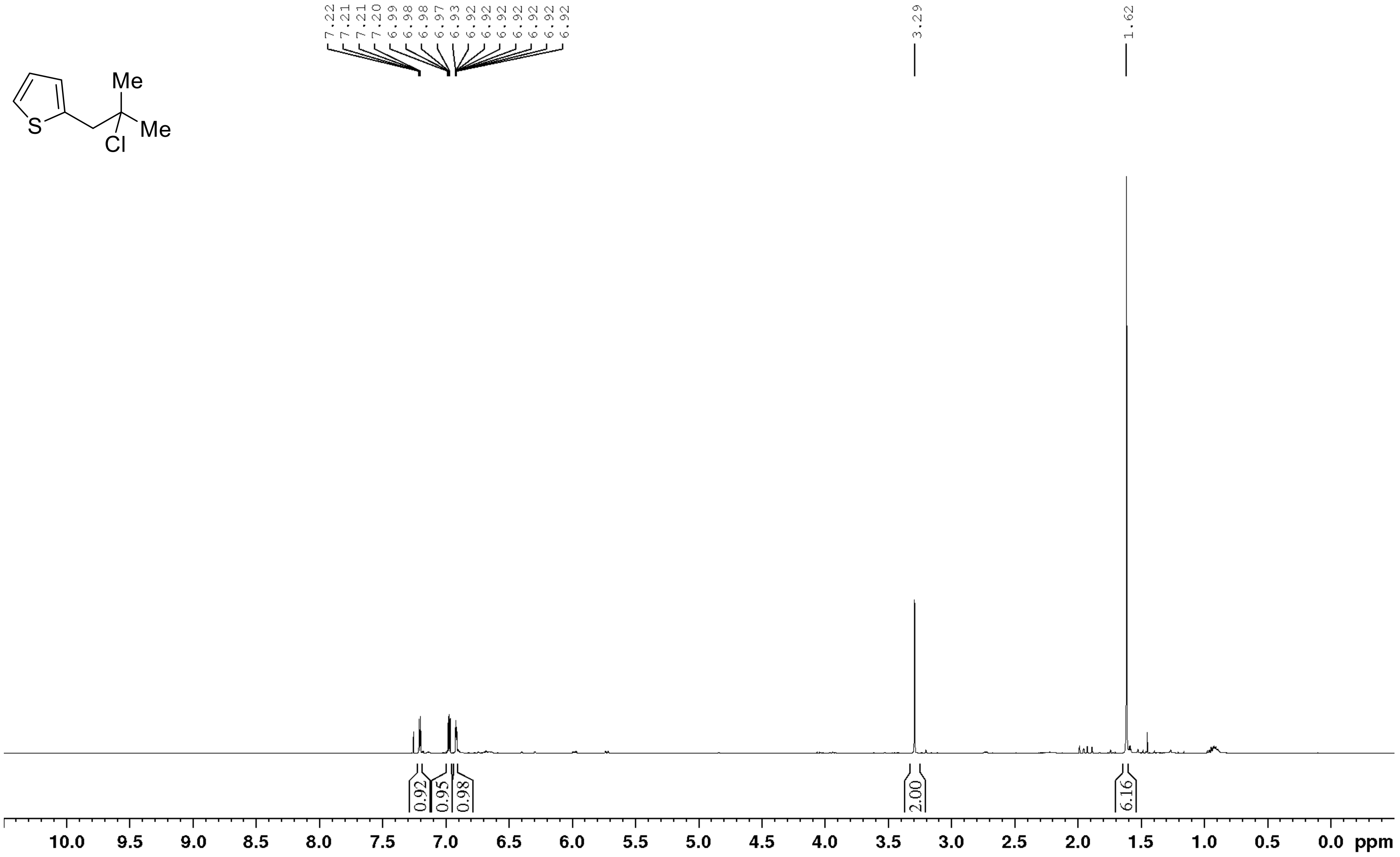

Figure S38. 2-(2-Chloro-2-methylpropyl)thiophene (3n): $^{13}C\{^{1}H\}$ NMR (125 MHz, $CDCl_3$, 298 K)

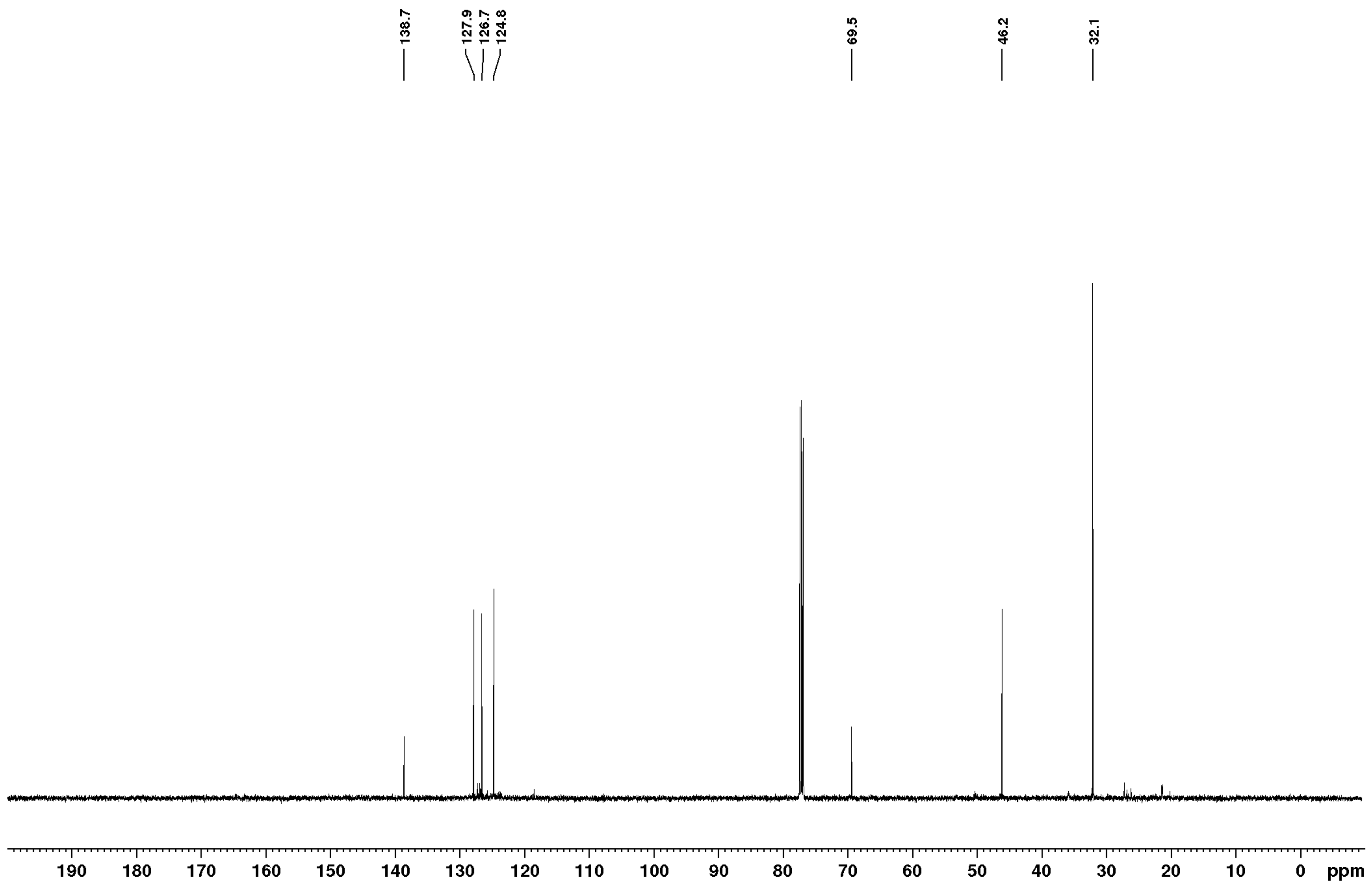

Figure S39. (3-Chloro-3-methylbutyl)benzene (3o): ^{1}H NMR spectrum (400 MHz, $CDCl_3$, 298 K)

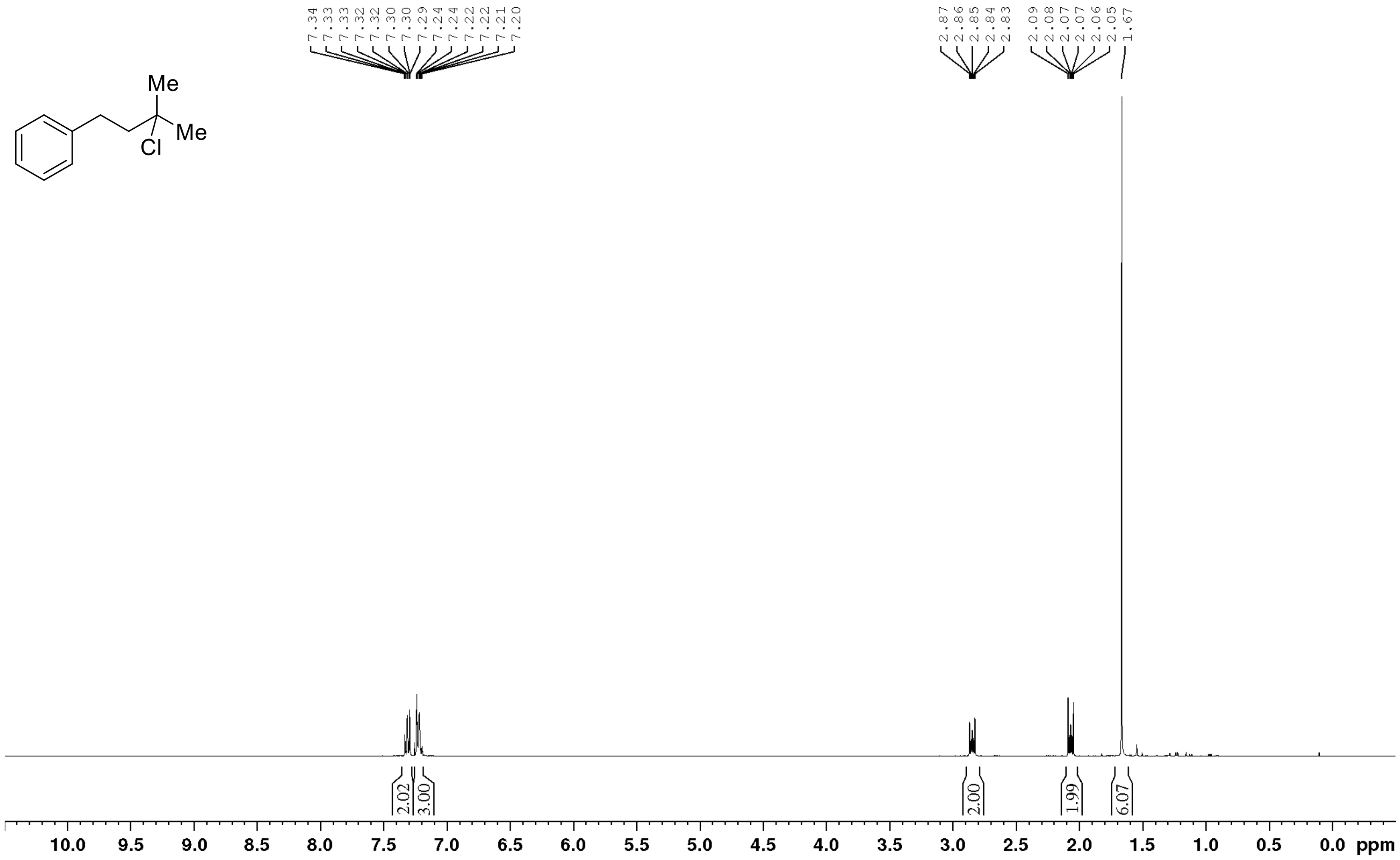

Figure S40. (3-Chloro-3-methylbutyl)benzene (3o): $^{13}C\{^{1}H\}$ NMR (101 MHz, $CDCl_3$, 298 K)

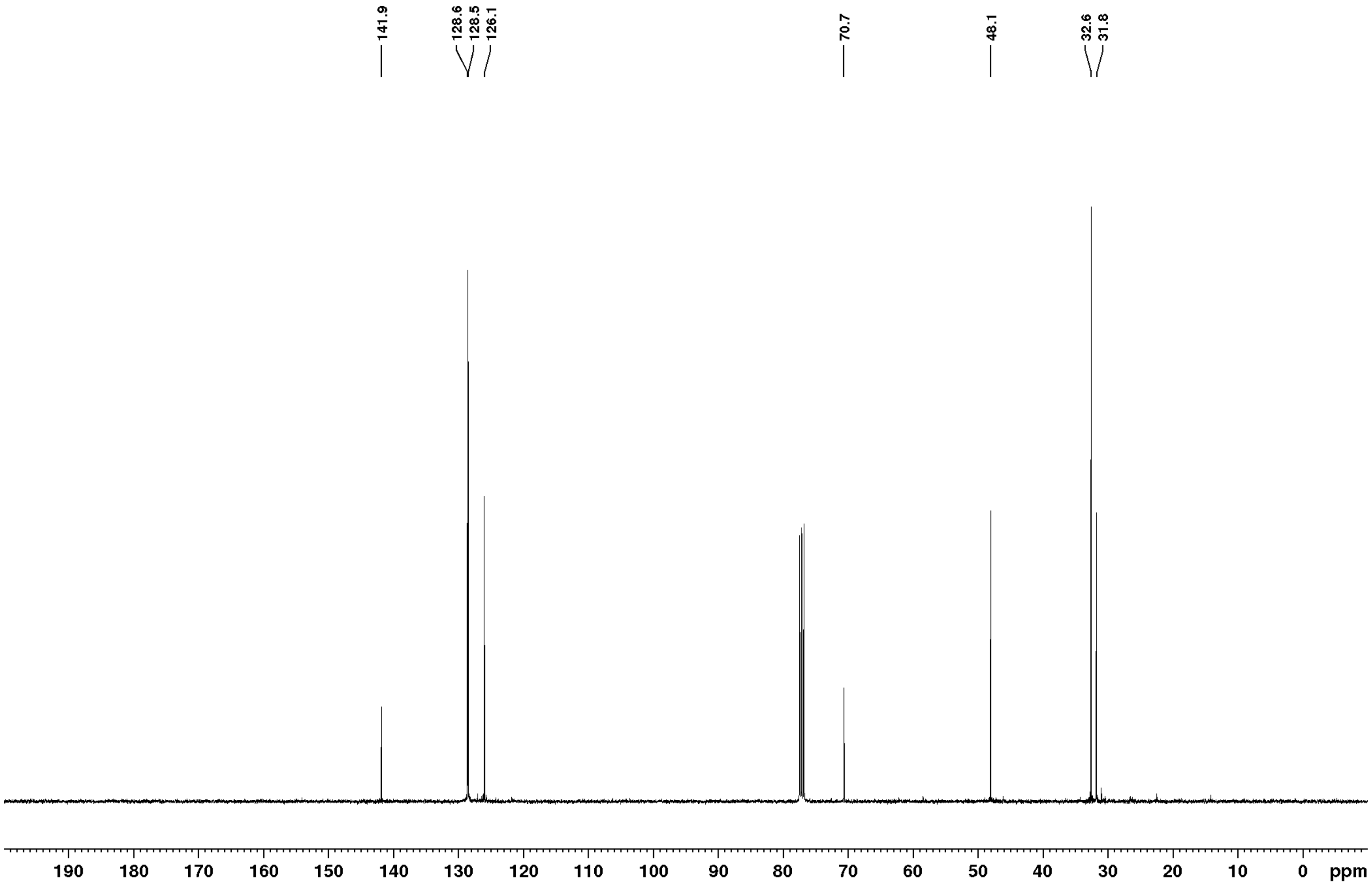

Figure S41. 2-Chloro-2-methylnonane (3p): ^{1}H NMR spectrum (400 MHz, $CDCl_3$, 298 K)

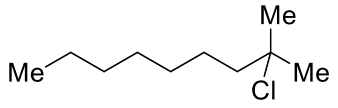

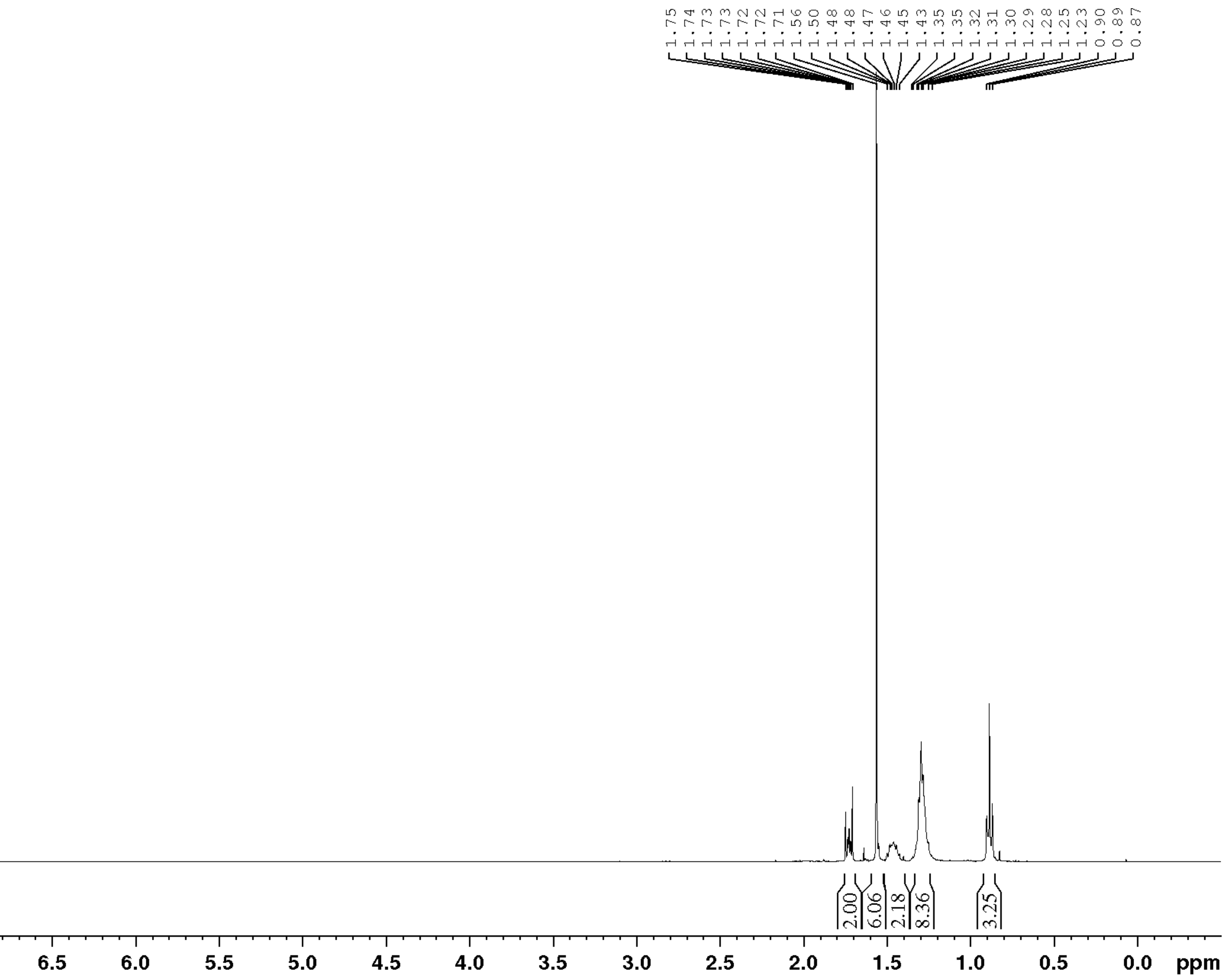

Figure S42. 2-Chloro-2-methylnonane (3p): $^{13}C\{^{1}H\}$ NMR (101 MHz, $CDCl_3$, 298 K)

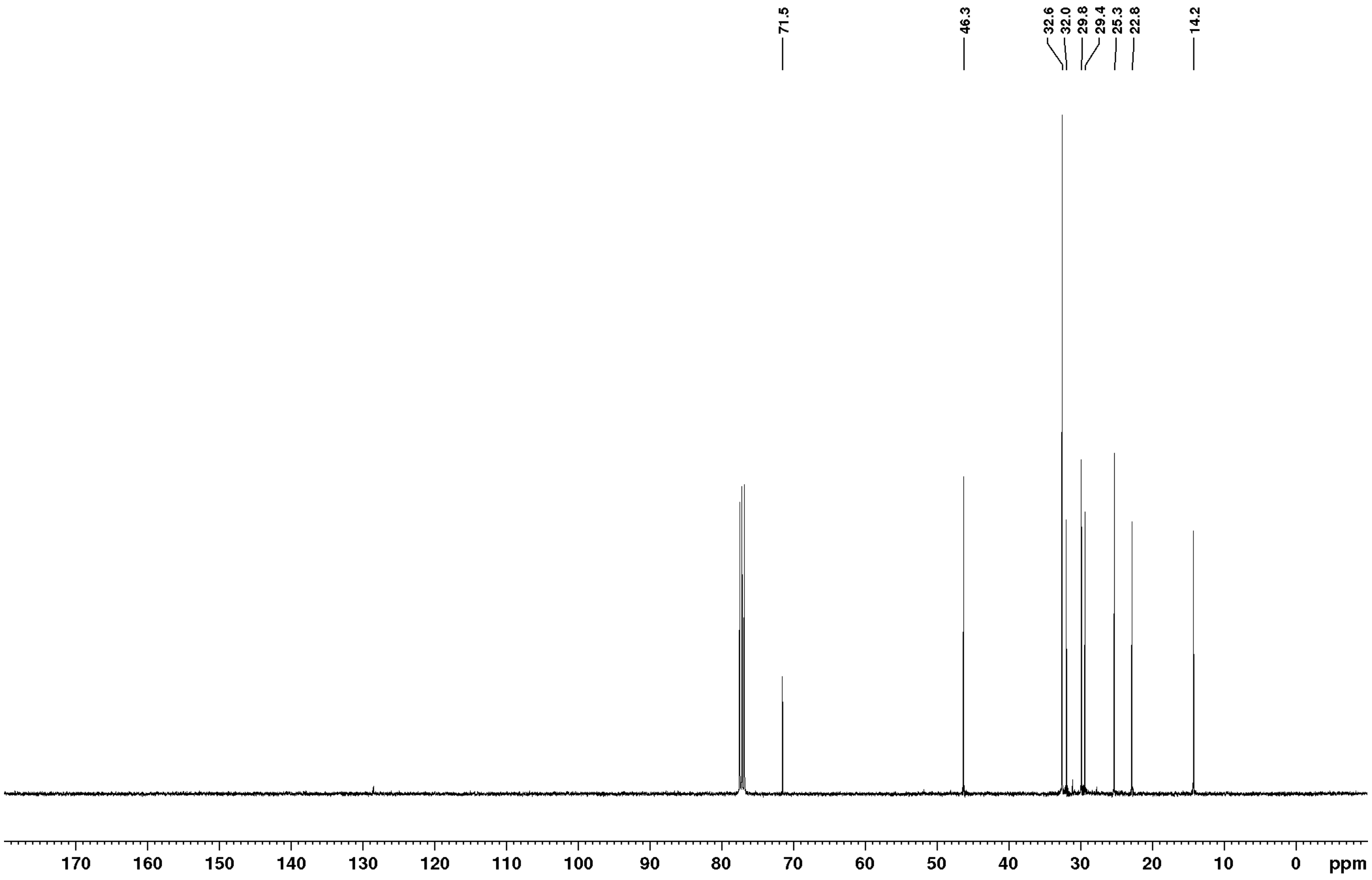

Figure S43. (2-Chloro-2-methylpropane-1,3-diyl)dibenzene (3q): ^{1}H NMR spectrum (400 MHz, $CDCl_3$, 298 K)

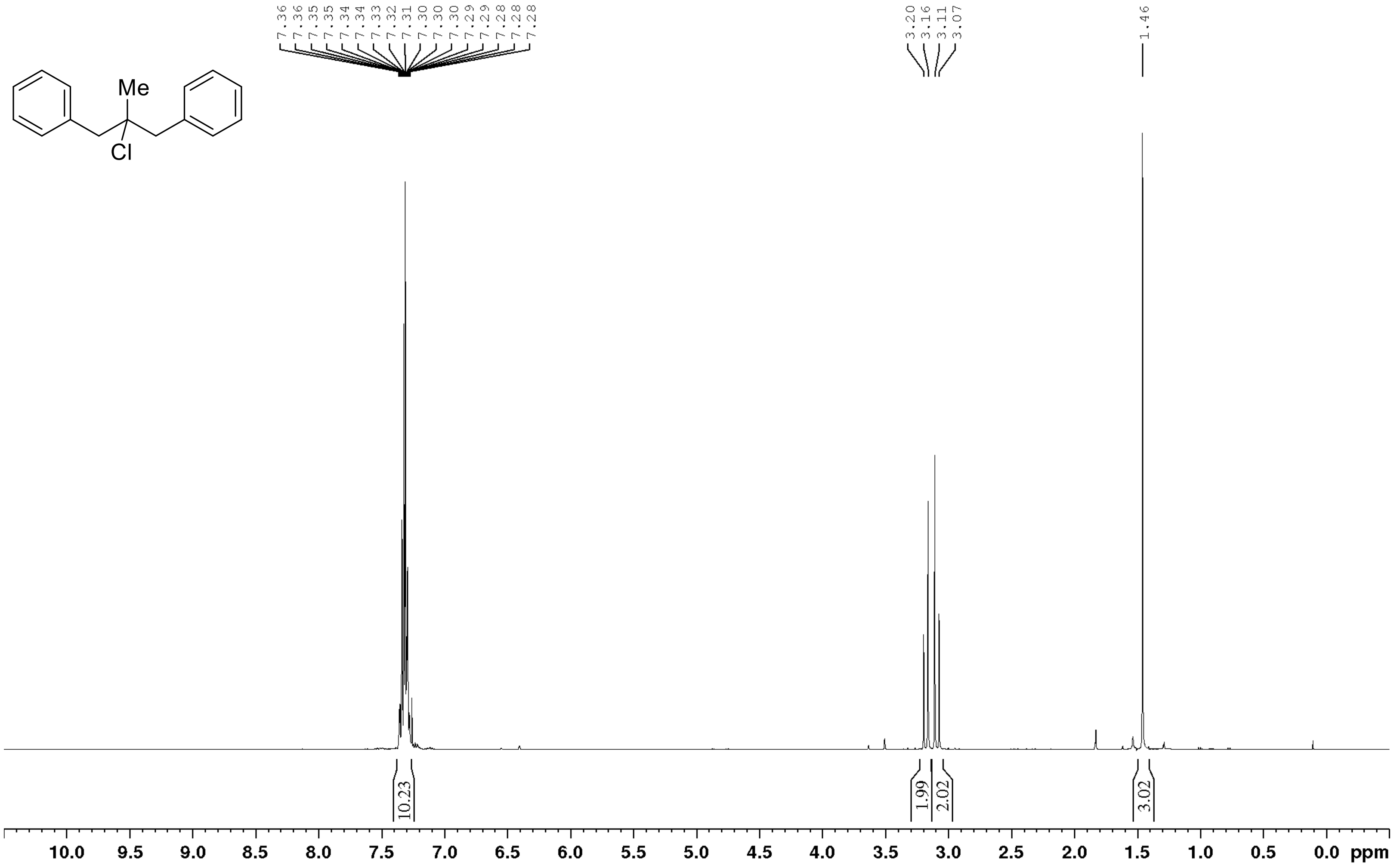

Figure S44. (2-Chloro-2-methylpropane-1,3-diyl)dibenzene (3q): ^{1}H NMR spectrum (400 MHz, $CDCl_3$, 298 K)

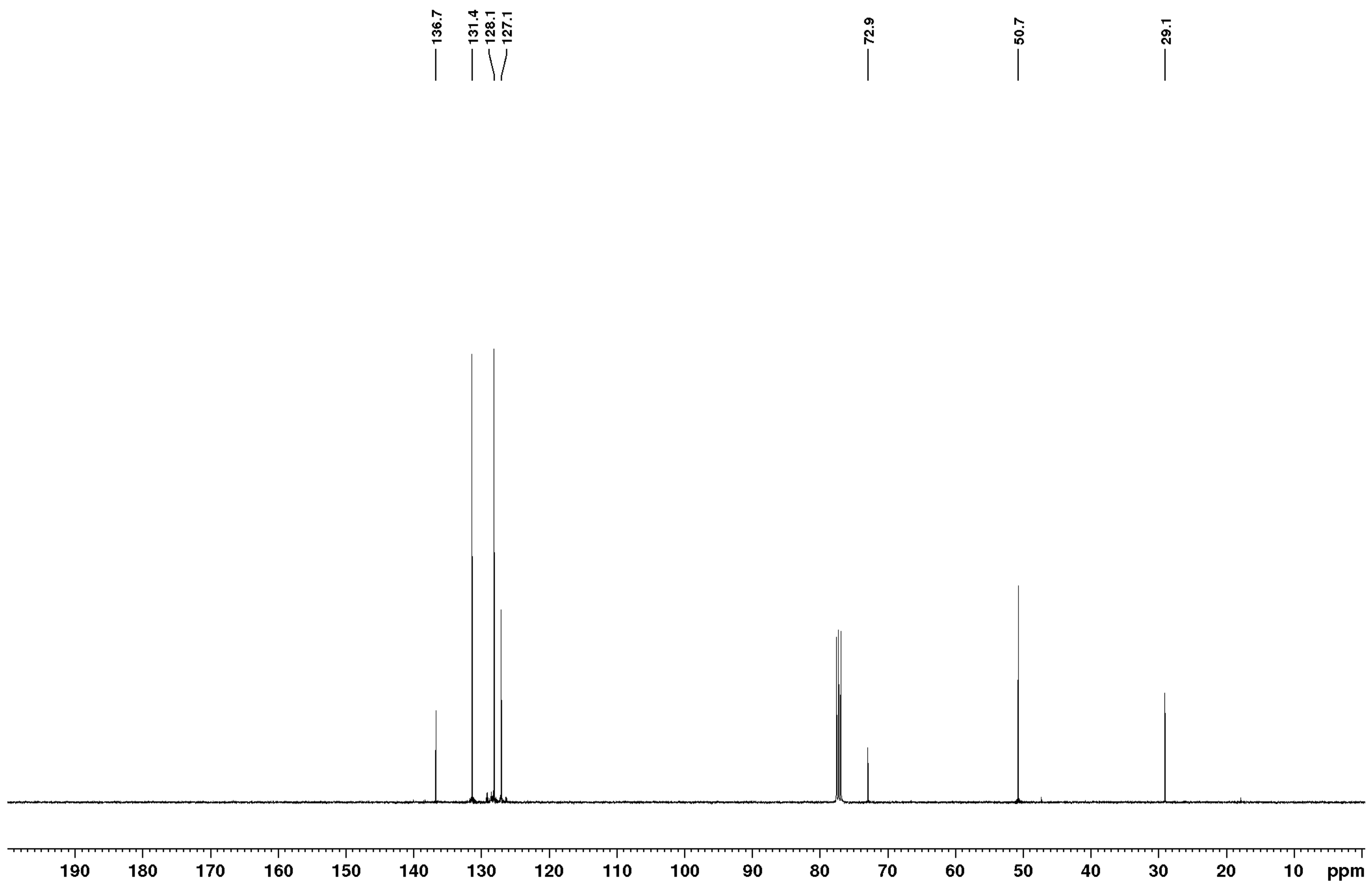

Figure S45. 1-Chloro-1-methylcyclooctane (3r): ^{1}H NMR spectrum (400 MHz, $CDCl_3$, 298 K)

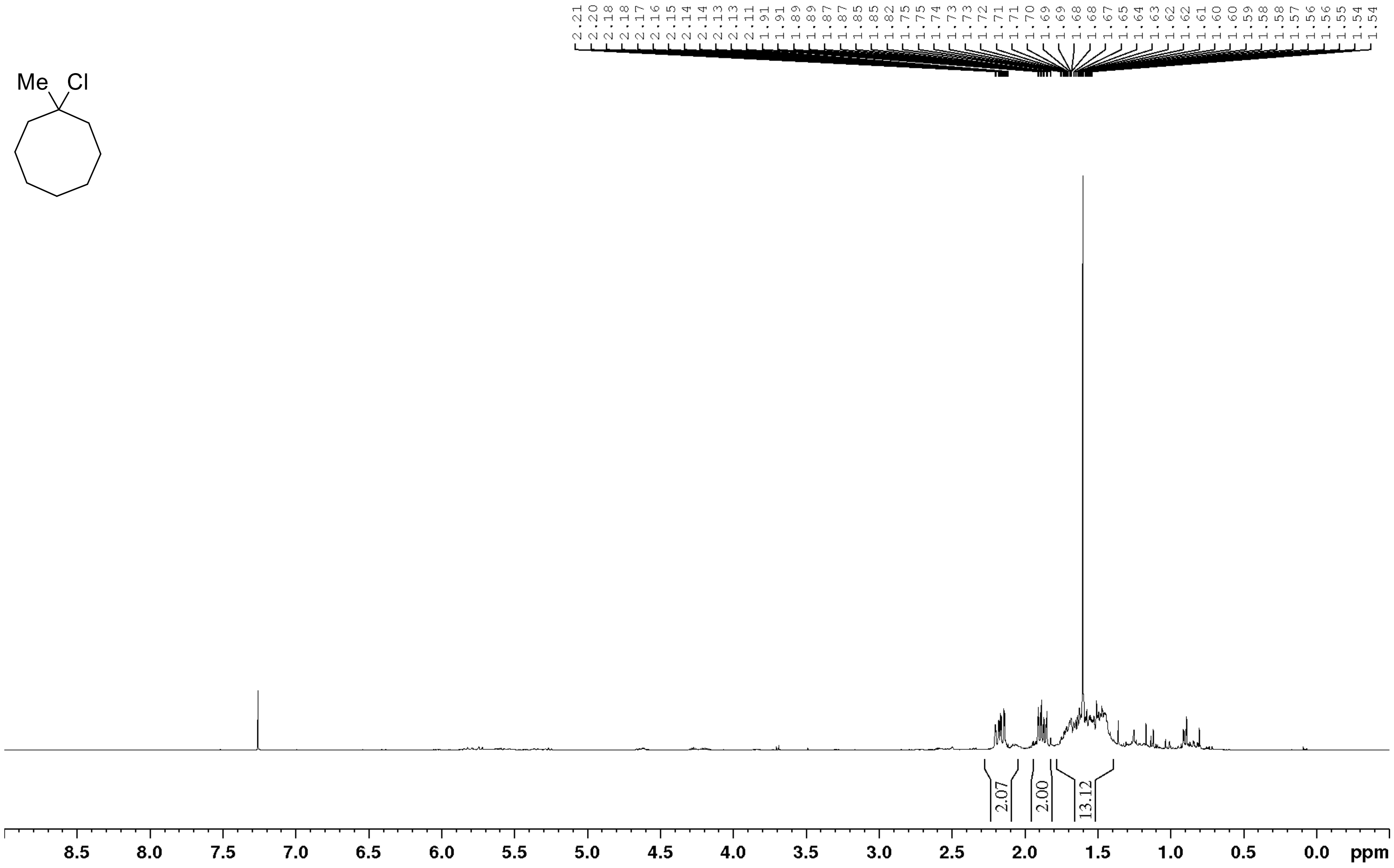

Figure S46. 1-Chloro-1-methylcyclooctane (3r): $^{13}C\{^{1}H\}$ NMR (101 MHz, $CDCl_3$, 298 K)

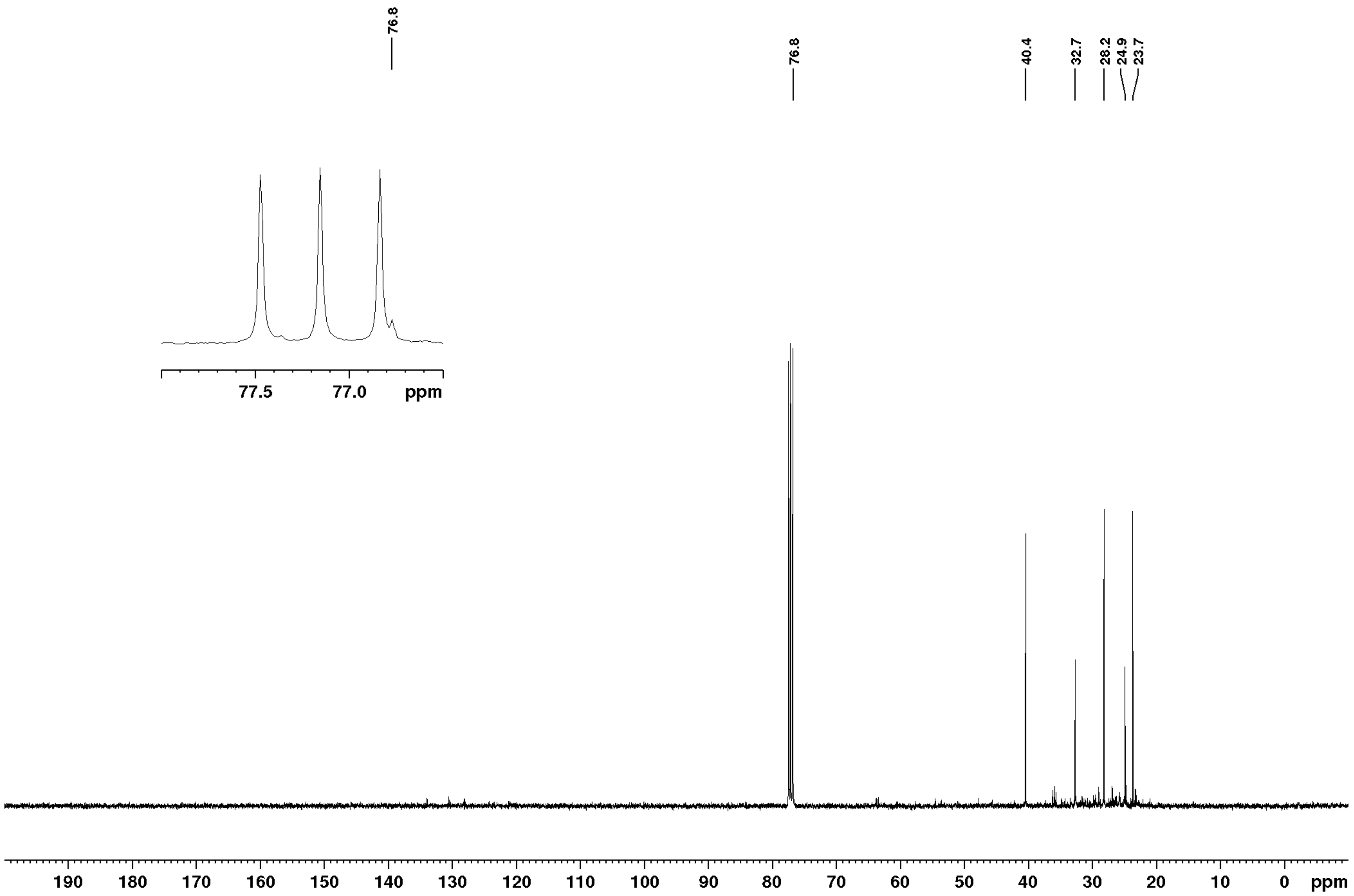

Figure S47. ***tert*-Butyl(3-chloro-3-methylbutoxy)diphenylsilane (3s):** ^{1}H NMR spectrum (400 MHz, $CDCl_3$, 298 K)

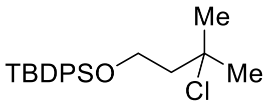

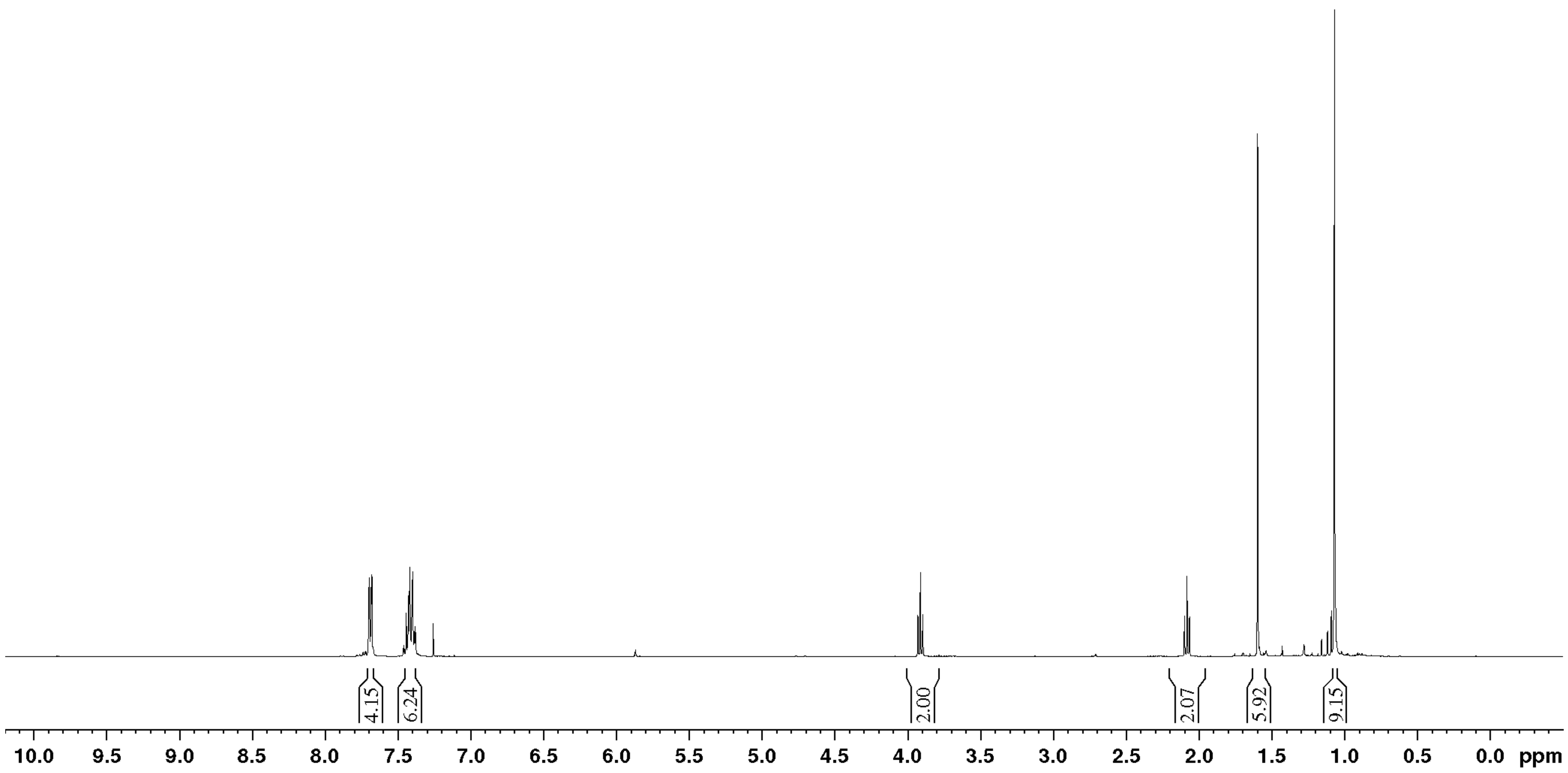

Figure S48. *tert*-Butyl(3-chloro-3-methylbutoxy)diphenylsilane (3s): $^{13}C\{^1H\}$ NMR (101 MHz, $CDCl_3$, 298 K)

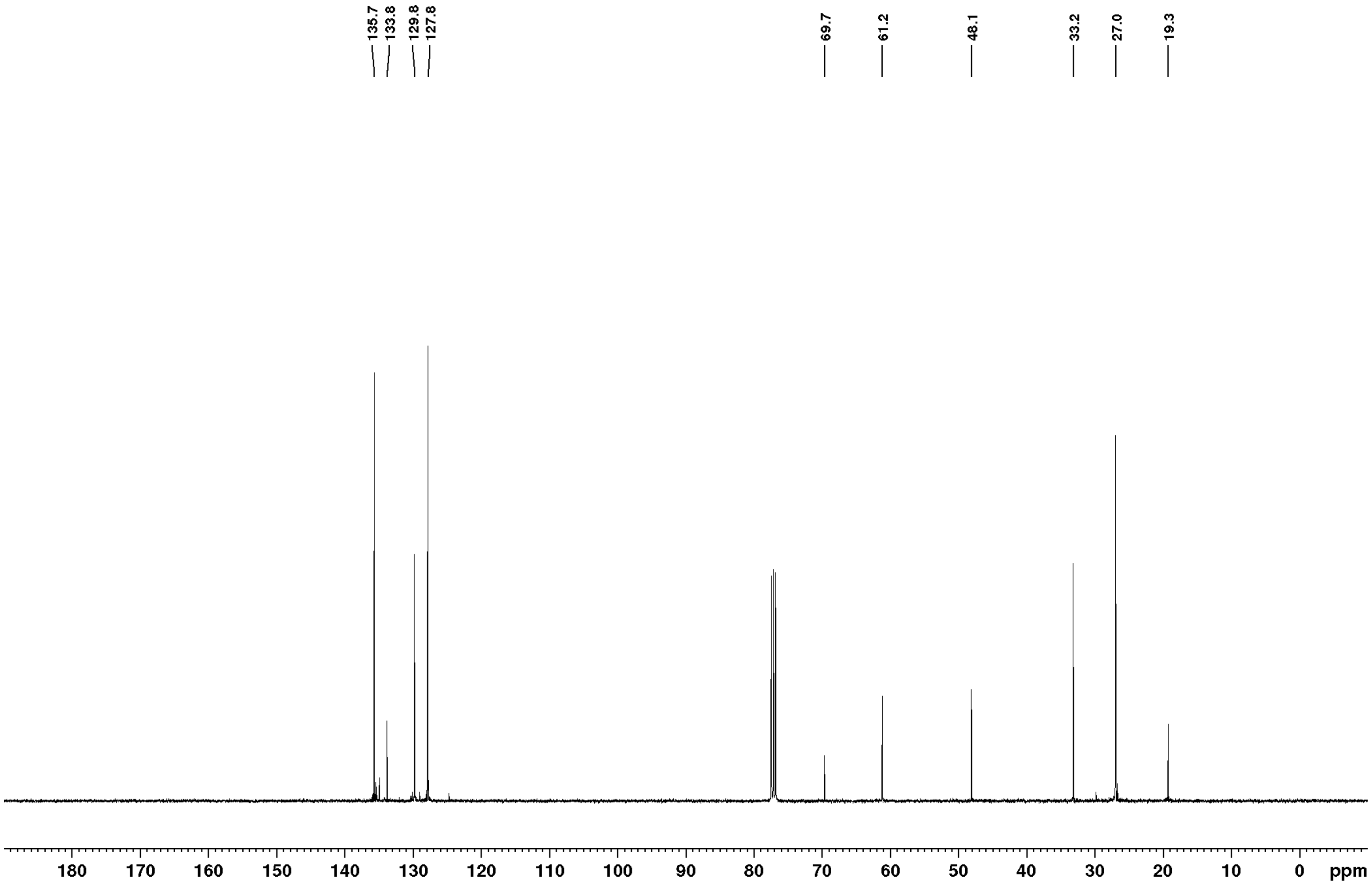

Figure S49. ((3-Chloro-3-methylbutoxy)methyl)benzene (3t): ^{1}H NMR spectrum (400 MHz, $CDCl_3$, 298 K)

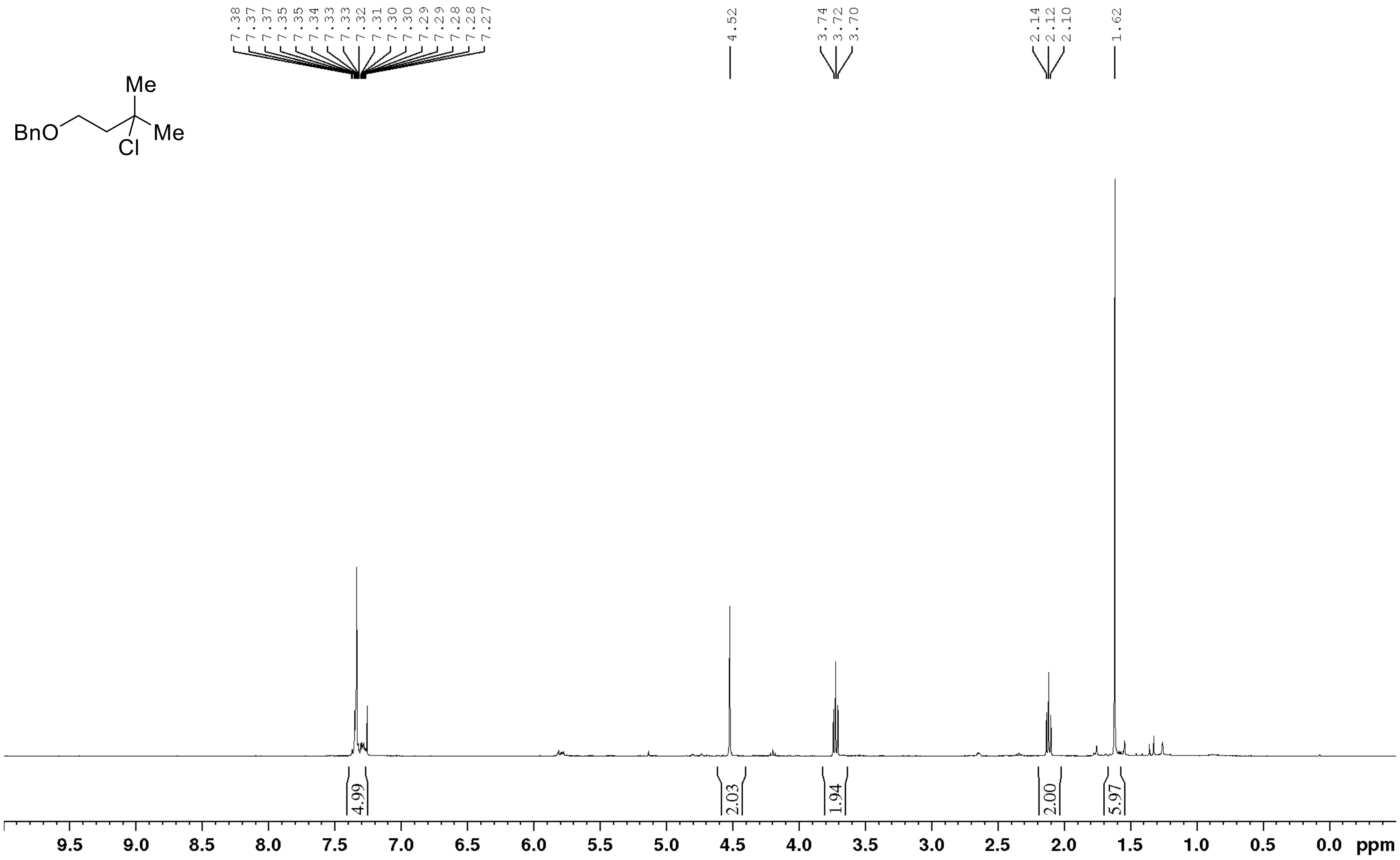

Figure S50. ((3-Chloro-3-methylbutoxy)methyl)benzene (3t): $^{13}C\{^{1}H\}$ NMR (101 MHz, $CDCl_3$, 298 K)

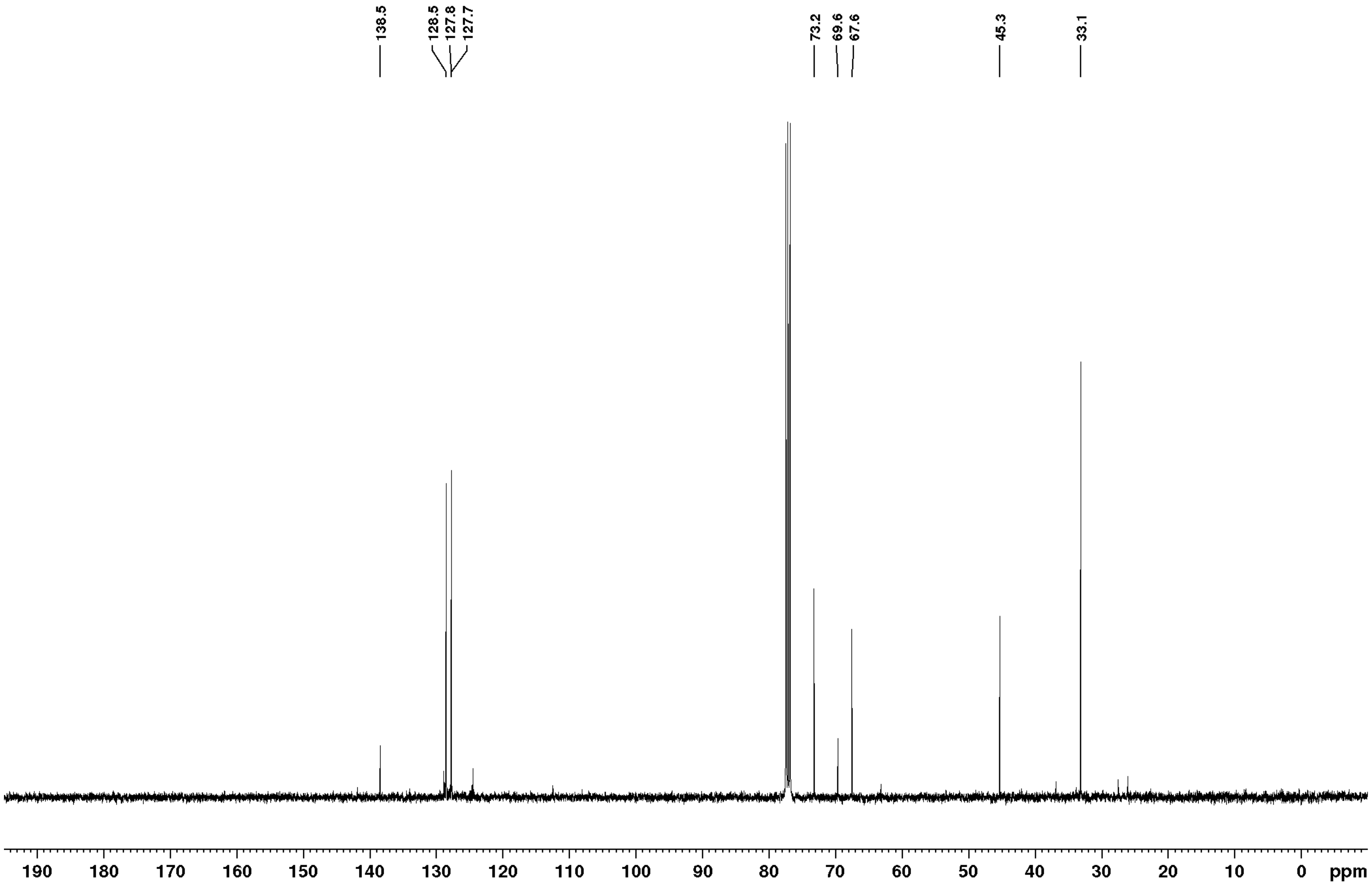

Figure S51. 3-Chloro-3-methylbutyl pivalate (3u): ^{1}H NMR spectrum (400 MHz, $CDCl_3$, 298 K)

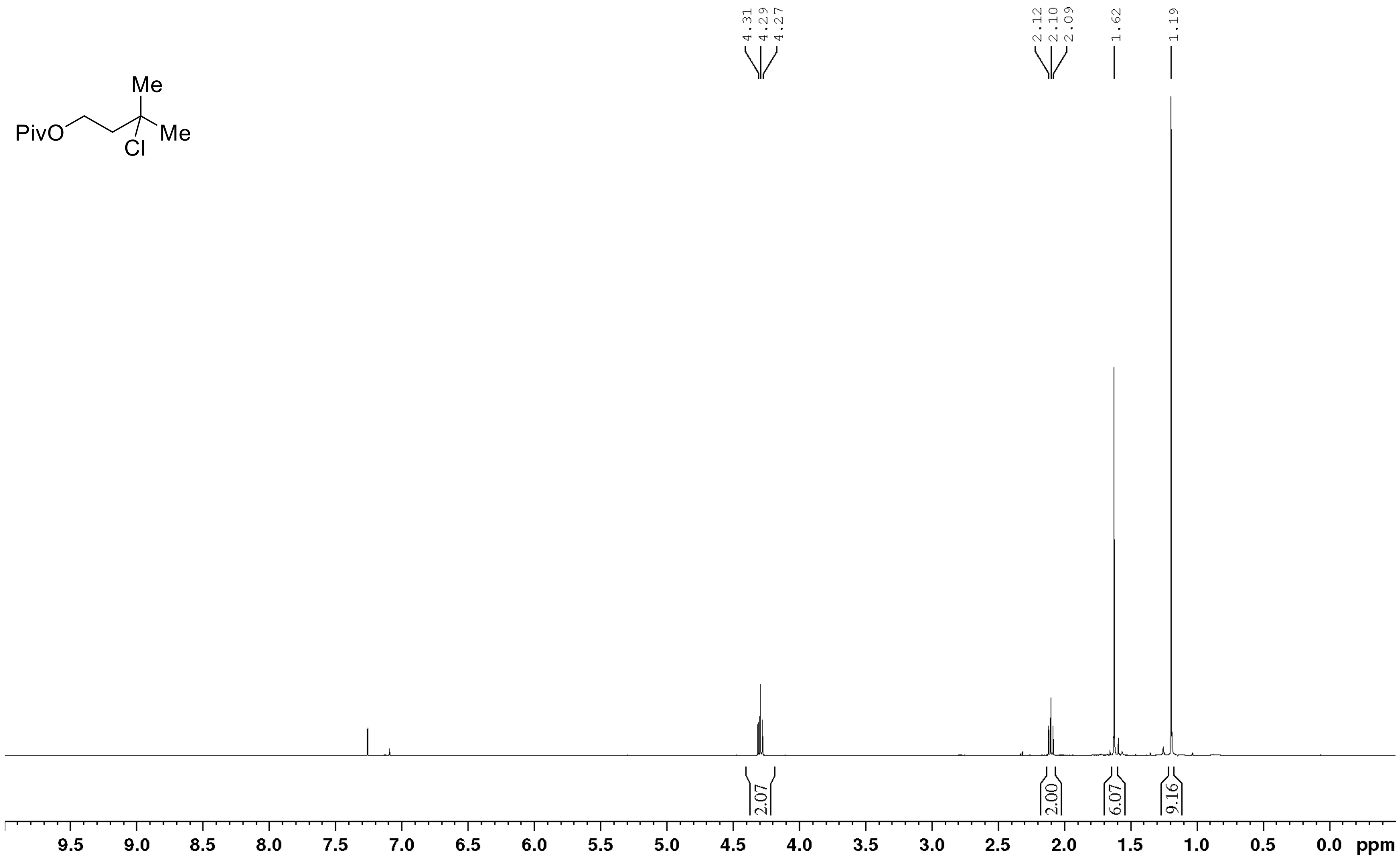

Figure S52. 3-Chloro-3-methylbutyl pivalate (3u): $^{13}C\{^{1}H\}$ NMR (101 MHz, $CDCl_3$, 298 K)

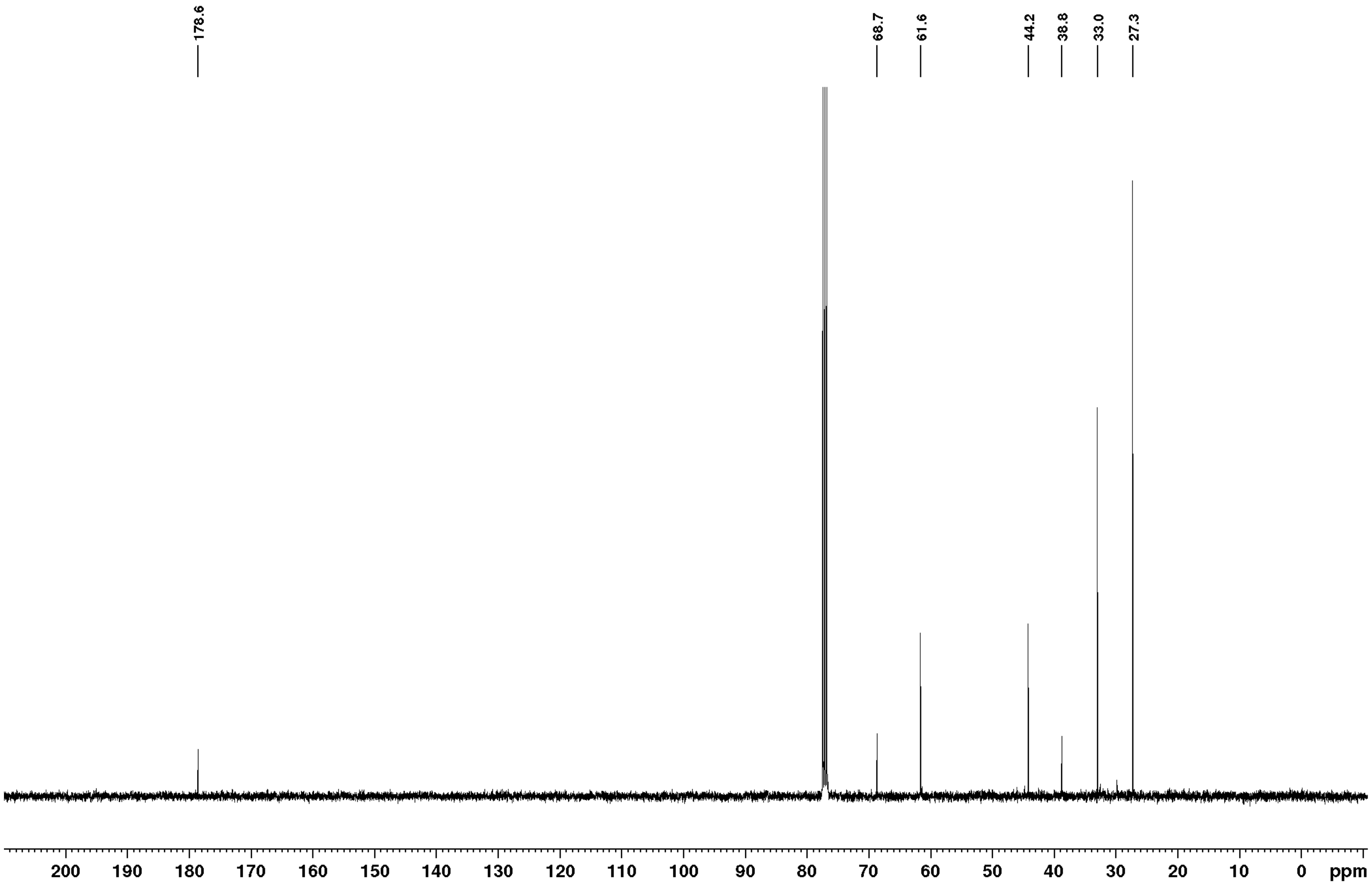

Figure S53. 2-(4-Chloro-4-methylpentyl)isoindoline-1,3-dione (3x): ^{1}H NMR spectrum (400 MHz, $CDCl_3$, 298 K)

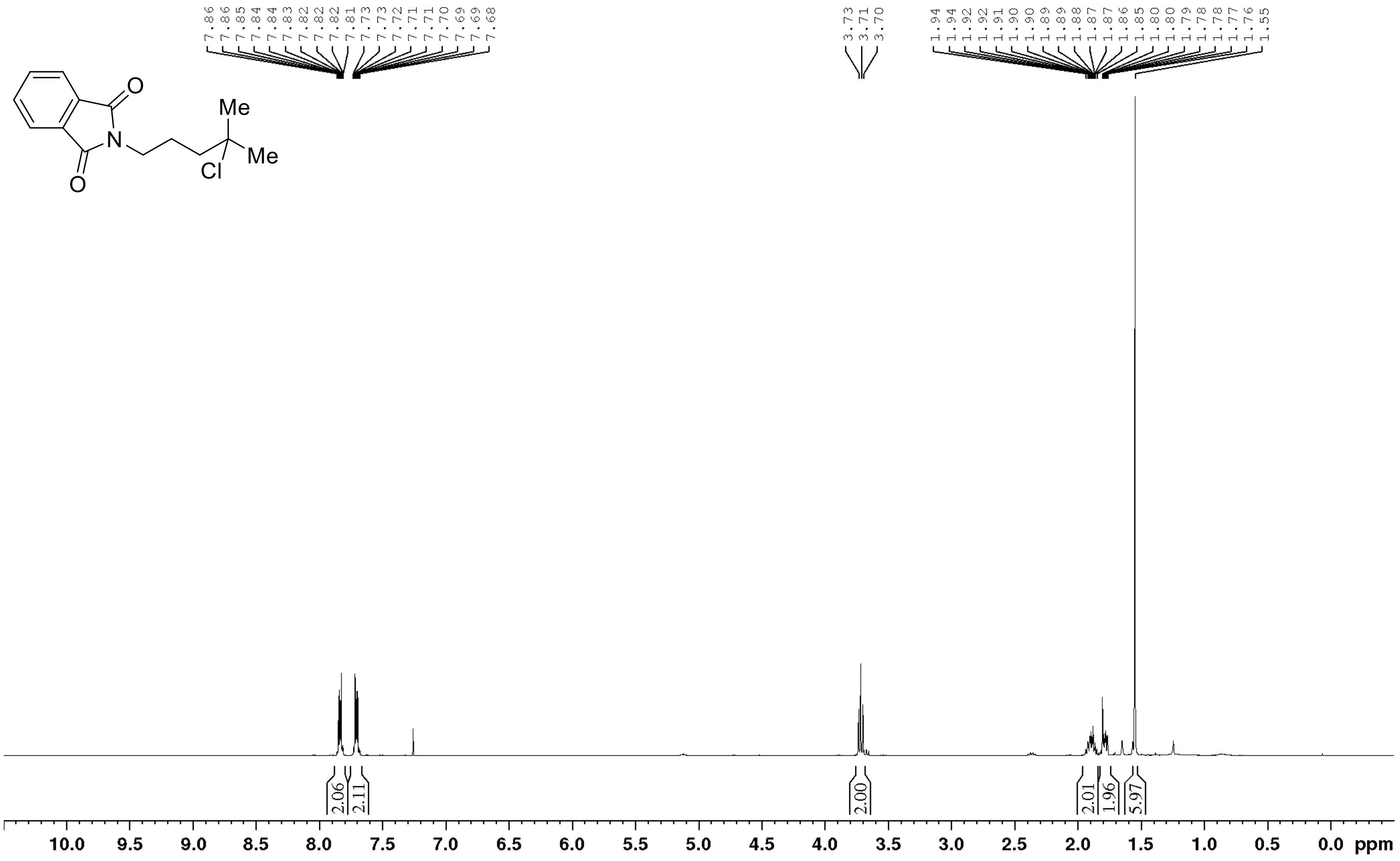

Figure S54. 2-(4-Chloro-4-methylpentyl)isoindoline-1,3-dione (3x): $^{13}C\{^{1}H\}$ NMR (101 MHz, $CDCl_3$, 298 K)

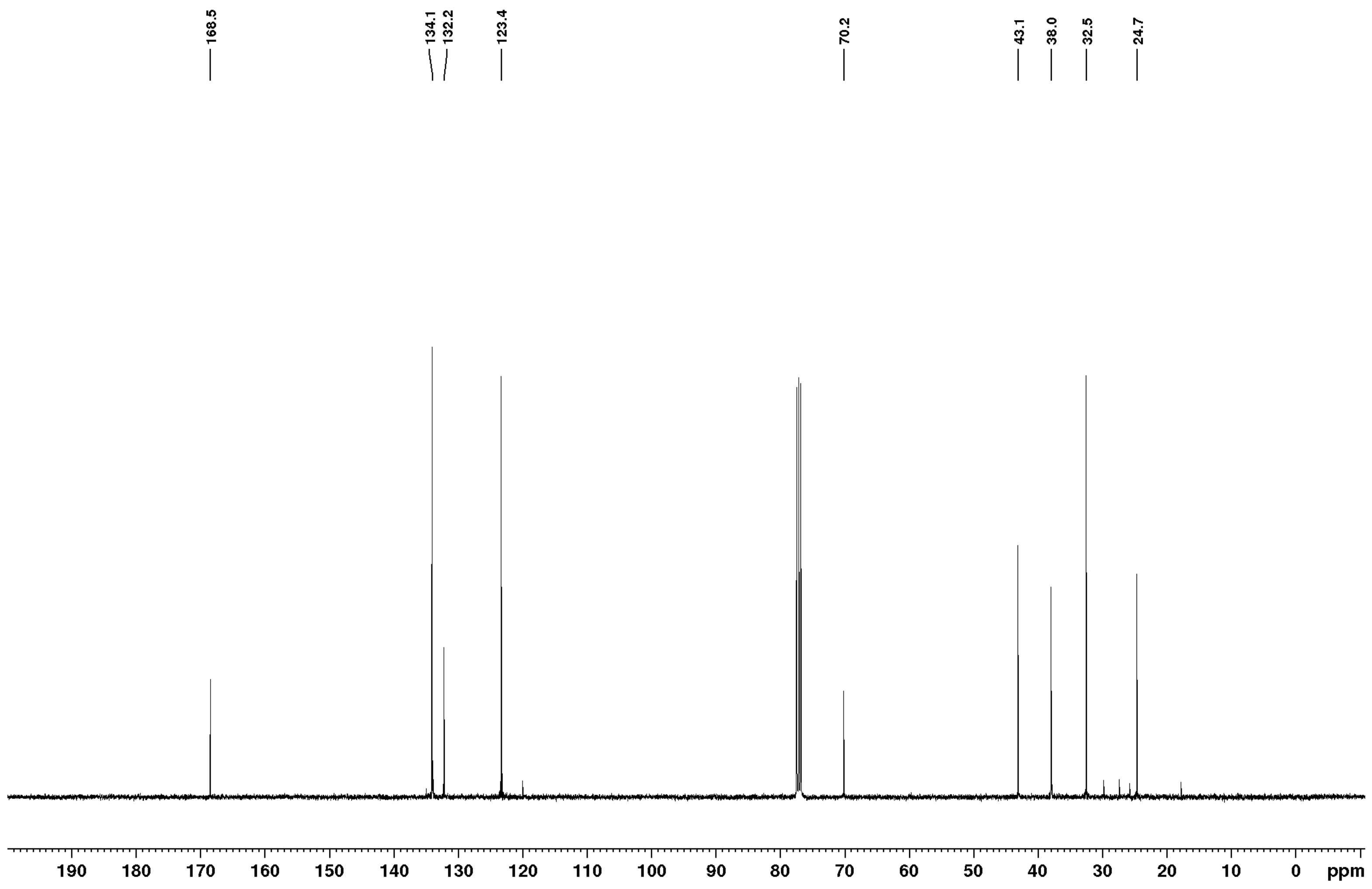

Figure S55. 1-(2-Chloropropyl)naphthalene (3y): ^{1}H NMR spectrum (400 MHz, $CDCl_3$, 298 K)

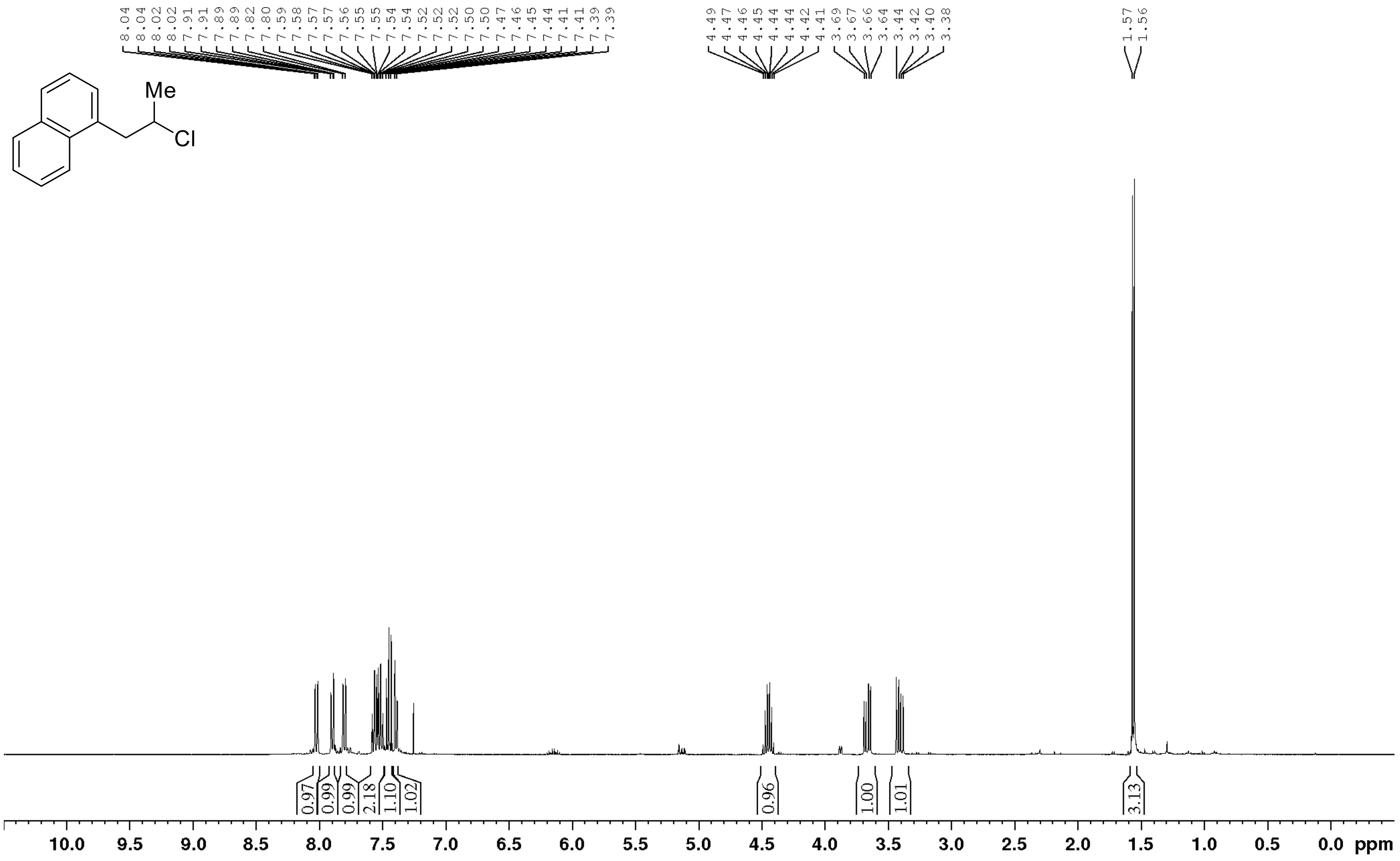

Figure S56. 1-(2-Chloropropyl)naphthalene (3y): $^{13}C\{^{1}H\}$ NMR (101 MHz, $CDCl_3$, 298 K)

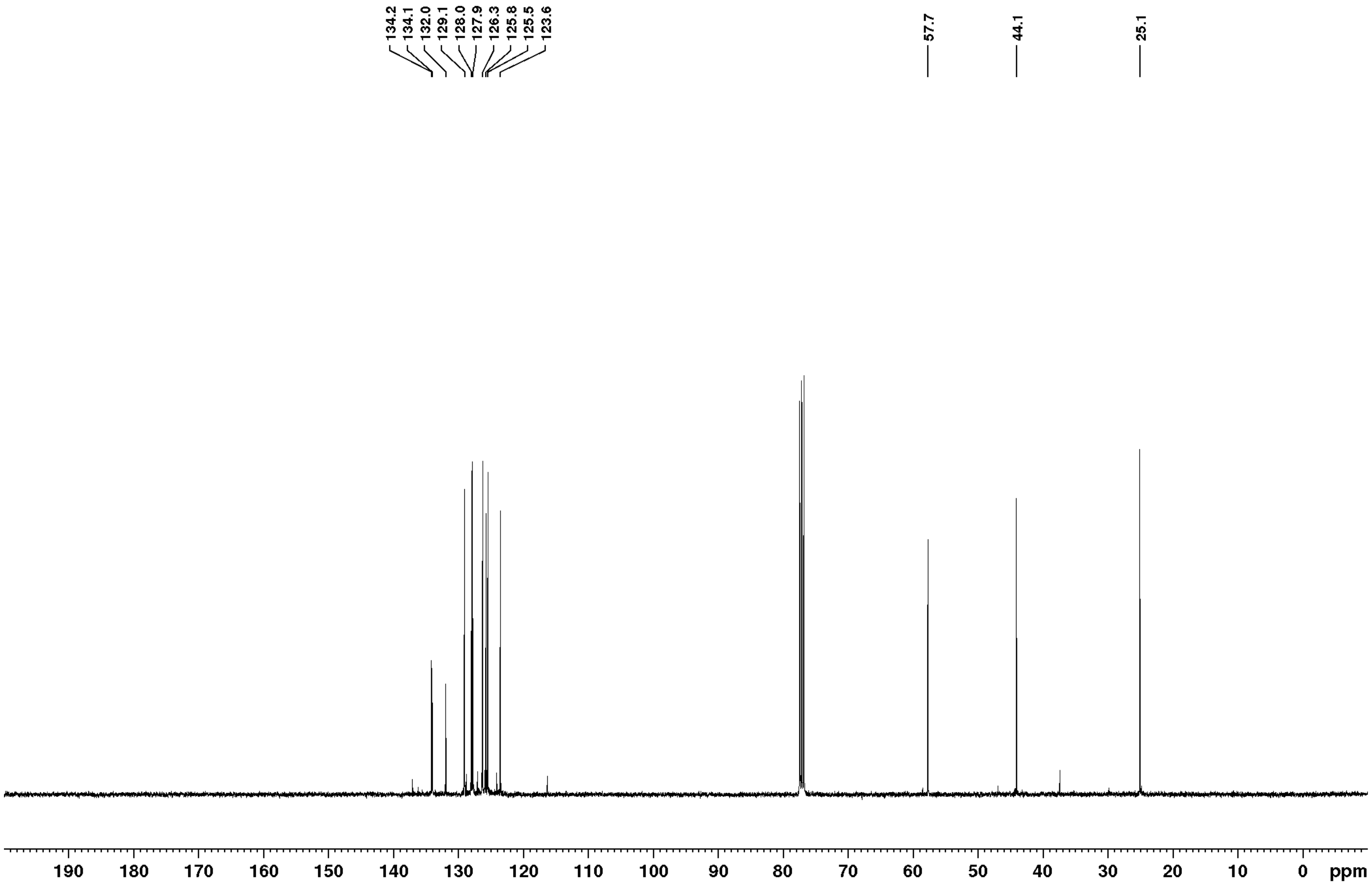

Figure S57. 1-Chloro-3-(1-chloroethyl)benzene (3z): ^{1}H NMR spectrum (400 MHz, $CDCl_3$, 298 K)

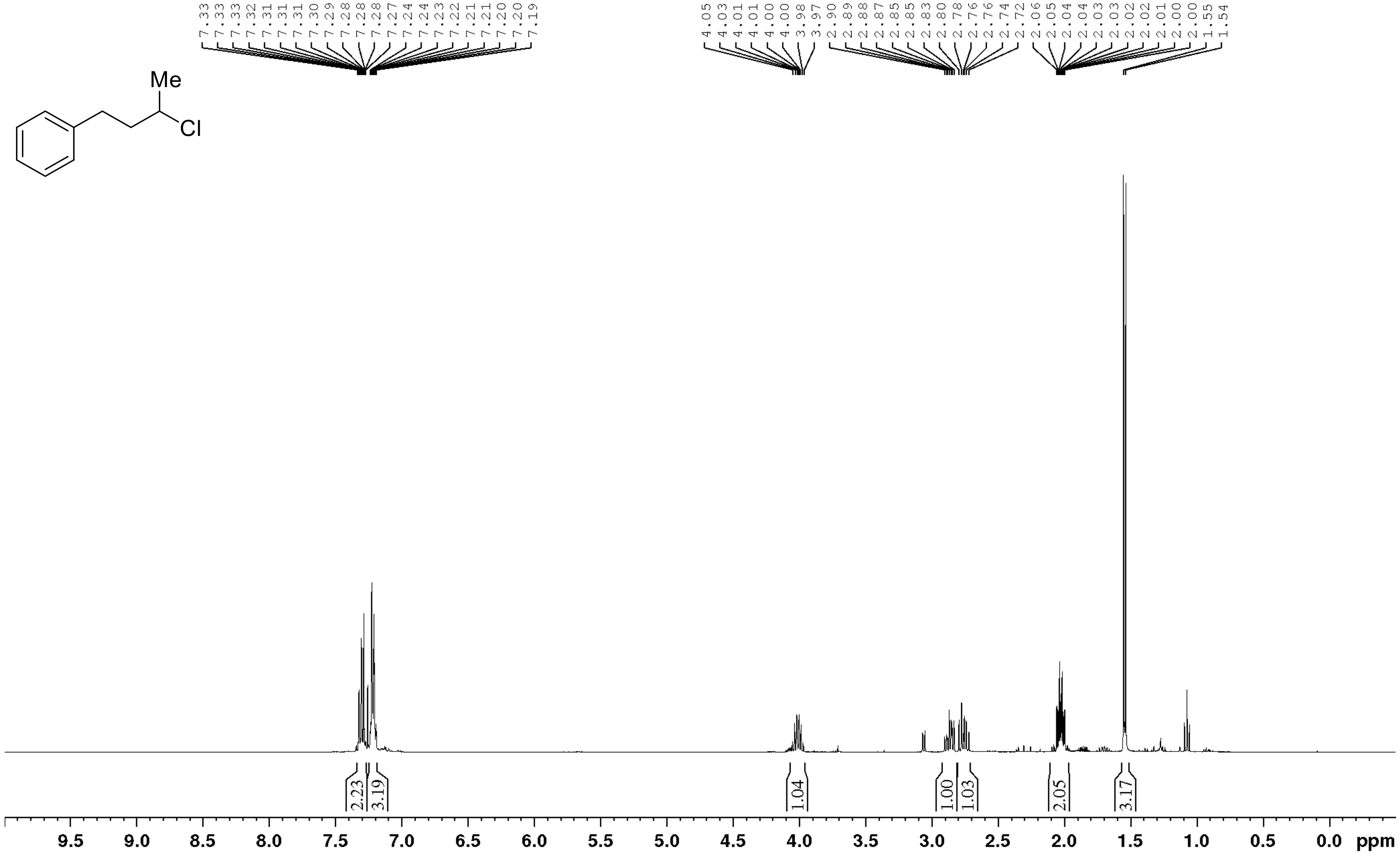

Figure S58. 1-Chloro-3-(1-chloroethyl)benzene (3z): $^{13}C\{^{1}H\}$ NMR (101 MHz, $CDCl_3$, 298 K)

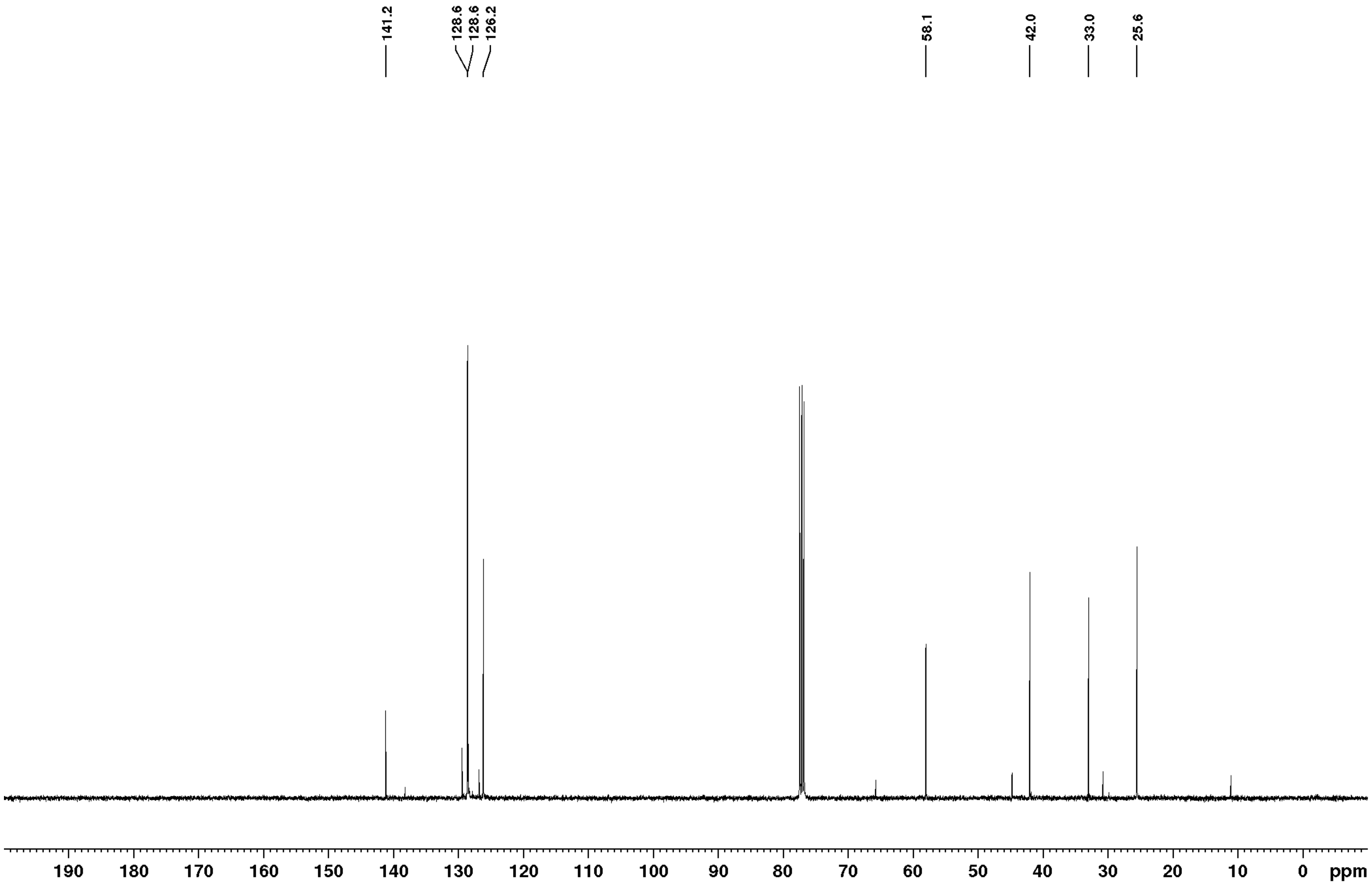

Figure S59. 5-Chlorohexyl pivalate (3a’): ^{1}H NMR spectrum (400 MHz, $CDCl_3$, 298 K)

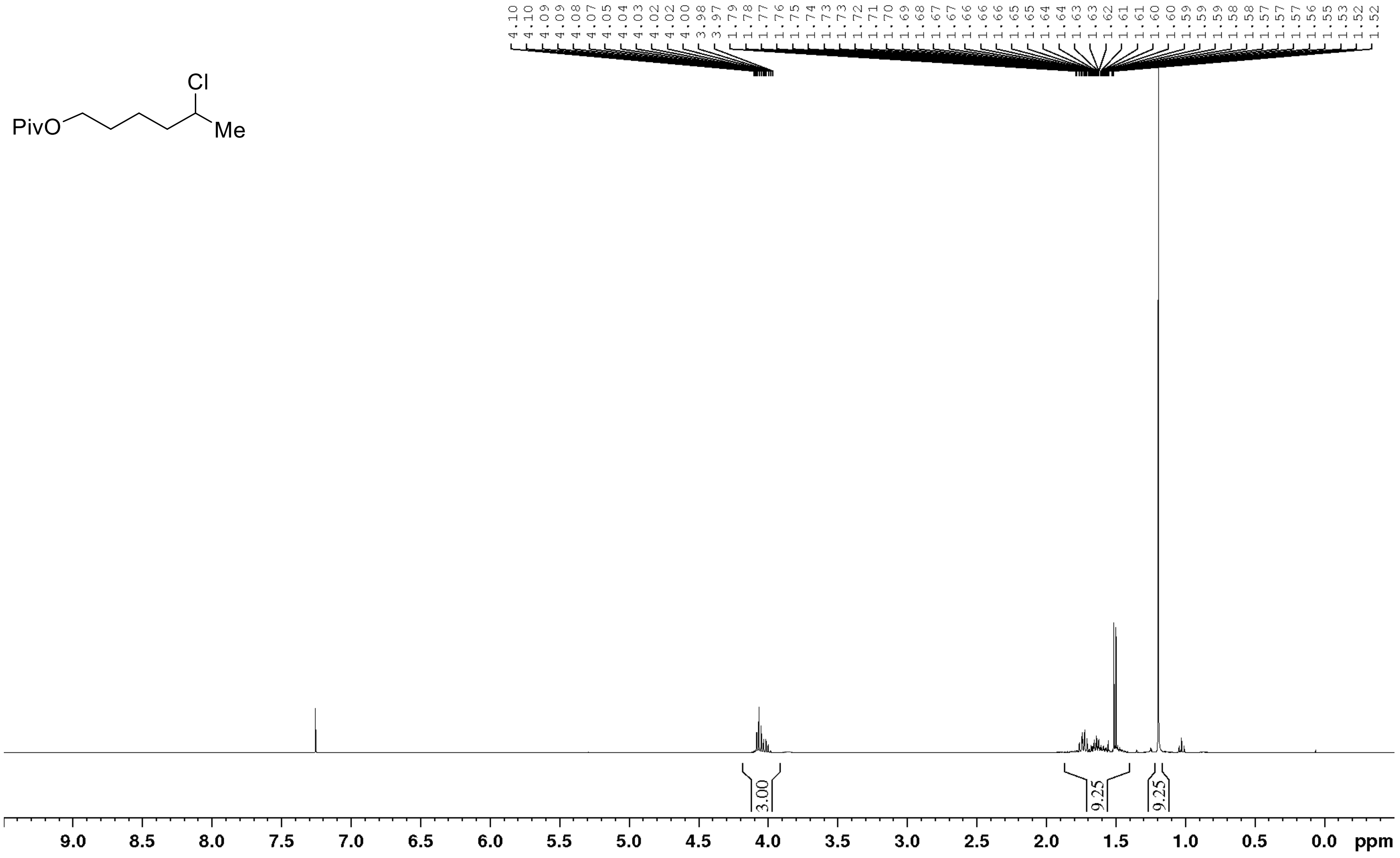

Figure S60. 5-Chlorohexyl pivalate (3a'): $^{13}C\{^1H\}$ NMR (101 MHz, $CDCl_3$, 298 K)

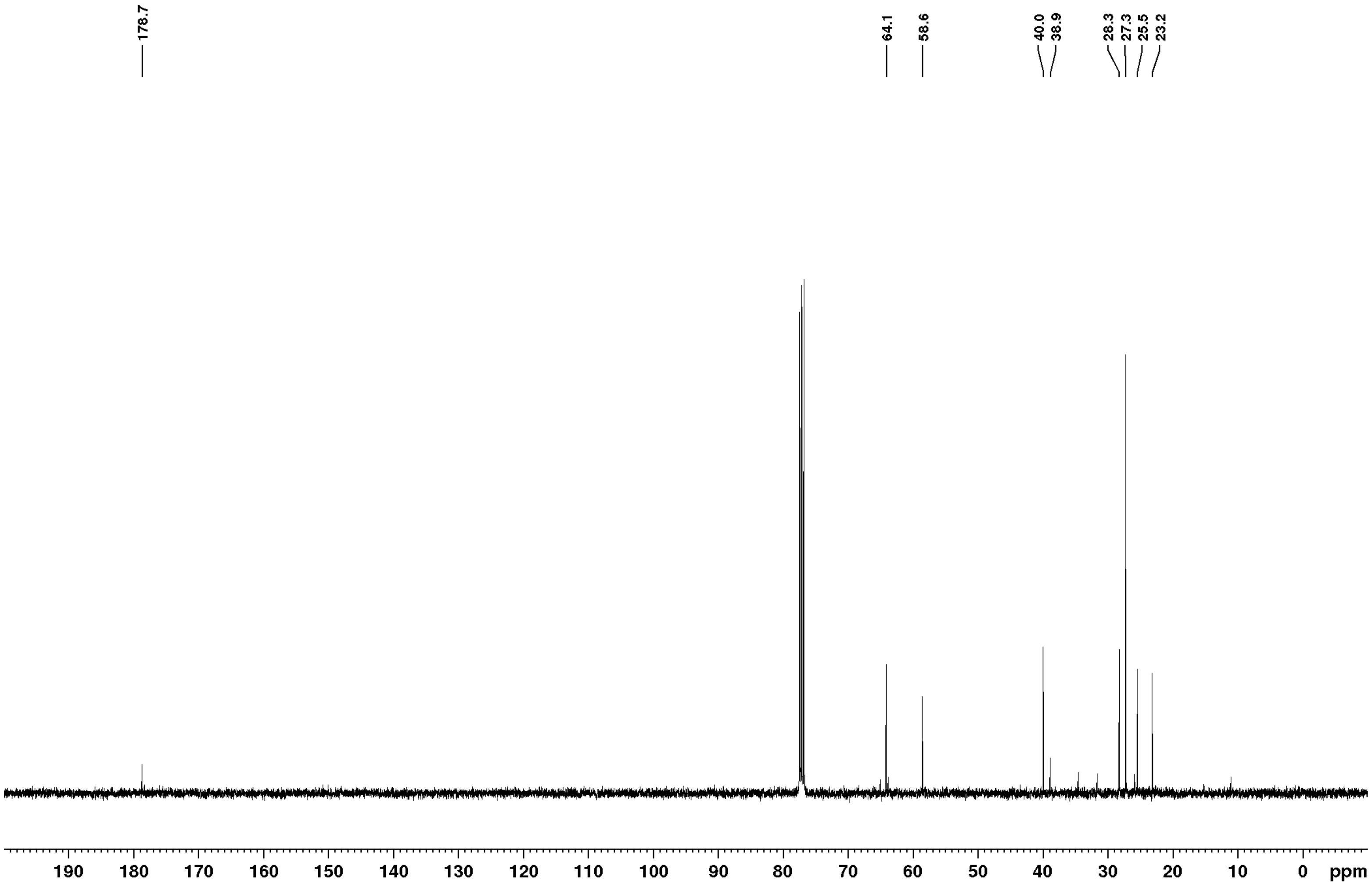

Figure S61. 1-Chloro-3-(1-chloroethyl)benzene (3b'): ^{1}H NMR spectrum (400 MHz, $CDCl_3$, 298 K)

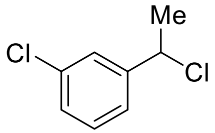

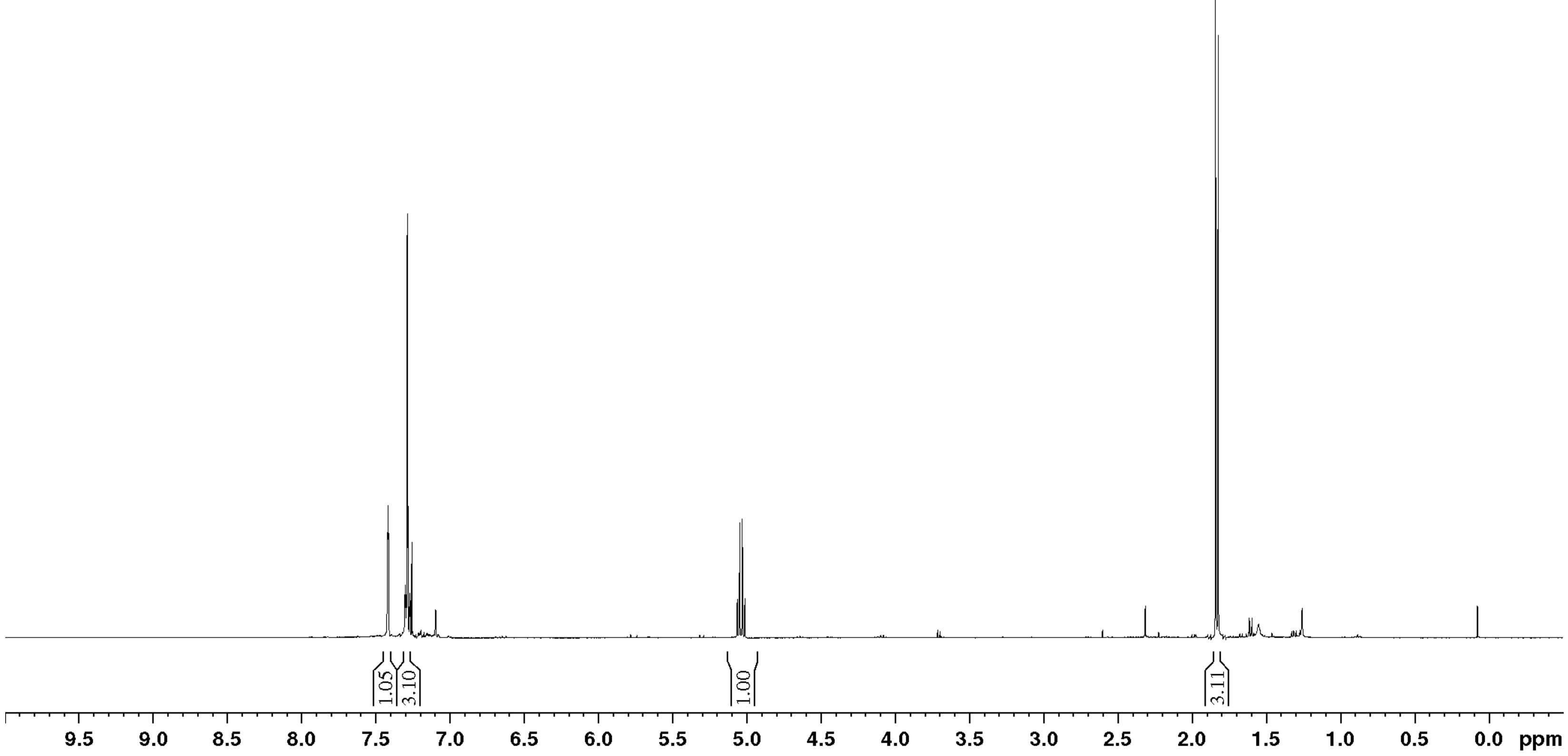

Figure S62. 1-Chloro-3-(1-chloroethyl)benzene (3b'): $^{13}C\{^1H\}$ NMR (101 MHz, $CDCl_3$, 298 K)

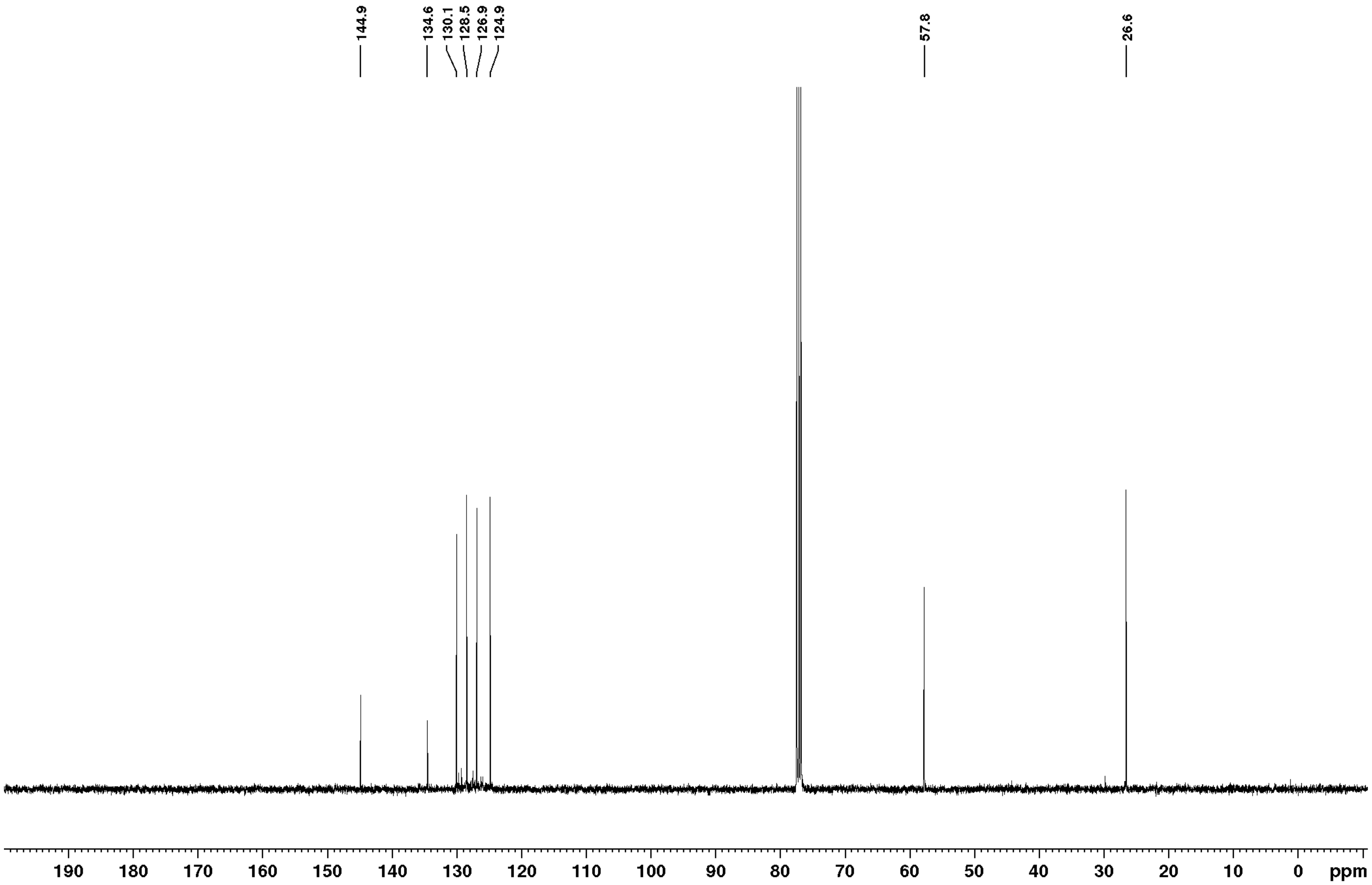

Figure S63. (*E/Z*)-(1-Chloroprop-1-en-1-yl)benzene [(*E*/*Z*)-7a]: ^{1}H NMR spectrum (400 MHz, $CDCl_3$, 298 K)

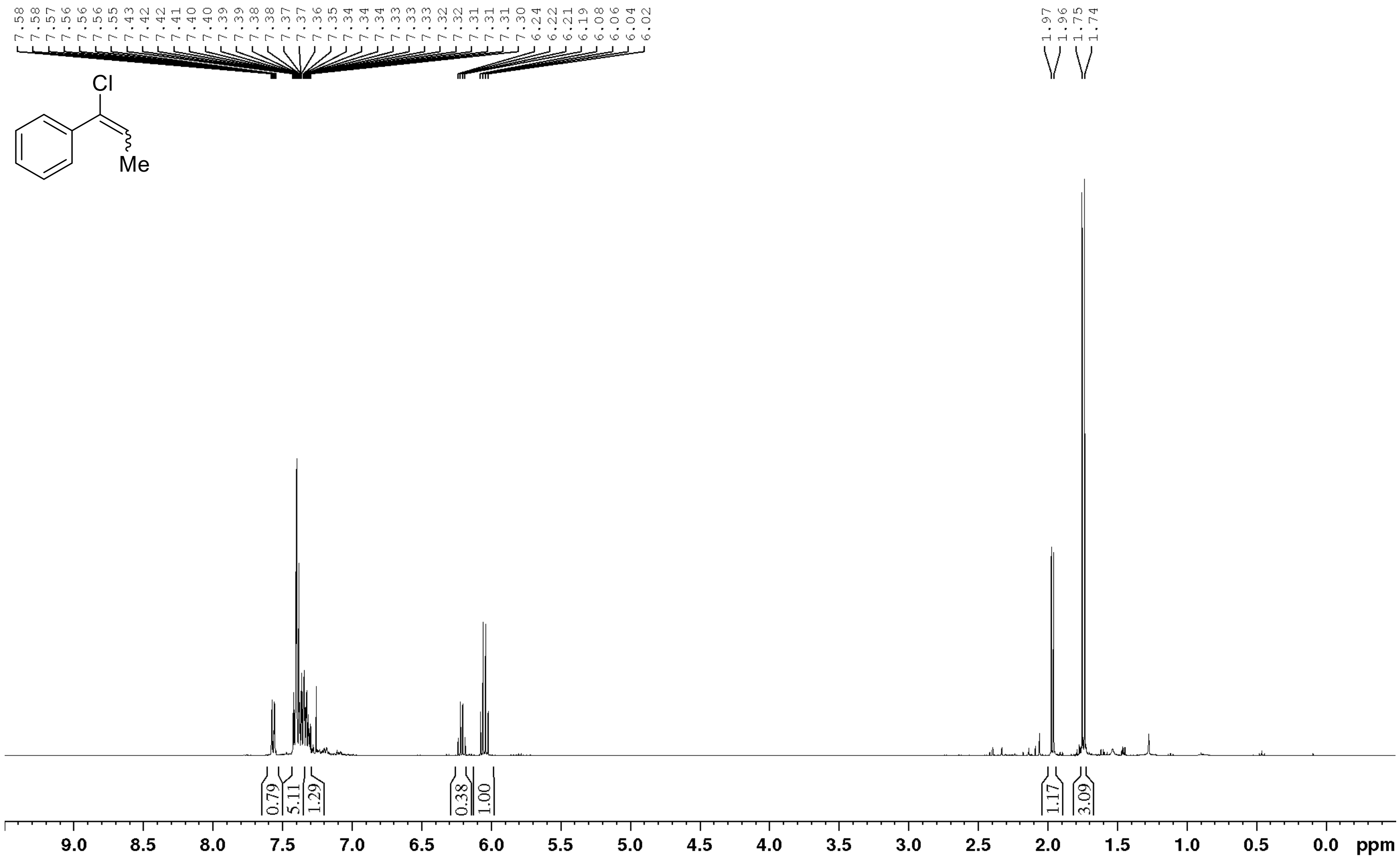

Figure S64. (*E/Z*)-(1-Chloroprop-1-en-1-yl)benzene [(*E*/*Z*)-7a]: $^{13}C\{^1H\}$ NMR (101 MHz, $CDCl_3$, 298 K)

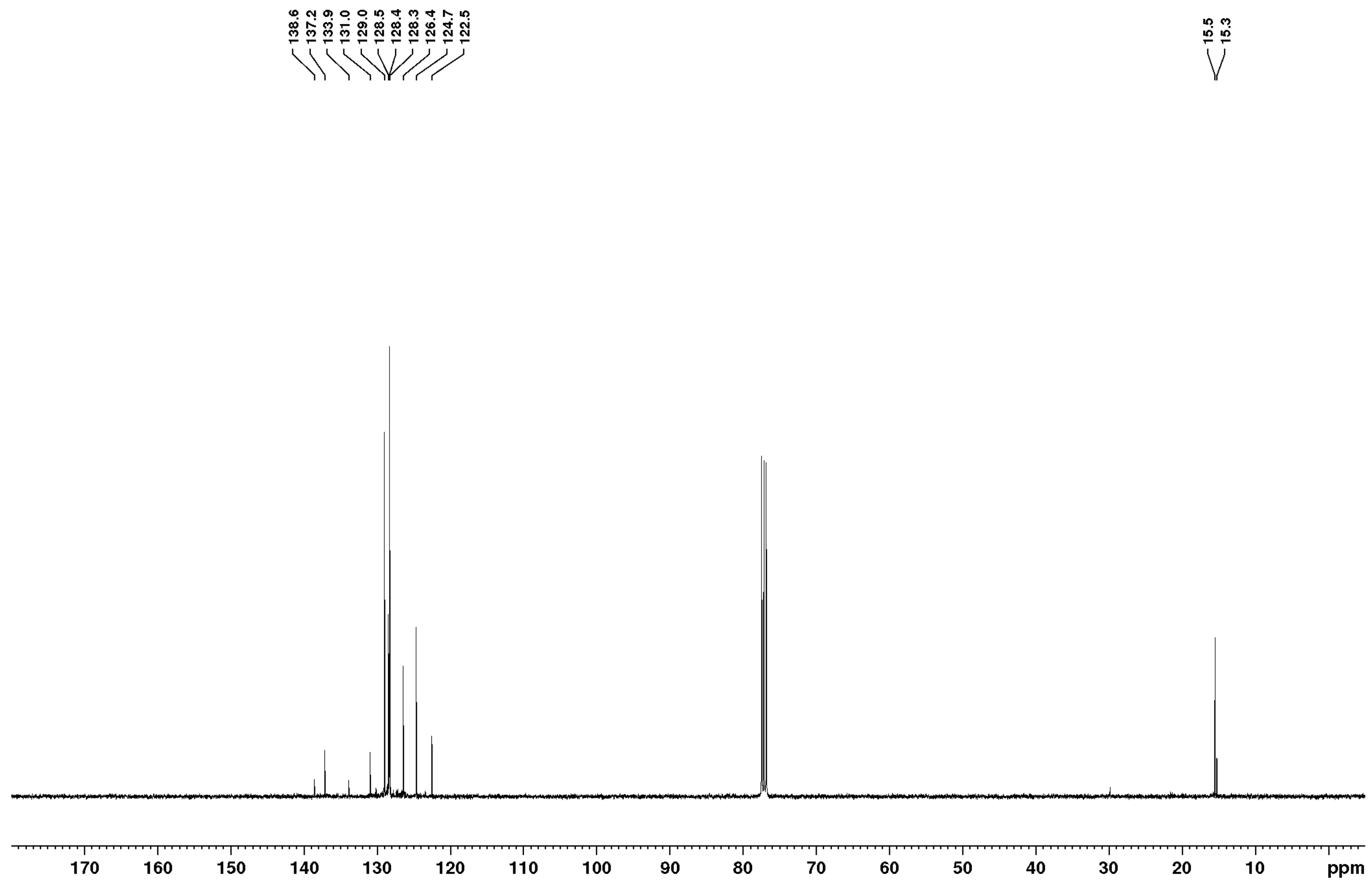

Figure S65. (*E/Z*)-(1-Chloropent-1-en-1-yl)benzene [(*E*/*Z*)-7b]: ^{1}H NMR spectrum (400 MHz, $CDCl_3$, 298 K)

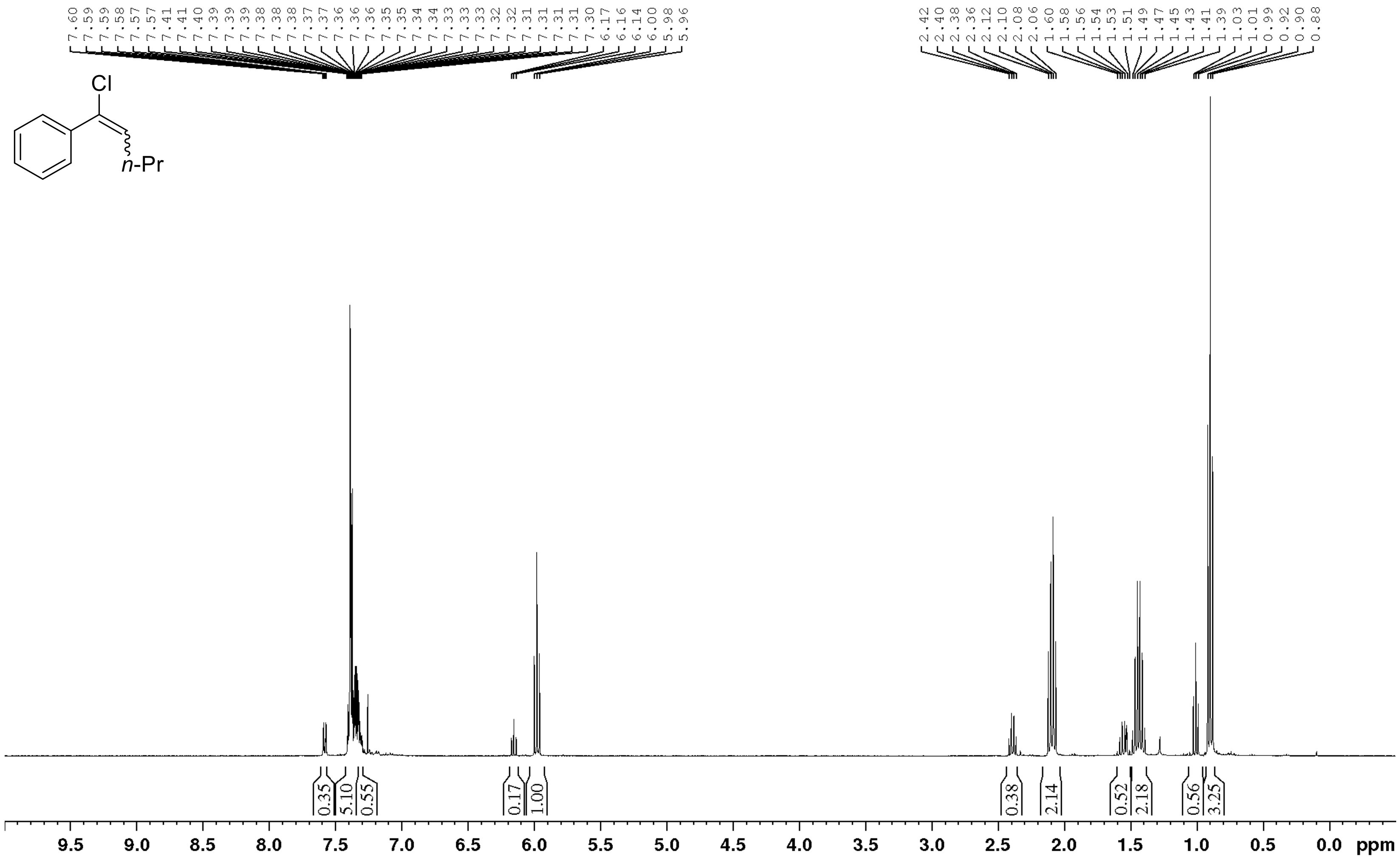

Figure S66. (*E*/*Z*)-(1-Chloropent-1-en-1-yl)benzene [(*E*/*Z*)-7b]: $^{13}C\{^{1}H\}$ NMR (101 MHz, $CDCl_3$, 298 K)

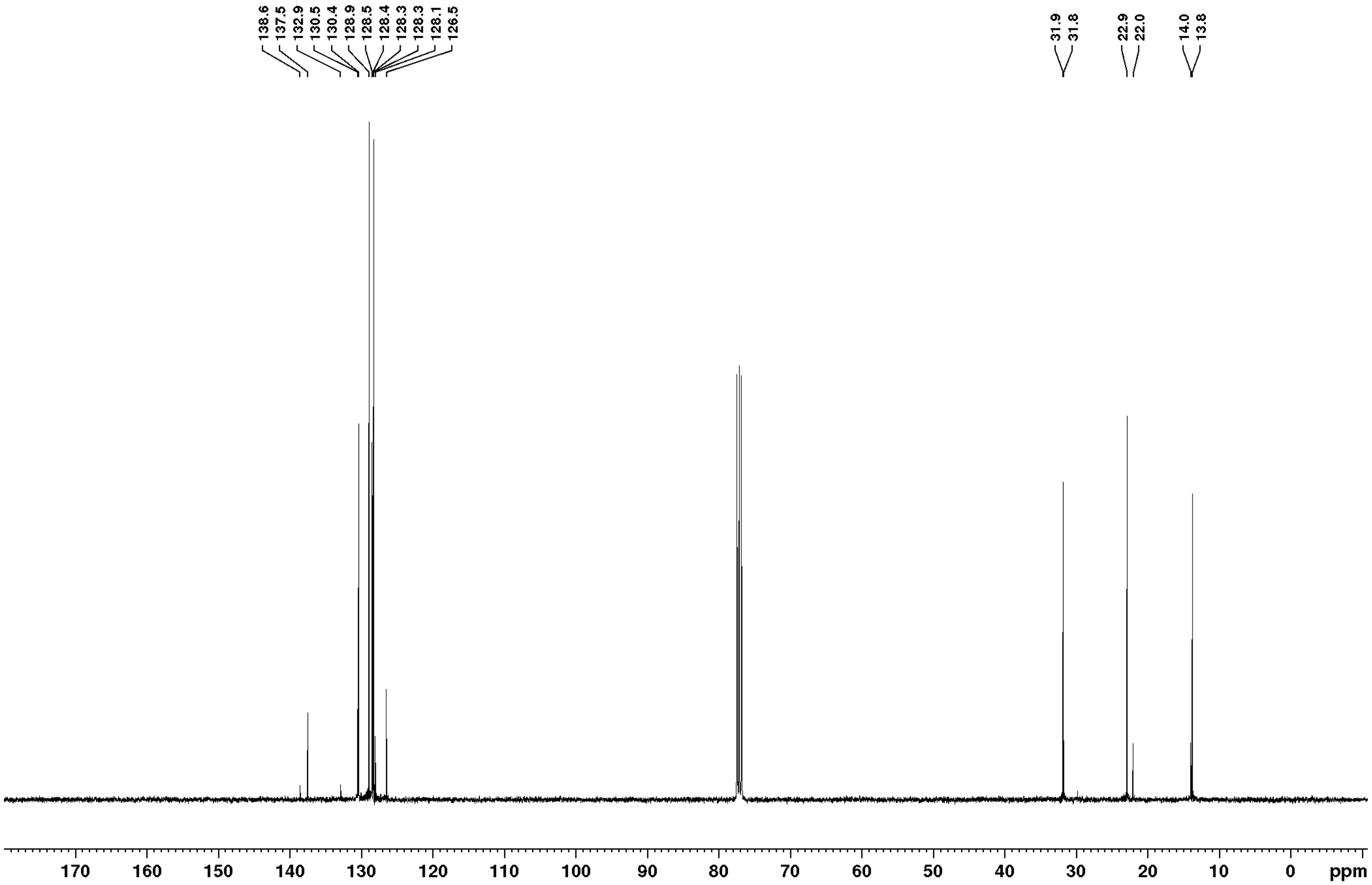

Figure S67. (*E*)-(1-Chloroethene-1,2-diyl)dibenzene [(*E*)-7c]: ^{1}H NMR spectrum (400 MHz, $CDCl_3$, 298 K)

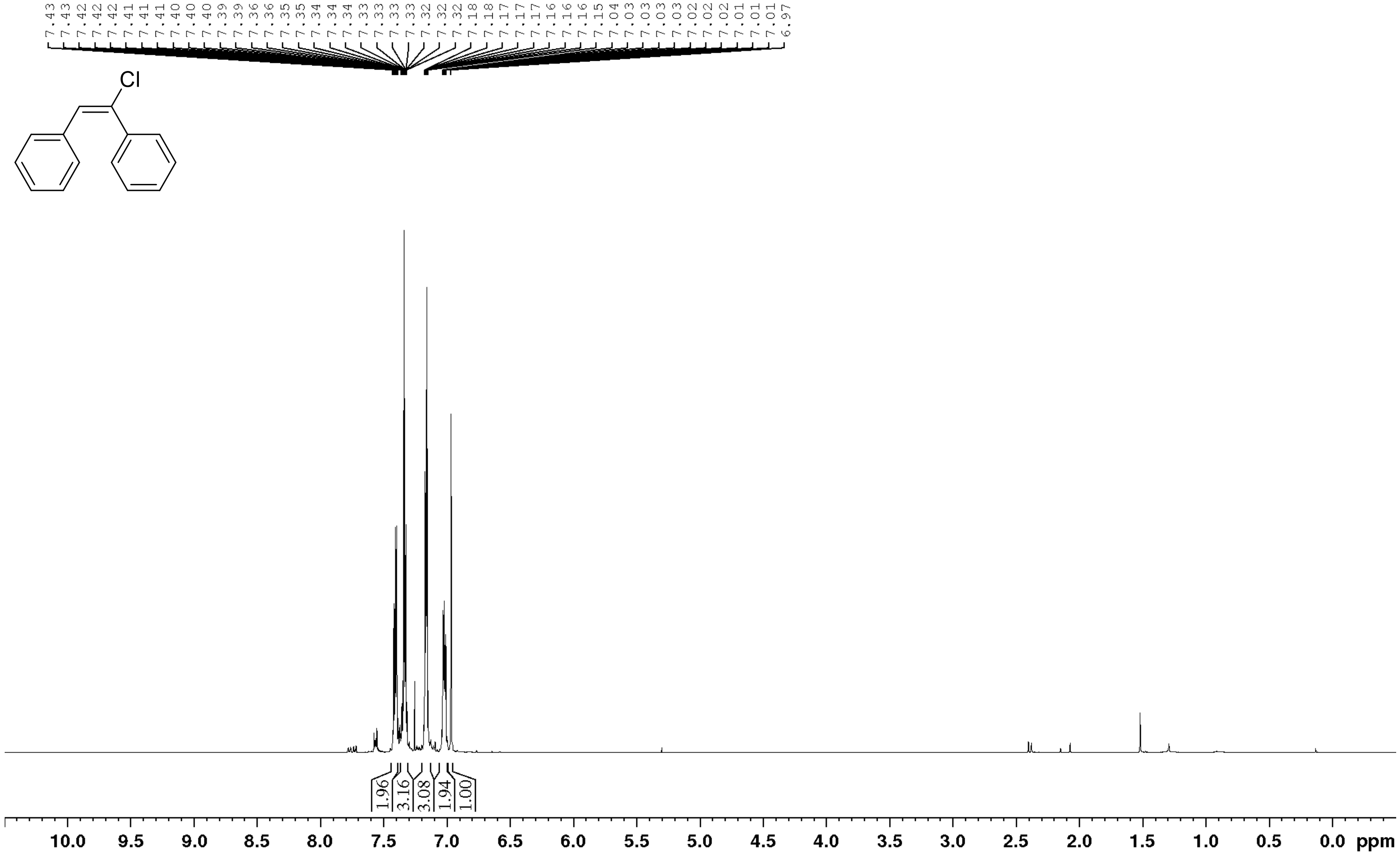

Figure S68. (*E*)-(1-Chloroethene-1,2-diyl)dibenzene [(*E*)-7c]: $^{13}C\{^{1}H\}$ NMR (101 MHz, $CDCl_3$, 298 K)

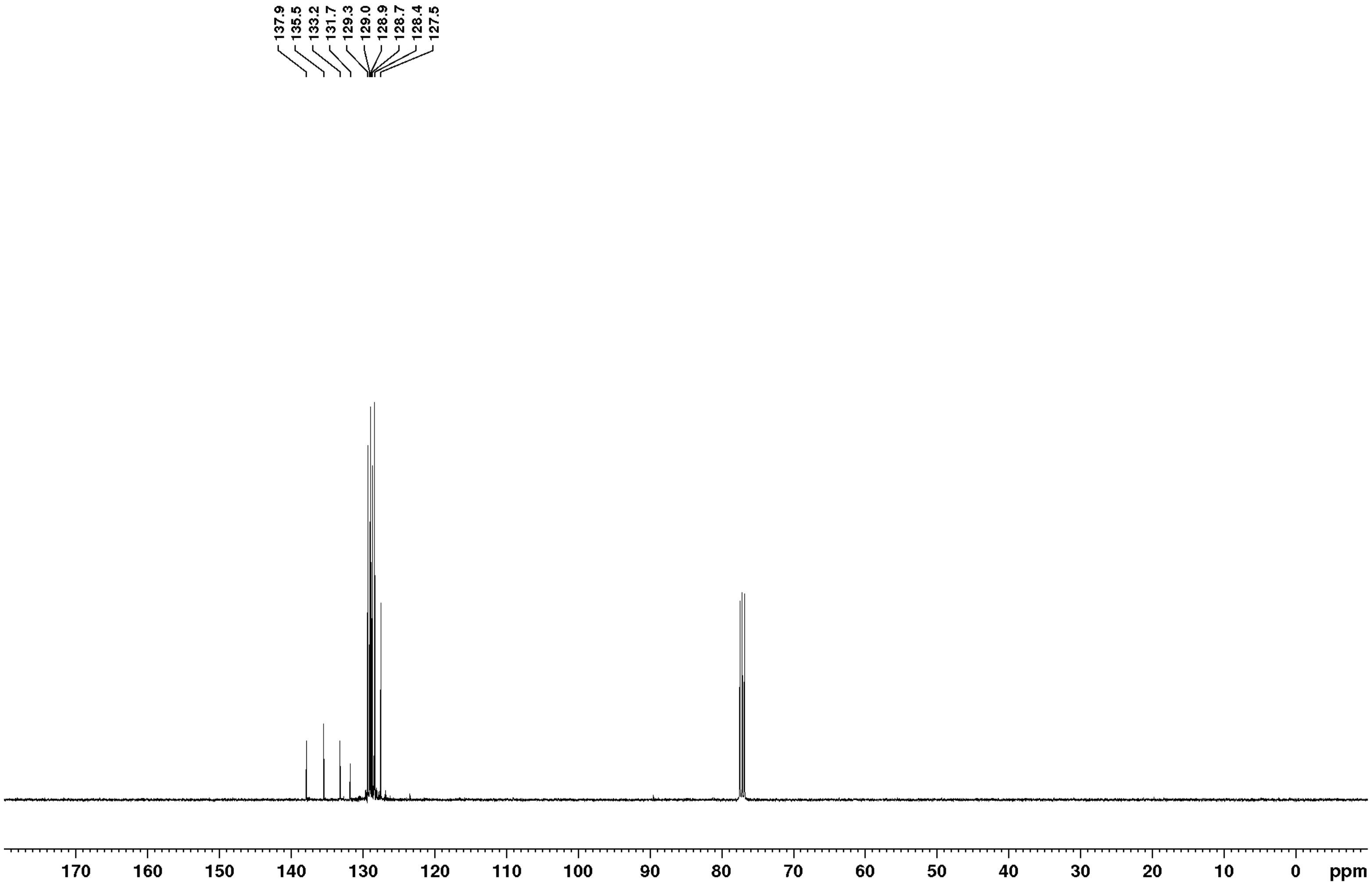

Figure S69. (*E*/*Z*)-4,4'-(1-Chloroethene-1,2-diyl)bis(methylbenzene) [(*E*/*Z*)-7d]: ^{1}H NMR spectrum (500 MHz, $CDCl_3$, 298 K)

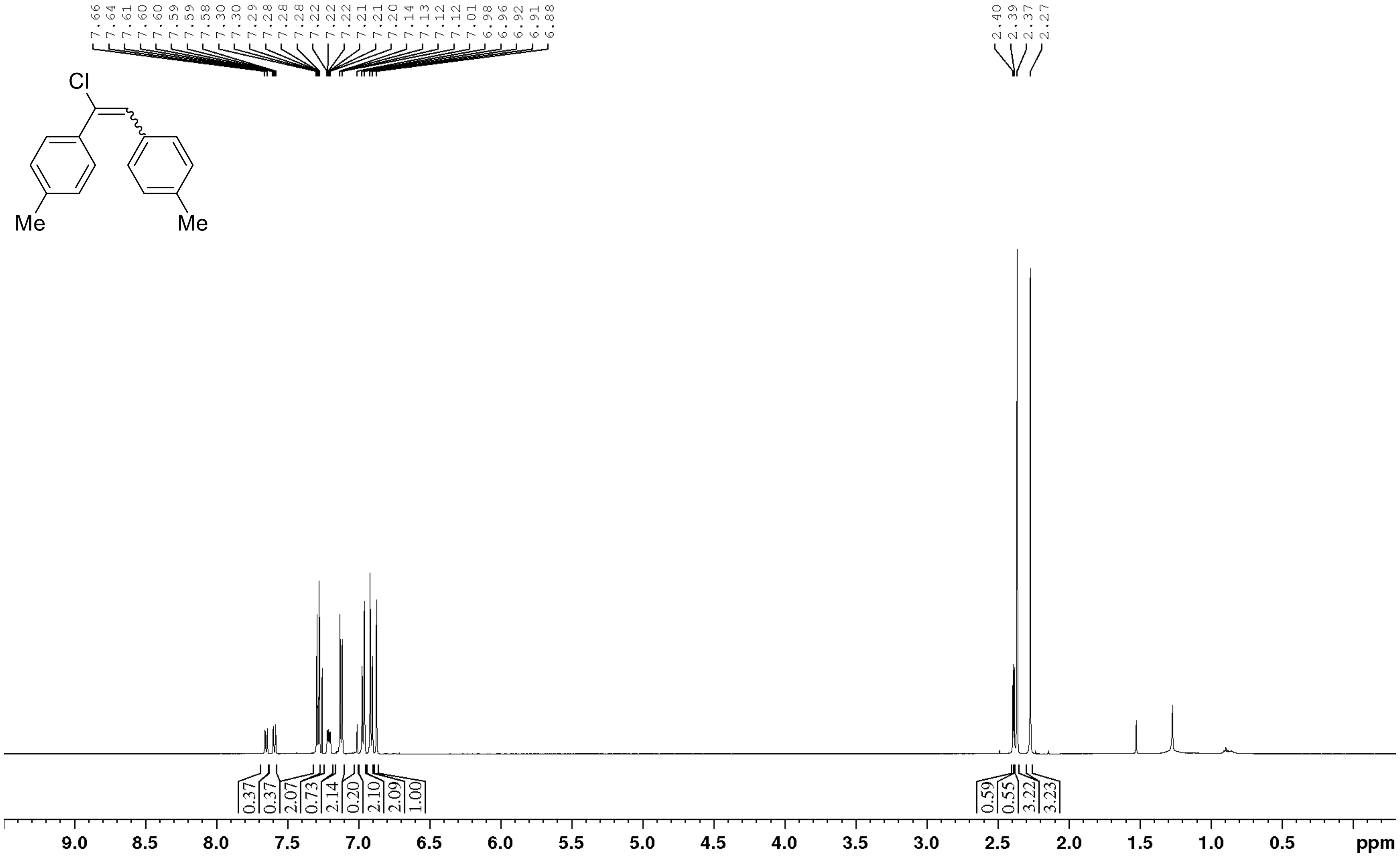

Figure S70. (*E*/*Z*)-4,4'-(1-Chloroethene-1,2-diyl)bis(methylbenzene) [(*E*/*Z*)-7d]: $^{13}C\{^1H\}$ NMR (125 MHz, $CDCl_3$, 298 K)

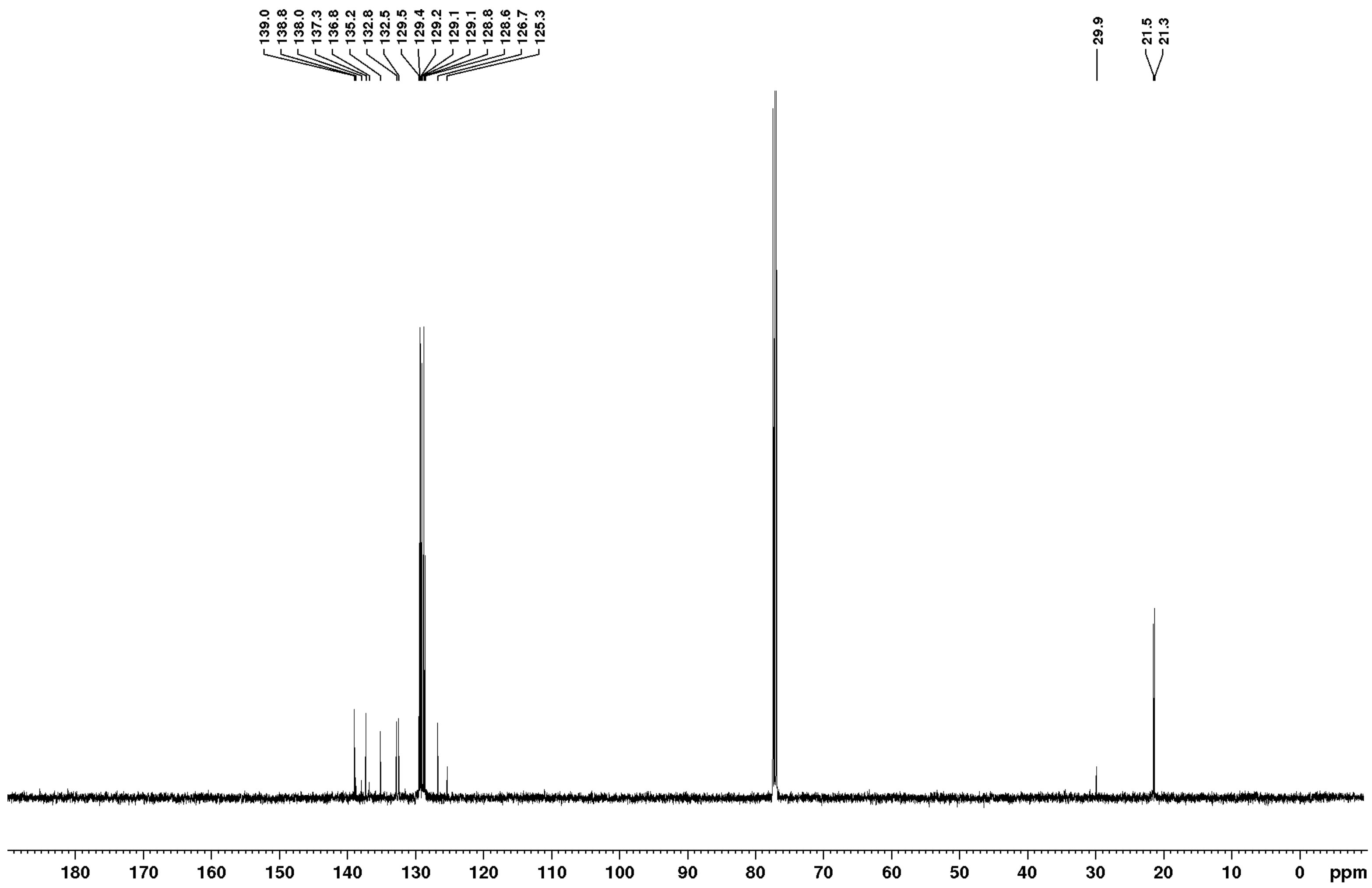

7 References

[S1] P. R. K. Harris, E. D. Becker, S. M. Cabral de Menezes, R. Goodfellow, P. Granger, *Pure Appl. Chem.* **2001**, *73*, 1795–1818.

[S2] D. Kuck, J. Schneider, H. F. Grutzmacher, *J. Chem. Soc. Perkin Trans. 2* **1985**, 689–696.

[S3] A. F. Bella, A. M. Z. Slawin, J. C. Walton, *J. Org. Chem.* **2004**, *69*, 5926–5933.

[S4] T. Krüger, K. Vorndran, T. Linker, *Chem. Eur. J.* **2009**, *15*, 12082–12091.

[S5] T. Horibe, Y. Tsuji, K. Ishihara, *ACS Catal.* **2018**, *8*, 6362–6366.

[S6] C. M. R. Volla, D. Marković, S. R. Dubbaka, P. Vogel, *Eur. J. Org. Chem.* **2009**, 6281–6288.

[S7] C. Tabélé, C. Curti, N. Primas, Y. Kabri, V. Remusat, P. Vanelle, *Synthesis* **2015**, *47*, 3339–3346.

[S8] C. Dong, L. Zhang, X. Xue, H. Li, Z. Yu, W. Tang, L. Xu, *RSC Adv.* **2014**, *4*, 11152–11158.

[S9] M. Davi, H. Lebel, *Org. Lett.* **2009**, *11*, 41–44.

[S10] H. Lebel, D. Guay, V. Paquet, K. Huard, *Org. Lett.* **2004**, *6*, 3047–3058.

[S11] N. Millius, G. Lapointe, P. Renaud, *Molecules* **2019**, *24*, 4184.

[S12] M. Fujiu, K. Negishi, J. Guang, P. G. Williard, S. Kuroki, K. Mikami, *Dalton Trans.* **2015**, *44*, 19464–19468.

[S13] P. A. Cleary, K. A. Woerpel, *Org. Lett.* **2005**, *7*, 2478–2481.

[S14] S. W. Lardy, V. A. Schmidt, *J. Am. Chem. Soc.* **2018**, *140*, 12318−12322.

[S15] L. Xu, Z. Liu, W. Dong, J. Song, M. Mao, J. Xu, H. Ren, *Org. Biomol. Chem.* **2015**, *13*, 6333–6337.

[S16] H. Albright, H. L. Vonesh, C. S. Schindler, *Org. Lett.* **2020**, *22*, 3155–3160.

[S17] S. Skvorcova, A. Jirgensons, *Org. Lett.* **2017**, *19*, 2478–2481.

[S18] W. B. Reid, J. J. Spillane, S. B. Krause, D. A. Watson, *J. Am. Chem. Soc.* **2016**, *138*, 5539−5542.

[S19] K. Park, G. Bae, J. Moon, J. Choe, K. H. Song, S. Lee, *J. Org. Chem.* **2010**, *75*, 6244–6251.

[S20] C.-H. Lee, S.-M. Lee, B.-H. Min, D.-S. Kim, C.-H. Jun, *Org. Lett.* **2018**, *20*, 2468–2471.

[S21] B. Gaspar, E. M. Carreira, *Angew. Chem. Int. Ed.* **2008**, *47*, 5758–5760.

[S22] F. T. Schevenels, M. Shen, S. A. Snyder, *J. Am. Chem. Soc.* **2017**, *139*, 6329–6337.

[S23] L. Han, J.-B. Xia, L. You, C. Chen, *Tetrahedron* **2017**, *73*, 3696–3701.

[S24] P. J. Kropp, S. D. Crawford, *J. Org. Chem.* **1994**, *59*, 3102–3112.

[S25] S. Dérien, H. Klein, C. Bruneau, *Angew. Chem. Int. Ed.* **2015**, *54*, 12112–12115.

[S26] P. Yu, A. Bismuto, B. Morandi, *Angew. Chem. Int. Ed.* **2020**, *59*, 2904–2910.
